# Quantitative CT imaging and radiation-absorbed dose estimations of ^166^Ho microspheres: paving the way for clinical application

**DOI:** 10.1186/s41747-024-00511-8

**Published:** 2024-10-14

**Authors:** Chiron Morsink, Nienke Klaassen, Gerrit van de Maat, Milou Boswinkel, Alexandra Arranja, Robin Bruggink, Ilva van Houwelingen, Irene Schaafsma, Jan Willem Hesselink, Frank Nijsen, Bas van Nimwegen

**Affiliations:** 1 Department of Clinical Sciences, Faculty of Veterinary Medicine, Utrecht University, PO Box 80154, 3508 TD Utrecht, The Netherlands; 2 Department of Medical Imaging, Radboud Institute for Health Sciences, Radboud University Medical Center, PO Box 9101, 6500 HB Nijmegen, The Netherlands; 3 Quirem Medical B.V., 8418 AH Deventer, The Netherlands; 4 3D Lab, Radboud University Medical Center, PO Box 9101, 6500 HB Nijmegen, The Netherlands

**Keywords:** Brachytherapy, Dosimetry, Holmium-166, Monte Carlo method, Tomography (x-ray computed)

## Abstract

**Background:**

Microbrachytherapy enables high local tumor doses sparing surrounding tissues by intratumoral injection of radioactive holmium-166 microspheres (^166^Ho-MS). Magnetic resonance imaging (MRI) cannot properly detect high local Ho-MS concentrations and single-photon emission computed tomography has insufficient resolution. Computed tomography (CT) is quicker and cheaper with high resolution and previously enabled Ho quantification. We aimed to optimize Ho quantification on CT and to implement corresponding dosimetry.

**Methods:**

Two scanners were calibrated for Ho detection using phantoms and multiple settings. Quantification was evaluated in five phantoms and seven canine patients using subtraction and thresholding including influences of the target tissue, injected amounts, acquisition parameters, and quantification volumes. Radiation-absorbed dose estimation was implemented using a three-dimensional ^166^Ho specific dose point kernel generated with Monte Carlo simulations.

**Results:**

CT calibration showed a near-perfect linear relation between radiodensity (HU) and Ho concentrations for all conditions, with differences between scanners. Ho detection during calibration was higher using lower tube voltages, soft-tissue kernels, and without a scanner detection limit. The most accurate Ho recovery in phantoms was 102 ± 11% using a threshold of mean tissue HU + (2 × standard deviation) and in patients 98 ± 31% using a 100 HU threshold. Thresholding allowed better recovery with less variation and dependency on the volume of interest compared to the subtraction of a single HU reference value. Corresponding doses and histograms were successfully generated.

**Conclusion:**

CT quantification and dosimetry of ^166^Ho should be considered for further clinical application with on-site validation using radioactive measurements and intra-operative Ho-MS and dose visualizations.

**Relevance statement:**

Image-guided holmium-166 microbrachytherapy currently lacks reliable quantification and dosimetry on CT to ensure treatment safety and efficacy, while it is the only imaging modality capable of quantifying high *in vivo* holmium concentrations.

**Key Points:**

- Local injection of ^166^Ho-MS enables high local tumor doses while sparing surrounding tissue.
- CT enables imaging-based quantification and radiation-absorbed dose estimation of concentrated Ho *in vivo*, essential for treatment safety and efficacy.
- Two different CT scanners and multiple acquisition and reconstruction parameters showed near-perfect linearity between radiodensity and Ho concentration.
- The most accurate Ho recoveries on CT were 102 ± 11% in five phantoms and 98 ± 31% in seven canine patients using thresholding methods.
- Dose estimations and volume histograms were successfully implemented for clinical application using a dose point kernel based on Monte Carlo simulations.

**Graphical Abstract:**

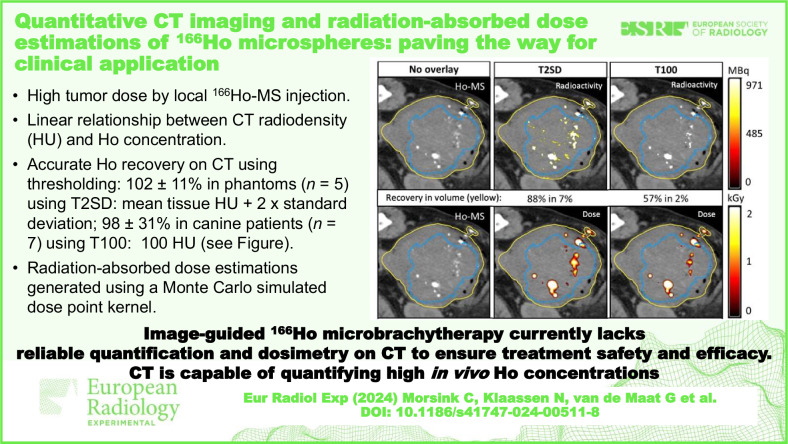

## Background

Solid tumors are mostly treated using a combination of surgery and radiotherapy [1–4]. However, these options are not always feasible, may lead to side effects, and lack efficacy in various tumor types [5–7]. Holmium-166 (^166^Ho) microbrachytherapy is a minimally invasive treatment option that enables tumor destruction by intra-tumoral injections of radioactive ^166^Ho microspheres (^166^Ho-MS) [8–15]. ^166^Ho emits high-energy beta radiation (*E*_β, max_ = 1.77 MeV, 49.9%; 1.85 MeV, 48.8%) with a limited soft-tissue penetration depth (90% dose delivery in the first 3 mm tissue), enabling a high local dose while sparing surrounding tissues [13, 16–18].

^166^Ho microbrachytherapy was shown to be effective with minimal side effects in veterinary patients, resulting in mean tumor volume decreases of up to 83% [9, 10, 12, 15]. Patient quarantine after treatment was relatively short because of low energy gamma radiation (*E*_γ_ = 0.08 MeV, 6.6%) and the short ^166^Ho half-life (*t*_1/2_ = 26.82 h). Treatment can be performed during a single anesthesia event and be repeated if indicated [9, 12]. The feasibility of ^166^Ho microbrachytherapy was subsequently demonstrated in human patients [8]. However, intra-operative imaging-based biodistribution and dose monitoring are required to improve treatment safety and efficacy.

^166^Ho-MS can be visualized with magnetic resonance imaging (MRI), computed tomography (CT), and single-photon emission CT based on their paramagnetic characteristics, high electron density, and the emitted gamma radiation, respectively [19]. MRI is currently used during transarterial radioembolization of liver malignancies [20–24], with relatively low reported *in vivo* concentrations of ~ 0.2–0.3 mg ^166^Ho-MS per mL tissue, assuming homogeneous distribution in a relatively large liver volume through hepatic artery branches [21, 25]. In comparison, *in vivo* concentrations during ^166^Ho microbrachytherapy were between 0.5 and 100 mg per mL tissue, depending on tumor volume and dose cohort [8, 10, 12].

Quantification of relatively high local Ho-MS concentrations using MRI is complicated because the minimal echo times and intervals are relatively long (~ 1 ms) in multiple gradient-echo sequences on clinical scanners [26]. In other words, signal decay occurs too rapidly for accurate sampling [26] due to high local Ho-MS concentrations. While these high local concentrations can be estimated using a postprocessing method [26], CT may be better suited for the quantification of high Ho-MS amounts [27–30].

CT has high anatomical reference and resolution for the detection of Ho-MS [19], it is generally quicker and cheaper compared to MRI and single-photon emission CT, has fewer restrictions (no magnetic field), and is more widely available [31–34]. The feasibility of Ho quantification on CT has previously been demonstrated within our group, using a selected threshold value based on linear HU value increases for higher Ho concentrations [29]. As outlined by the authors, Ho quantification could be further optimized by improving acquisition protocols and adapting to the target tissue. In addition, the radiation-absorbed tissue doses were not modeled, which is essential to monitor the safety and efficacy of radionuclide therapy.

In the present work, we aimed to optimize Ho quantification on CT, generate corresponding radiation-absorbed dose estimations, and provide recommendations for clinical implementation. We evaluated Ho detection efficacy using phantoms and multiple CT parameters on two different clinical scanners, we implemented quantification and dosimetry in dedicated software using multiple thresholding and subtraction methods, and we evaluated quantification efficacy and feasibility of dosimetry in phantoms and canine patients using one scanner.

## Methods

### CT calibration

Two phantoms were prepared to determine the relationship between CT radiodensity in HU and non-radioactive Ho concentrations: (i) a Ho chloride (HoCl) phantom with a large range of concentrations for extended evaluation; and (ii) a Ho-MS phantom with a smaller matching range consistent with clinical application.

#### HoCl phantom preparation

The HoCl phantom contained 19 tubes ranging from 0.0 to 129.0 mg Ho/mL (Supplementary Table S3). A stock solution was prepared of 150 mg/mL Ho (III) chloride hexahydrate (HoCl_3_·6H_2_O, 43% Ho) in sterile water (Versylene, Fresenius Kabi, B.V., Huis ter Heide, The Netherlands), which was diluted for each concentration. Two 15-mL tubes (Polystyrene Centrifuge Tube, Falcon™) were prepared per concentration, one for scanning and one to determine the Ho concentration using Inductively Coupled Plasma Optical Emission Spectrometry. From each tube, 150 µL was diluted 50 or 100 times in 2% nitric acid and measured at two different wavelengths (345.6 and 347.4 nm) using a spectrometer (Optima 8,000, PerkinElmer, Inc., Waltham, MA, USA; Sapphire injector, glass nebulizer). The spectrometry calibration solutions were prepared by dissolving Ho Inductively Coupled Plasma standards in 2% nitric acid, and the maximum measurement error was 2%.

#### Ho-MS phantom preparation

The Ho-MS phantom contained nine tubes ranging from 0.0 to 9.7 mg Ho/mL (Supplementary Table S3). For each concentration, a stock solution was prepared of 30 mL of 1.3% agarose (Agar powder (Product code 20768292), VWR Chemicals, PA, USA), 15 mL of 0.1% poloxamer 188 (Pluronic F-68, Sigma-Aldrich, Zwijndrecht, The Netherlands) in sterile water, and the required amount (mg) of Ho poly(L-lactic acid) MS (Quirem Medical B.V., Deventer, The Netherlands; 19.7% Ho; Supplementary Table S3) prepared and weighed as previously described [35]. The agar was dissolved in sterile water and heated to 90 °C for 10 min. Poloxamer 188 was added, and the solution was slowly cooled to 40 °C while stirring. Each solution was poured into the tubes gently but quickly to prevent air bubble formation and placed in ice to speed up hardening and prevent Ho-MS sedimentation.

#### CT acquisition

A SOMATOM Definition AS (Siemens Medical Solutions USA, Inc., PA, USA) and an Aquilion ONE (Canon Medical Systems USA, Inc., CA, USA) were used for Ho calibration and to compare results. The Siemens scanner is a 12-bit system with a HU scale ranging from -1,024 to 3,071 HU, whereas the Canon scanner is a 16-bit system ranging from -32,768 to 32,767 HU [36, 37]. The Hounsfield scale of tissue density is open-ended; however, the scanners are limited to these different respective minimum and maximum HU values for measuring low- and high-density materials.

**Fig. 1:**
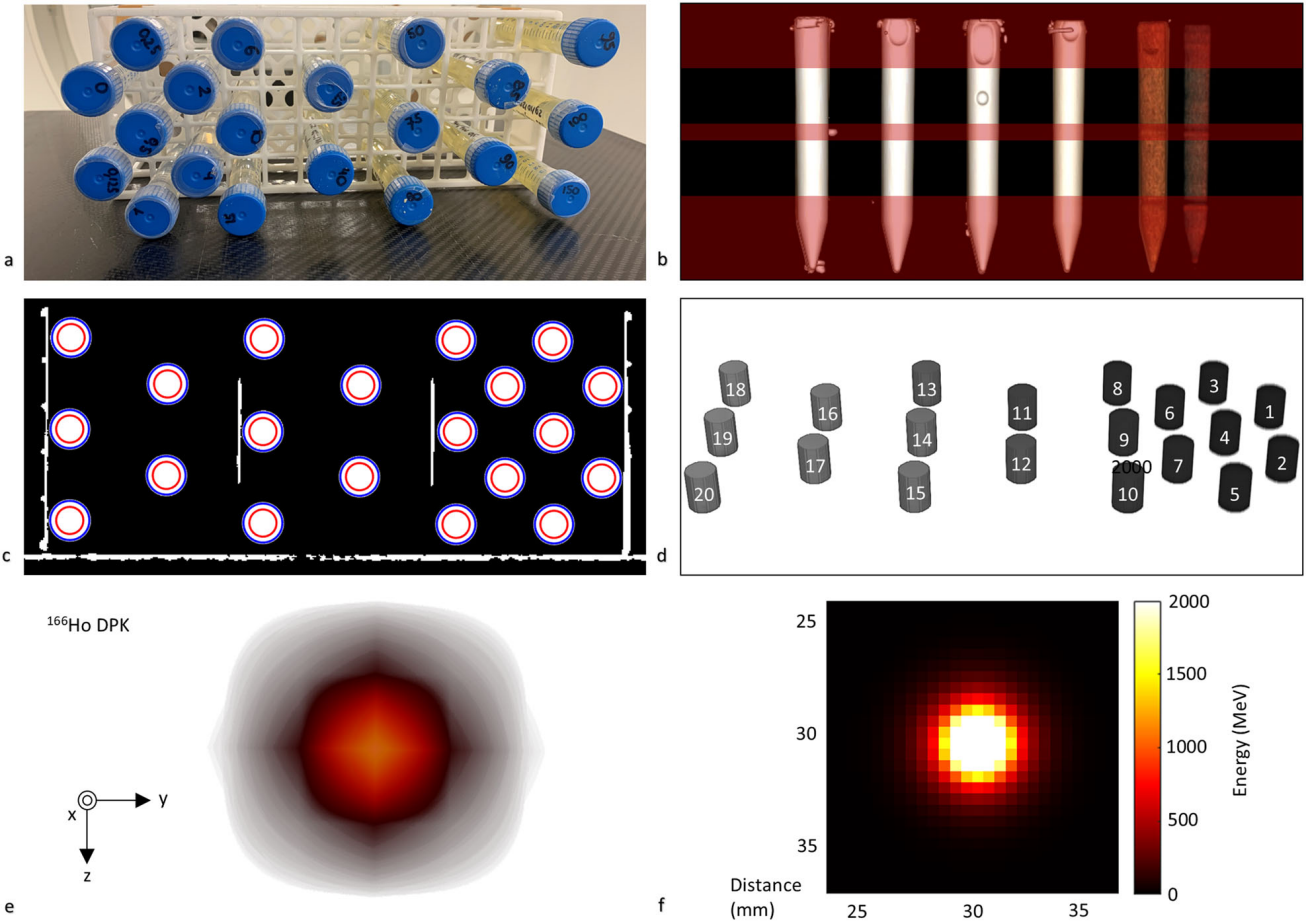
CT calibration and the generated DPK used for Ho quantification and ^166^Ho radiation-absorbed dose estimations. **a** Craniocaudal tube positioning of a HoCl phantom with increasing Ho concentrations for calibration. **b** 3D visualization showing decreased attenuation for lower Ho concentrations (left to right) with removed slices (transparent red planes) containing the tapered tube bottoms and caps, air, and/or the positioning racket. **c** Cylindrical masks around the tube edges (blue lines) and calibration area (red lines). **d** Segmented cylindrical volumes (3.0 cm^3^) used for radiodensity measurements from low (1) to high (20) Ho concentration. Tube 10 (15 mg/mL) was scanned but not used due to a preparation error. **e** 3D geometry of the ^166^Ho DPK used for radiation-absorbed dose estimations on CT images. **f** Energy distribution of the generated ^166^Ho DPK measuring 61 × 61 × 61 mm^3^ with the source located in its center. 3D, Three-dimensional; CT, Computed tomography; DPK, Dose point kernel; HoCl, Holmium chloride

The calibration tubes were placed in metal-free rackets positioned horizontally and parallel to the CT table to obtain cross-sectional images (Fig. 1a). Both phantoms were first scanned using the Siemens scanner, in free air using 81 combinations of acquisition parameters (Table 1) based on diagnostic acquisition protocols of soft tissue and brain tumors at the Academic Veterinary Hospital and Radboud University Medical Center. The organ characteristic was set to the brain, the data collection diameter was 500 mm, the pitch was 1.0, and the slice increment was equal to the slice thickness. The scans were then repeated on the Canon scanner with matched dose settings using the resulting CT dose index volume (mGy).

**Table 1:** Acquisition parameters for Ho calibration and quantification

Scan object	Scanner	Tube kilovoltage (kVp)	Exposure (mAs)	Slice thickness (mm)	Reconstruction kernel
Calibration phantoms	Siemens	80, 100, 120	Free, 200, 400	1, 2, 5	H31s, H41s, H60s
	Canon	80, 100, 120	Matched on CTDIvol	1, 2, 5	Brain, Brain + , Bone
Quantification phantoms	Siemens	80, 120	400	1	H41s
Veterinary patients	Siemens	120	400	1	H41s

Two different clinical scanners (Siemens SOMATOM Definition AS; Canon Aquilion ONE) and multiple acquisition and reconstruction parameters were used. Exposure settings between the scanners were matched based on CTDIvol (see Supplementary Table S4). *CTDIvol* CT dose index volume

#### CT data processing

CT datasets were loaded in RadiAnt^™^ (Medixant. Version 2022.1.1. Aug 17, 2022) for visual inspection (Fig. 1b) and in MATLAB (Version 9.10.0 (R2021a), The Mathworks Inc.; 2021, Natick, MA, USA) to extract HU values. CT intensities were converted to HU values by multiplying with the Rescale Slope (DICOM Tag (0028, 1053)) and adding the Rescale Intercept (DICOM Tag (0028, 1052)). Slices were removed if they contained tapered tube bottoms, caps, air bubbles, and/or the positioning racket (Fig. 1b). Outer excess slices were removed to create equal lengths of 30 mm across datasets. Cylindrical masks of 100 mm^2^ (⌀ 11 mm) were applied around each tube center leaving a circumferential margin of ~ 3 mm to the tube edge (⌀ 17 mm) (Fig. 1c) and the data was sorted by concentration (Fig. 1d). Mean HU values (HU_mean_) were calculated with standard deviations (SD), 95% confidence interval, the minimum HU value (HU_min_) and the maximum HU value (HU_max_), which were exported to a spreadsheet file (Microsoft Excel®, Microsoft Corporation, 2022).

#### Statistical analysis

Regression statistics were used to test the linearity of the relationship between HU_mean_ and Ho concentration [29]. Data points were excluded for HU_max_ values higher than the detection limit. Results consisted of the calibration intercept (m, HU) and slope (b, HU x mL/mg Ho), the correlation coefficient (R), the coefficient of determination (R square), the standard error, the *F*-statistic, and the *t*-statistics with significance at *p* < 0.05. Regression lines were forced through zero if the standard errors were larger than the corresponding intercepts [38].

### Protocol for Ho quantification and radiation-absorbed dose estimations

**Fig. 2:**
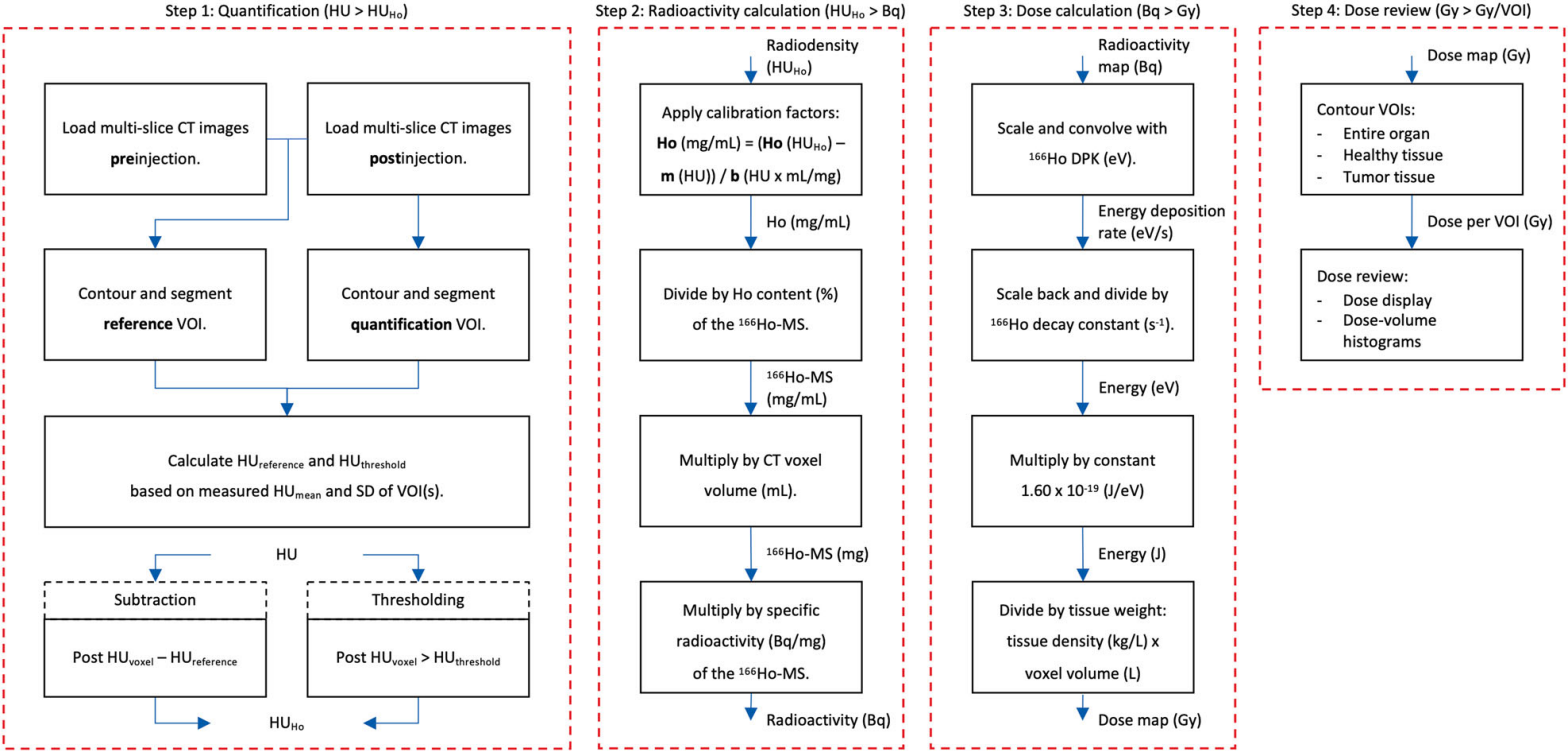
Protocol for Ho quantification and generating corresponding ^166^Ho radiation-absorbed dose estimations on CT. CT, Computed tomography; DPK, Dose point kernel; Ho-MS, Holmium microspheres; SD, Standard deviation; VOI, Volume of interest

The following protocol for ^166^Ho quantification and dose estimations (Fig. 2) was implemented in a research version of Q-Suite™ (Quirem Medical B.V.) [20, 21, 23, 25].

#### Ho quantification

Ho was quantified using multiple subtraction and thresholding methods based on baseline tissue HU (HU_reference_) in a reference volume of interest (VOI) on a preinjection CT image and increased mean HU values (HU_Ho_) in a quantification VOI containing Ho-MS on postinjection CT image.

Using subtraction, Ho was quantified by subtracting the single HU_reference_ value preinjection from individual voxel values postinjection (Eq. 1). Subtracting individual voxel values pre- and postinjection might be more accurate but CT image registration with submillimeter voxel accuracy was not available. We hypothesized that subtracting a single value from both negative and positive postinjection voxel values would result in a mean HU increase due to Ho-MS deposition. The following variations were used to analyze this and if (extremely) negative values non-representative of injected tissue could be excluded while not needing to contour around them (such as air, bile ducts, or brain ventricles):

i. results including all negative values (S −; Eq. 1);
ii. results excluding voxel values below the negative HU_reference_, assuming the minimum value of tissue without Ho-MS is zero (S; Post HU_voxel_ = 0, HU_Ho_ ≥ -HU_reference_);
iii. results excluding voxel values below zero (S + ; HU_Ho_ ≥ 0).

1 $${{\rm{Subtraction}}}\!:\,{{{\rm{HU}}}}_{{{\rm{Ho}}}}={{\rm{Post}}}\,{{{\rm{HU}}}}_{{{\rm{voxel}}}}-{{{\rm{HU}}}}_{{{\rm{reference}}}}$$

Using thresholding, Ho was quantified by setting a threshold HU value (HU_threshold_, Eq. 2) using the following patient-specific and fixed variations for postinjection voxel values:

i. HU_reference_ for comparison with subtraction (*T*);
ii. HU_reference_ with an addition of one to three times the SD, respectively (T1SD, T2SD, T3SD), based on unknown CT signal noise and likely overvalued results by only using the HU_reference_;
iii. 50 HU based on the known HU of soft tissue [39];
iv. 100 HU based on previous research [29].

In theory, thresholding could result in voxels with negative Ho concentrations for tissues with low HU_reference_ values by subtraction of a higher CT calibration intercept (Eq. 3). These negative concentrations were detected and excluded by setting the intercept (if applied) as minimal threshold.

2 $${{\rm{Thresholding}}}\!:{{{\rm{HU}}}}_{{{\rm{Ho}}}}={{\rm{Post}}}\,{{{\rm{HU}}}}_{{{\rm{voxel}}}} \, > \, {{{\rm{HU}}}}_{{{\rm{threshold}}}}$$

#### Holmium-166 radioactivity calculation

The HU_Ho_ values were converted to Ho concentrations using the intercept (if applied) and slope values from the HoCl calibration (Eq. 3) and subsequently to Ho-MS radioactivity (Eq. 4).

3 $${{{\rm{Ho}}}}({{{\rm{HU}}}}_{{{\rm{Ho}}}})	=m+b\times {{{\rm{Ho}}}}({{{\rm{mg}}}}/{{{\rm{mL}}}})\\ 	\to {{{\rm{Ho}}}}({{{\rm{mg}}}}/{{{\rm{mL}}}})=\frac{{{{\rm{Ho}}}}({{{\rm{HU}}}}_{{{\rm{Ho}}}})-m({{{\rm{HU}}}})}{b({{{\rm{HU}}}}\times {{{\rm{mg}}}}/{{{\rm{mL}}}})}$$

where $m$ is the calibration intercept and $b$ is the calibration slope.

4 $${A}_{{{\rm{voxel}}}}({{{\rm{Bq}}}})=	\frac{{{{\rm{Ho}}}}({{{\rm{mg}}}}/{{{\rm{mL}}}})\times {V}_{{{\rm{voxel}}}}\left({{{\rm{mL}}}}\right)}{{{{{\rm{Ho}}}}\; {{{\rm{content}}}}\; {{{\rm{Ho}}}}}-{{{\rm{MS}}}}\,\,\left( \% \right)} \\ 	 \times {A}_{{{{\rm{Ho}}}}-{{{\rm{MS}}}}}({{{\rm{Bq}}}}/{{{\rm{mg}}}})$$

where ${A}_{{{\rm{voxel}}}}$ is the ^166^Ho-MS radioactivity in each voxel, ${V}_{{{\rm{voxel}}}}$ is the voxel volume calculated by multiplying the pixel spacing in direction *x* by the pixel spacing in direction *y* by the slice spacing (direction *z*) with slice thickness equal to increment, and ${A}_{{{\rm{Ho}}}-{{\rm{MS}}}}$ is the ^166^Ho-MS specific radioactivity.

#### Holmium-166 radiation-absorbed dose estimations

A three-dimensional point-symmetric ^166^Ho dose point kernel (DPK) was calculated using Monte Carlo Simulations (MCNPX®, Version 2.7.0, Los Alamos National Laboratory, NM, USA) according to the method described in Medical Internal Radiation Dose Pamphlet 17 [40] and assuming a tissue density of 1.06 kg/L based on liver tissue according to International Commission on Radiation Units and Measurements Report 44 [41] (Fig. 1e, f). A 121 × 121 × 61 matrix and 0.5 × 0.5 × 1.0 mm^3^ resolution were used with the source located in its center and spatial distributions of overlapping radiation energies extracted from dose-info.radar.com (beta particles, Auger and conversion electrons, x-rays, and gamma photons) [42, 43] using default particle physics settings (energy cutoff 1 keV, photoelectric effect, and coherent photon scattering turned on, Brehmsstrahlung and x-ray production by electrons).

The voxel radioactivity map was matched to the DPK size and resolution using scaling and trilinear interpolation, they were convolved to calculate the energy deposition rates (Eq. 5) and scaled back to calculate the cumulative dose map (Eq. 6).

5 $$E\left({{\rm{eV}}}/{{\rm{s}}}\right)=A\left({{{\rm{s}}}}^{-1}\right)\otimes {{\rm{DPK}}}({{\rm{eV}}})$$

6 $${D}_{{{\rm{voxel}}}}({{\rm{Gy}}})=\frac{E({{\rm{eV}}}/{{\rm{s}}})\,}{\lambda \left({{{\rm{s}}}}^{-1}\right)}\times \frac{1.60\times {10}^{-19}({{\rm{J}}}/{{\rm{eV}}})}{\rho ({{\rm{kg}}}/{{\rm{L}}})\times {V}_{{{\rm{voxel}}}}\left({{\rm{L}}}\right)}$$

where *E* is the energy, *A* is the voxel radioactivity map (Bq = s^−1^), *D*_*voxel*_ is the voxel dose (Gy = J/kg), *λ* is the ^166^Ho decay constant of 7.18 × 10^−6 ^s^−1^ [17], and *ρ* is the assumed tissue density of 1.06 kg/L based on liver tissue according to Report 44 of the International Commission on Radiation Units and Measurements [41].

### Evaluation in phantoms and canine patients

Quantification efficacy was evaluated in tissue phantoms and canine patients after standardized injection of predetermined and measured ^166^Ho-MS amounts, including potential influences of (i) the target tissue/radiodensity; (ii) the amounts injected; (iii) CT acquisition parameters; and (iv) the size of the quantification VOI.

#### ^166^Ho-MS suspension preparation

Non-radioactive Ho-MS were prepared, and neutron irradiated to obtain ^166^Ho-MS as previously described (QuiremSpheres™) [35, 44]. These were suspended in sterile water containing 0.1% poloxamer 188 (Pluronic F-68) and 116 mM phosphate buffer by gentle agitation and repeatedly drawing up and down in a syringe. Radioactivity in used materials was measured before and after injection using a dose calibrator (VDC-404 and VDC-606, Comecer, Joure, The Netherlands), corrected for radioactive decay until the moment of CT scanning, and subtracted to calculate the injected radioactivity and corresponding Ho-MS amounts.

#### Injections in quantification phantoms

**Fig. 3:**
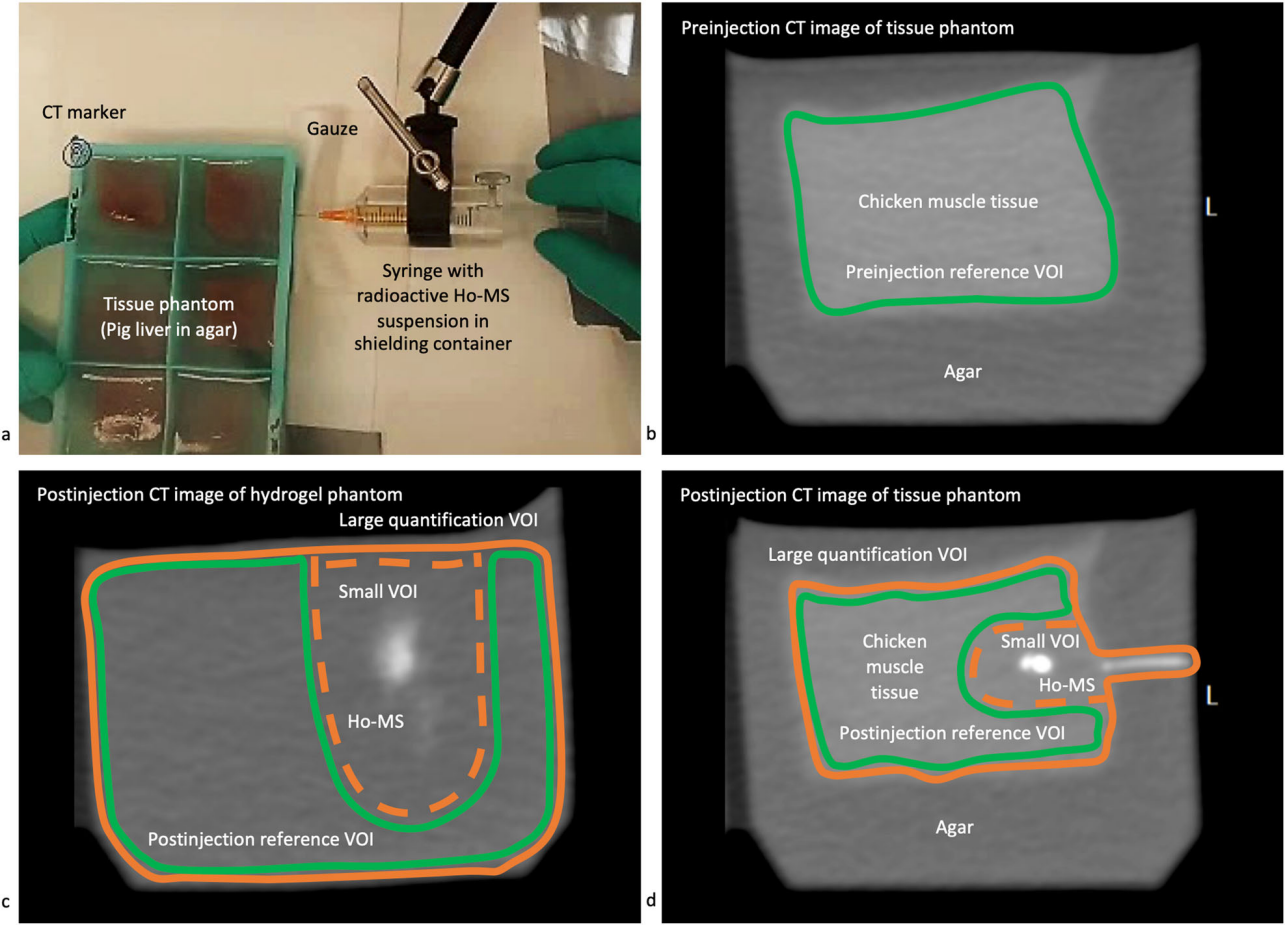
^166^Ho-MS injections and VOI contouring in phantoms to evaluate quantification efficacy on CT images. **a** Injection in liver tissue sample embedded in agar. **b** Preinjection CT image with a preinjection reference VOI contoured around the edges of a chicken muscle tissue sample (green line). **c** Postinjection CT image of a hydrogel sample showing the large quantification VOI contoured around the mold edges (orange line), the small quantification VOI narrowed around visible Ho-MS (dashed orange line), and a postinjection reference VOI without visible Ho-MS (green line). **d** Postinjection CT image showing the same VOI in a chicken muscle tissue sample. CT, Computed tomography; Ho-MS, Holmium microspheres; VOI Volume of interest

Five phantoms were created with six equivalent samples in silicone molds measuring 5 × 5 × 4 cm^3^ (length x width x height), including one spare sample (Supplementary Table S1 and Fig. 3). Phantom 1 contained hydrogel mimicking the cutting response and complex deformation of the human brain during brain shift [45]. Polyvinyl Alcohol 1.125 weight% (Product 363065-1KG; Sigma-Aldrich) and Phytagel 0.425 weight% (Sigma-Aldrich) were dissolved separately in deionized water by continuously stirring for 1 h at 90 °C. The two solutions were mixed in a 1:1 weight ratio at 70 °C under constant stirring for 30 min while covered with aluminum foil. The mixture was poured into molds placed in ice to cool slowly, and subsequently placed in a freezer at -25 °C for 18 h. The phantom was thawed for 24 h prior to the experiment. Phantoms 2 and 3 contained *ex vivo* chicken muscle and pig liver tissue, respectively, measuring ~ 3 × 3 × 3 cm^3^, embedded in agar to prevent Ho-MS leakage out of the samples. Two additional phantoms 4 and 5 were created by replicating phantoms 1 and 3, respectively, for repeated injection of double the Ho-MS amounts.

Each prepared syringe was placed horizontally in a 10-mm thick polymethylmethacrylate syringe container fixed to a laboratory stand (Fig. 3). A 25 G x 40 mm needle (Sterican, B. Braun, Melsungen, Germany) was attached, the syringe was rotated for at least 10 s to get a homogeneous suspension, and ~ 0.2 mL was used to flush the system. The needle was inserted ~ 2.5 cm through the mold, and 0.3 mL was injected. The needle was withdrawn, the outside of the mold was wiped with gauze, and radioactivity in the materials was measured. This was repeated for all samples using one syringe per phantom.

#### Injections in canine patients

Seven canine patients were previously treated by CT-guided ^166^Ho microbrachytherapy at the Academic Veterinary Hospital: Three meningiomas (2.0 cm^3^, 3.7 cm^3^, and 4.1 cm^3^), one glioma (5.0 cm^3^), an anal sac carcinoma (175.4 cm^3^), a soft-tissue sarcoma (36.7 cm^3^), and an oral squamous cell carcinoma (3.4 cm^3^). Tumor volumes were calculated using the largest diameters measured on pretreatment CT images (Eq. 7).

CT-guided injections of 0.1–0.2 mL were performed using standard 22 G needles (Sterican) in extracranial tumors, and a curved needle (Parker Curved Needle Set, Cook Medical, IN, USA) guided through a dedicated cannula [46] in intracranial tumors.

7 $${{\rm{Volume}}}=\frac{\pi }{6}\times {{\rm{length}}}\times {{\rm{width}}}\times {{\rm{height}}}$$

#### Ho-MS amounts

We aimed to inject 7.5 mg Ho-MS in each sample of phantoms 1 to 3 and double the amount (15 mg) in replicated phantoms 4 and 5. In patients, we aimed to inject 5.0 mg Ho-MS per cm^3^ tumor, resulting in varying amounts for different tumor volumes. Injection efficacy (injected *versus* aim) and theoretical Ho-MS concentrations (injected/quantification VOI) were calculated for comparison.

#### CT acquisition

The phantom experiments and patient treatments were performed at the Academic Veterinary Hospital housing the Siemens scanner using 80 and 120 kVp, 400 mAs, 1 mm slice thickness, and a H41s soft-tissue reconstruction kernel (Table 1).

#### Volumes of interest

Reference VOIs were created on both the preinjection and postinjection images to rule out significant radiodensity differences between scans, which were found in phantoms 2, 3, and 5 and in patients 1 and 7 (Supplementary Tables S1 and S6). Preinjection reference VOIs were manually contoured in phantoms around the mold or tissue edges and in patients around the tumor edges on the contrast images, followed by rigid registration on the non-contrast images (Supplementary Table S1 and Fig. 3). In phantoms, these VOIs were copied, and visible Ho-MS were excluded from the contour to create postinjection reference VOIs. In patients, the tumors were mostly filled with Ho-MS, and postinjection reference VOIs were created in soft tissue nearby instead, and only if differences (mean HU > 3) were found in at least three random ellipsoid regions in that tissue. Quantification VOIs were created by copying the preinjection reference VOI on the postinjection images while including all Ho-MS. In phantoms, this was defined as the large VOI, for which results were compared with a small VOI narrowed around visible Ho-MS (Fig. 3). Visible Ho-MS outside the contours were also included, for example, in the injection canal, whereas in patients, hyperdense structures that could be falsely classified as Ho (such as bone and calcifications) were excluded.

#### Outcomes

Quantification results were analyzed using two main outcomes: (i) the Ho recovery, *i.e*., the amount quantified/injected (%), which was deemed sufficient for further analyses between 70 and 130%; and (ii) The Ho volume fraction, *i.e*., the volume of voxels contributing to Ho recovery/quantification VOI (%), to validate results with respect to the used method: all voxels should inherently contribute to subtraction results, whereas only a fraction should contribute to threshold results.

The results were compared: (i) between tissues (phantom types/patients); (ii) between amounts injected/concentrations; (iii) in phantoms between 80 and 120 kVp; and (iv) in phantoms between the large and small VOI. Finally, dose distributions were also visualized in patients to demonstrate clinical proof of principle.

#### Statistical analysis

Numerical data are presented as the mean ± SD if normally distributed and as the median and interquartile range if skewed based on the Shapiro-Wilk test. Significance was tested using independent and paired samples *t*-tests if normally distributed and a Wilcoxon signed rank and Mann–Whitney *U*-test if not normally distributed, respectively. Statistical analysis was performed using IBM SPSS Statistics version 27 with a significance level set at *p* < 0.05.

## Results

### CT calibration

**Fig. 4:**
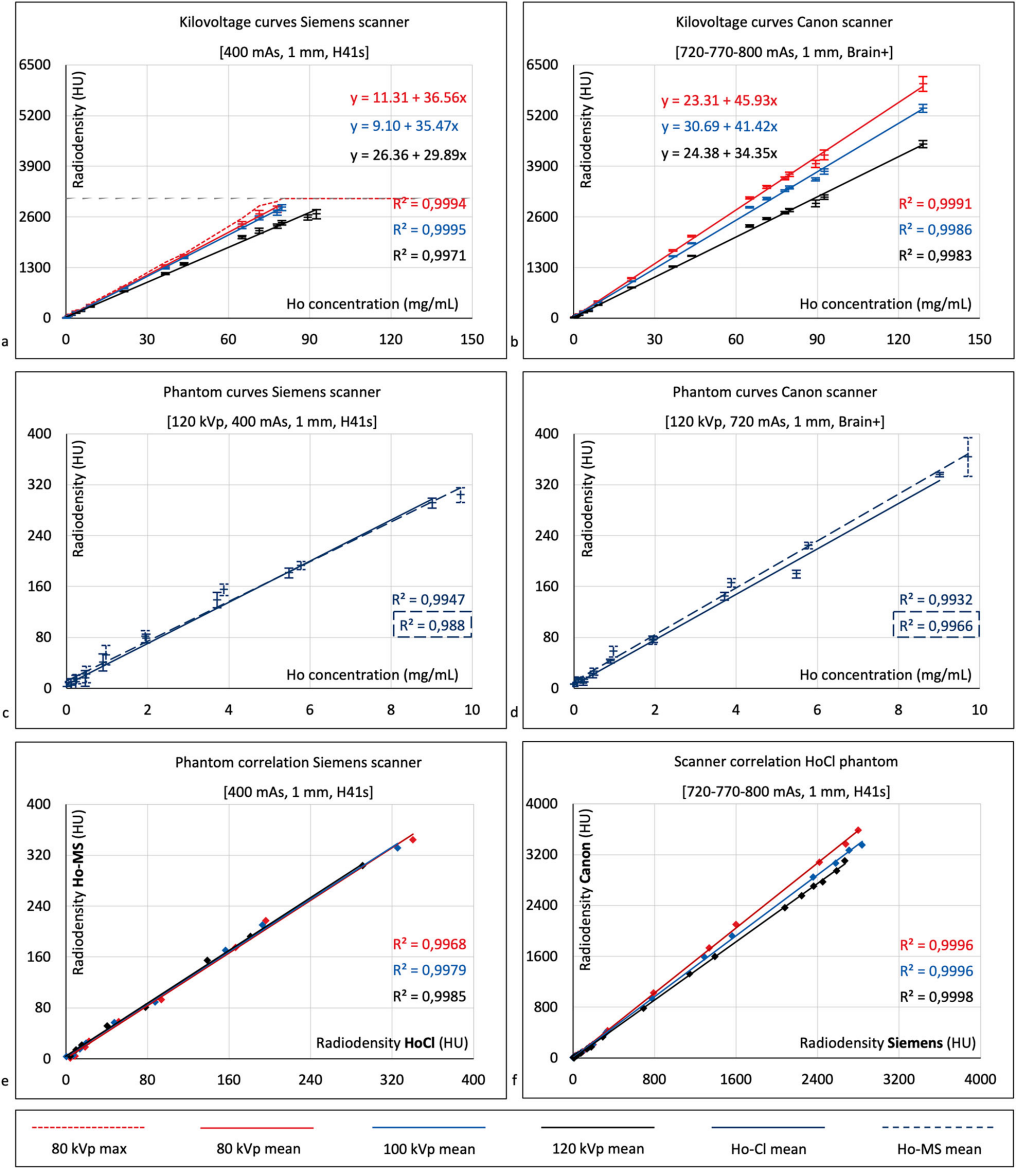
CT calibration curves showing measured HU values (mean ± SD) for different Ho concentrations in a HoCl and a Ho-MS phantom scanned with a Siemens SOMATOM Definition AS and a Canon Aquilion ONE scanner. **a**, **b** HoCl phantom scanned with 80, 100, and 120 kVp. **c**, **d** HoCl *versus* Ho-MS curves for each scanner. **e** Correlation between the HoCl and Ho-MS curves on the Siemens scanner for 80, 100, and 120 kVp. **f** Correlation between the Siemens and the Canon scanner for the HoCl phantom scanned with 80, 100, and 120 kVp. CT, Computed tomography; HoCl, Holmium chloride; Ho-MS, Holmium microspheres; SD, Standard deviation

In all datasets, there was a near-perfect correlation between radiodensity and Ho concentration (*R*^2^ > 0.98, *p* < 0.001), and the regression lines were interchangeable for the Ho-MS and the HoCl phantoms on both scanners (*n* = 9, *R*^2^ = 0.99; Fig. 4 and Supplementary Table S5).

For the Siemens scanner and used quantification parameters (120 kVp, 400 mAs, 1 mm, H41s), the fitted regression model is shown (Eq. 8) which was statistically significant (*R*^2^ = 0.99, *F*(1,16) = 4308.05, *p* < 0.001) with an intercept of 26.36 HU and an increase of 29.89 HU per mg/mL Ho. The Canon scanner had steeper curves for all datasets, with an intercept of 24.38 HU and an increase of 34.35 HU per mg/mL Ho for the matched parameters (120 kVp, 720 mAs, 1 mm, Brain + ; Fig. 4).

Steeper curves were observed for 80 kVp compared to 100 and 120 kVp (Fig. 4 and Supplementary Table S5), whereas differences were marginal for the tube current, slice thickness, and reconstruction kernel. The Siemens scanner’s detection limit was reached for concentrations between 43.6 and 92.5 mg/mL, with markedly lower maxima for bone kernel reconstructions compared to soft tissue.

Standard errors were larger than the intercept for the Siemens scanner using bone kernel reconstructions, and for the Canon scanner using 80 kVp, and bone kernel reconstructions of 100 and 120 kVp, indicating that these regression lines may be forced through zero by eliminating the intercept coefficient:

8 $$y=26.36+29.89x$$

where *y* is Ho (HU_Ho_) and *x* is Ho (mg/mL) (Eq. 3).

### Phantoms

#### Injections

Between 2–12 mg Ho-MS was injected in samples of phantoms 1 to 3, and 7–18 mg in replicated phantoms 4 and 5, with an efficacy between 25–164% (Supplementary Table S1). Injected amounts and concentrations were significantly higher in phantom 4 compared to 1 as planned (12.81 ± 4.05 *versus* 4.82 ± 2.74 mg, *t*(8) = -3.65, *p* = 0.006; 0.2 ± 0.1 *versus* 0.1 ± 0.1 mg/mL; *t*(8) = -3.80, *p* = 0.005), but not in phantom 5 compared to 3 (Supplementary Table S1 and S6).

#### Ho recovery

**Fig. 5:**
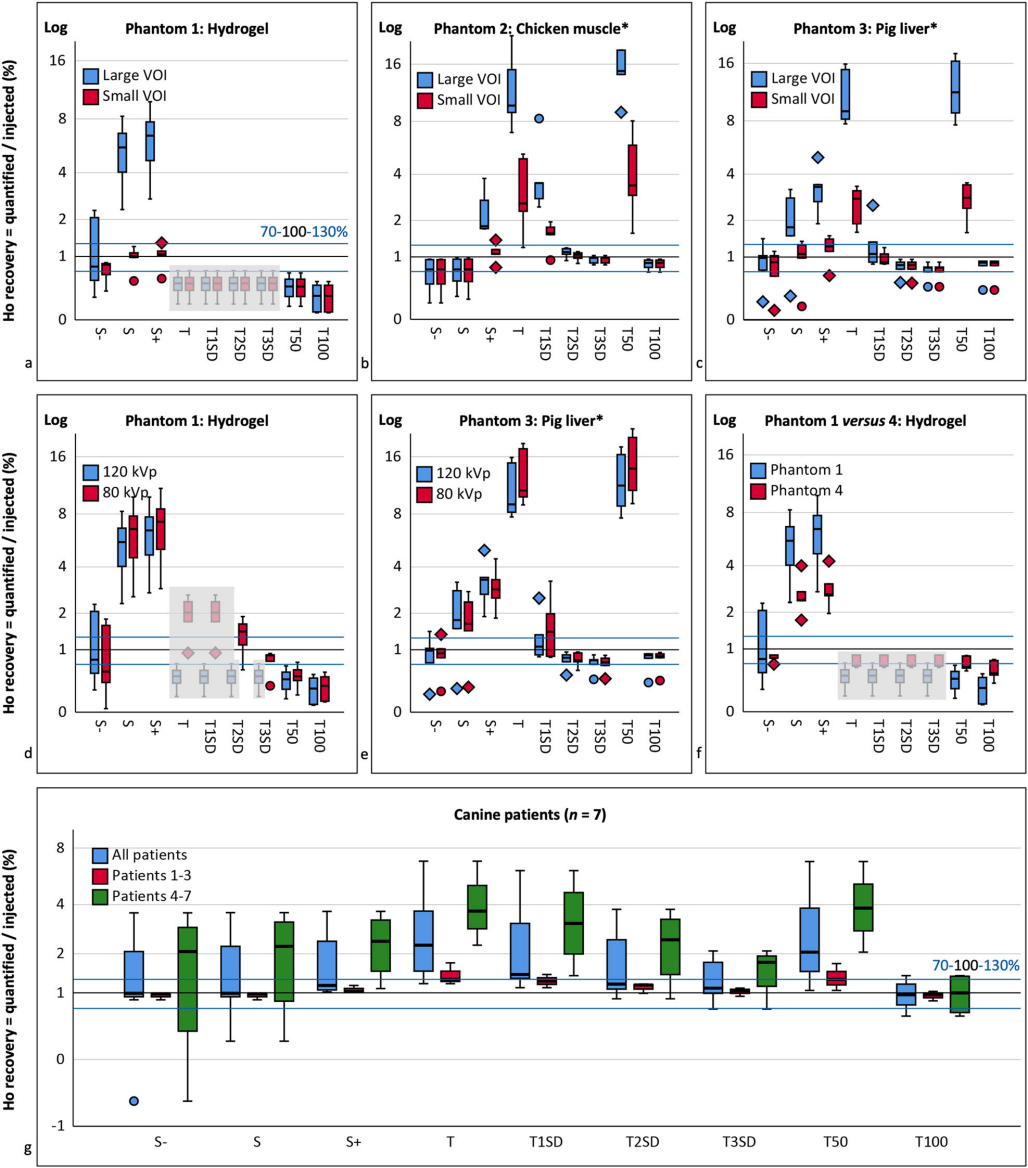
Ho recovery on CT images after standardized injections in replicated phantoms (*n* = 5) and canine patients (*n* = 7). Horizontal lines are shown for the desired 100% recovery (black line) and for sufficient 70–130% (blue lines) recoveries that were further analyzed. Recoveries were extremely under- or overvalued depending on the quantification method used. Phantom results were excluded if classified Ho HU values were lower than the applied CT calibration intercept (gray planes). *HU values of the postinjection reference VOI (without Ho-MS) were used for quantification instead of the preinjection reference VOI because they differed significantly. **a**–**c** Recovery in small *versus* large VOIs in phantoms 1, 2, and 3, respectively. **d**, **e** Recovery for 120 *versus* 80 kVp in phantoms 1 and 3, respectively. **f** Recovery in phantom 1 with significantly more Ho-MS injected compared to phantom 4. **g** Recovery in all canine patients (*n* = 7), and in grouped patients 1, 2, and 3 with significantly more Ho-MS injected compared to grouped patients 4–7. CT, Computed tomography; Ho-MS, Holmium microspheres, S, Subtraction; T, Thresholding; VOI, Volume of interest

The mean Ho recovery ranged from 25–2,042% for all conditions (Table 2 and Fig. 5; Supplementary Table S5), showing large under- and overvalued recoveries with respect to the desired 100%. Some tissue-specific thresholds in the hydrogel phantoms led to HU_Ho_ values lower than the applied calibration intercept and were excluded.

**Table 2:**
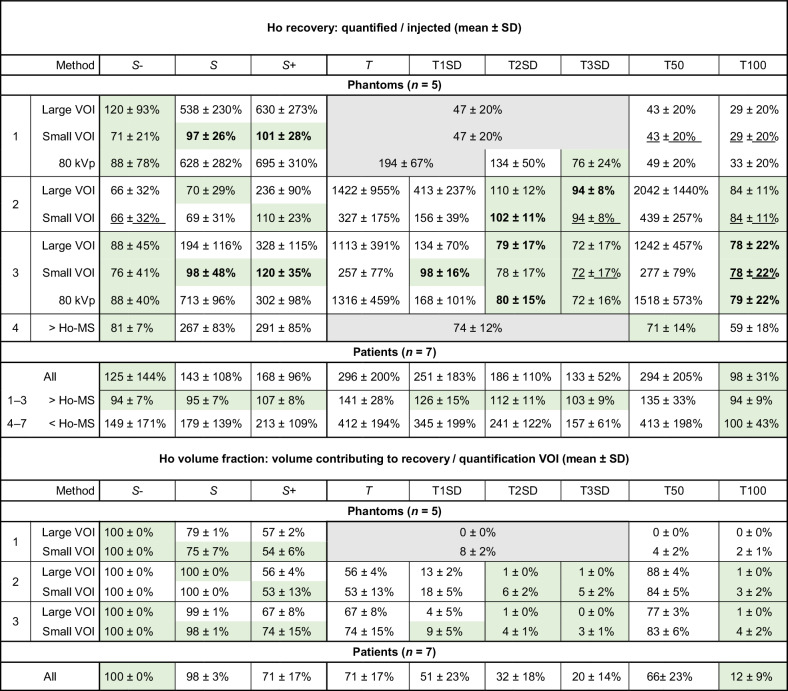
Ho recovery and volume fractions on CT images

Recovery of 100% means that 100% of injected ^166^Ho-MS were quantified. In phantoms, five repeated injections were performed. Recoveries between 70% and 130% were deemed sufficient (green cells) for further testing. Some results were significantly different between methods (bold), were equal between the small and large quantification VOI (underscored), or were discarded if the quantified Ho HU value was lower than the calibration intercept (gray cells). ^*166*^*Ho-MS* Holmium-166 microspheres, *S* subtraction excluding voxels below the baseline tissue radiodensity, *S* *+* subtraction excluding voxels below zero, *S* − subtraction including negative voxels, *SD* Standard deviation, *T* Threshold, *VOI* Volume of interest

For the large VOI in phantom 1, sufficient mean recovery (70–130%) was only found using S −, albeit with a relatively high SD (120 ± 93%). In phantom 2, sufficient recoveries were found using S, T2SD, T3SD, and T100, with T3SD closest to 100% (94 ± 8%). In phantom 3, sufficient recoveries were found using S − and the same sufficient thresholds but with T2SD and T100 closest to 100% (79 ± 17%, 78 ± 22%).

For the small VOI in phantom 1, sufficient recoveries were found also for S and S +, which were closest to 100% (97 ± 26%, 101 ± 28%). In phantom 2, for S +, and for T2SD, T3SD and T100 are equal to the large VOI, with T2SD closest to 100% (102 ± 11%). In phantom 3, for S and S +, and for T2SD, T3SD, and T100 with the addition of T1SD, which was closest to 100% (98 ± 16%). In both tissue phantoms 2 and 3, sufficient recoveries were equal between the large and small VOI for T3SD and T100. For subtraction, insufficient mean recoveries in the large VOI became sufficient in the small VOI showing significant differences for S in phantom 1 and S + in all three phantoms (Table 2 and Supplementary Table S5).

In phantom 1 using 80 kVp, sufficient recoveries were found also using S − which was closest to 100% (88 ± 78%) with the addition of T3SD. In Phantom 3, sufficient recoveries were found for the same methods as 120 kVp with marginal non-significant differences.

In phantom 4 with significantly more Ho-MS injected, sufficient recoveries were found also using S − which was closest to 100% (81 ± 7%) and using T50, while recovery of all included methods improved.

#### Ho volume fractions

**Fig. 6:**
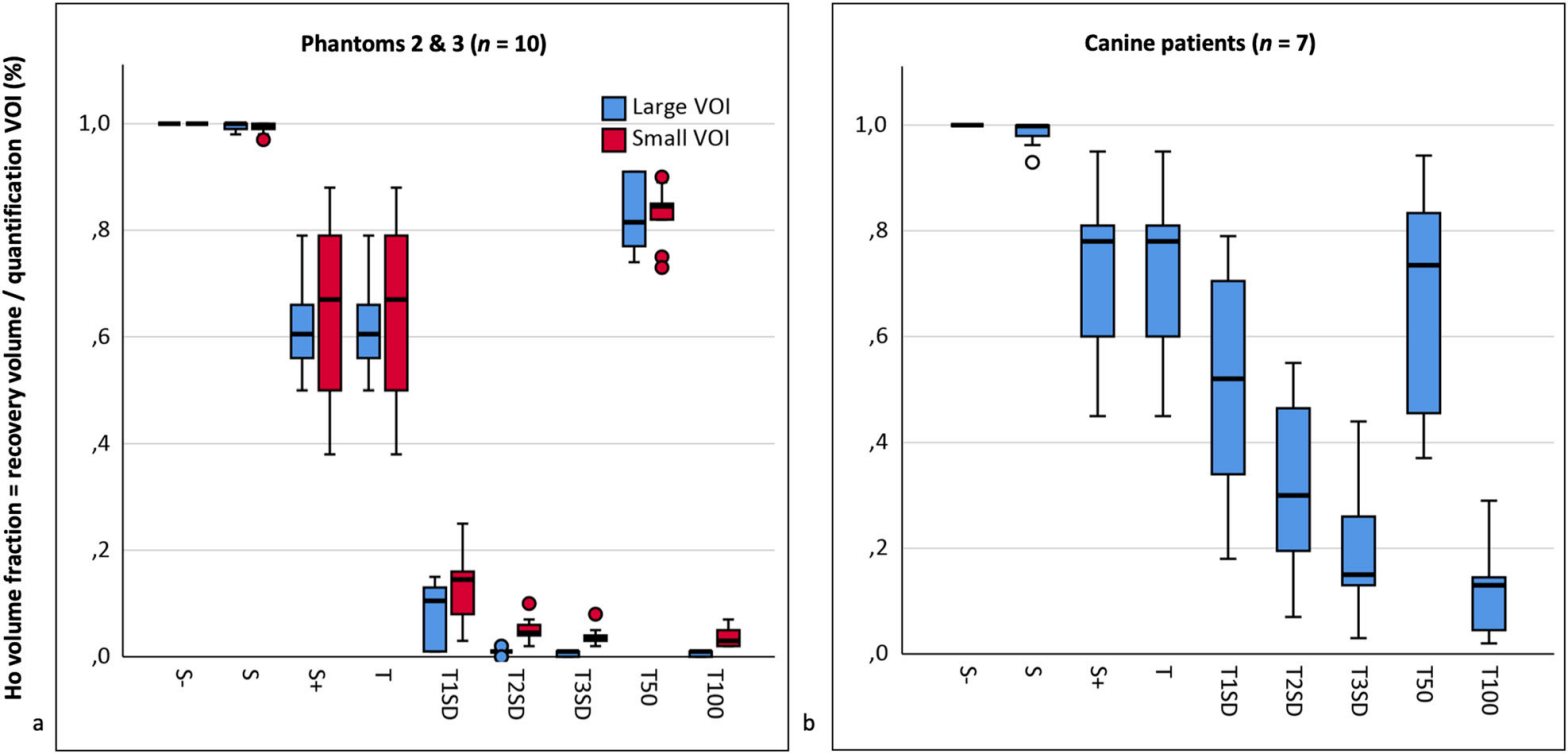
Ho volume fractions on CT after standardized injections in replicated phantoms (*n* = 5) and canine patients (*n* = 7). A fraction of 1 means that 100% of voxels in the contoured VOI contributed to Ho recovery. **a** Merged volume fractions for tissue phantoms 2 and 3. **b** Volume fractions for canine patients. CT, Computed tomography; S, Subtraction; T, Thresholding, VOI, Volume of interest

The mean Ho volume fraction in phantoms ranged from 0–100% (Table 2 and Fig. 6). The fraction was 100 ± 0% using S −, which decreased using S and S +. The maximum fraction using a threshold was 88 ± 4% (T50, phantom 2), which decreased down to 0 ± 0% using higher thresholds (T100, phantoms 1).

For subtraction, the volume fractions were comparable between the large and small VOI, whereas for thresholding, fractions were significantly higher in the small VOI for higher thresholds (T1SD and up, Supplementary Table S6). Higher injection amounts in phantom 4 compared to 1 did not lead to significantly different volume fractions.

### Canine patients

#### Injections

Between 8–256 mg Ho-MS was injected in canine patients with an efficacy between 37–350% (Supplementary Tables S2 and S6). Grouped patients 1–3 had a significantly higher theoretical Ho-MS concentration compared to patients 4–7 (7.4 ± 3.7 *versus* 1.7 ± 3.0 mg/mL, *t*(5) = 3.114, *p* = 0.026).

#### Ho recovery

The mean Ho recovery ranged from 98 to 296% (Table 2, Fig. 5, Supplementary Table S6) with sufficient recoveries for S − (125 ± 144%) and T100 (98 ± 31%), which was closest to 100% with markedly lower variation. Recovery improved markedly in grouped patients 1–3 compared to 4–7, also showing much lower variation.

#### Ho volume fractions

The mean Ho volume fraction ranged from 12–100% (Table 2 and Fig. 6). The fraction was 100 ± 0% using S −, 98 ± 3% using S, and 71 ± 17% using S +. The maximum fraction using a threshold was 71 ± 17% (T) which decreased to 12 ± 9% (T100).

#### Dose estimations and volume histograms

**Fig. 7:**
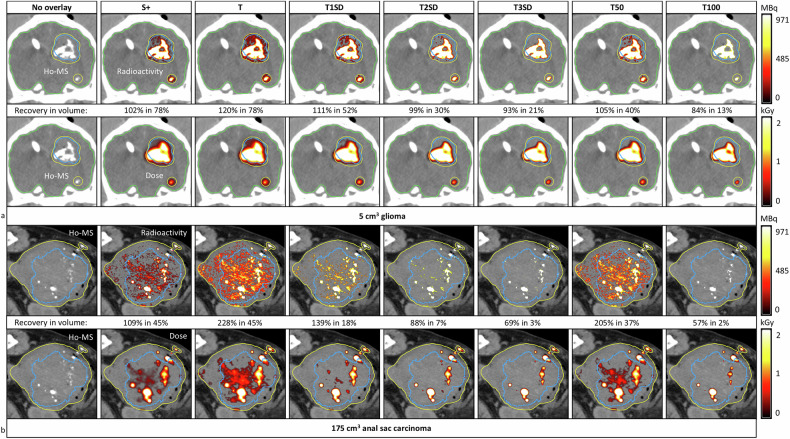
Ho radiation-absorbed dose estimations on CT for different quantification methods in two client-owned dogs after previous ^166^Ho microbrachytherapy. Radioactivity (MBq) and dose-overlays (kGy) are visualized and quantified as Ho recovery (quantified/injected (%)) in volume (volume contributing to Ho recovery/quantification VOI (%)). Subtraction results, including negative values, were also evaluated in our quantification experiments, but not in these dosimetry results as negative doses are not displayed in the used software. Orientation: left = left; right = right; top = dorsal; bottom = ventral. **a** Results in a French Bulldog with a 5.0 cm^3^ glioma showing the radioactivity per voxel (top row) and the cumulative doses (bottom row) with respect to the tumor (blue line), the quantification VOI (yellow line) and the whole brain (green line). **b** Results for a crossbreed labrador retriever with a 175 cm^3^ anal sac carcinoma in the tumor (blue line) and the quantification VOI (yellow line). S +, Subtraction excluding voxels below zero; SD, Standard deviation; T*,* Threshold; VOI, Volume of interest

**Fig. 8:**
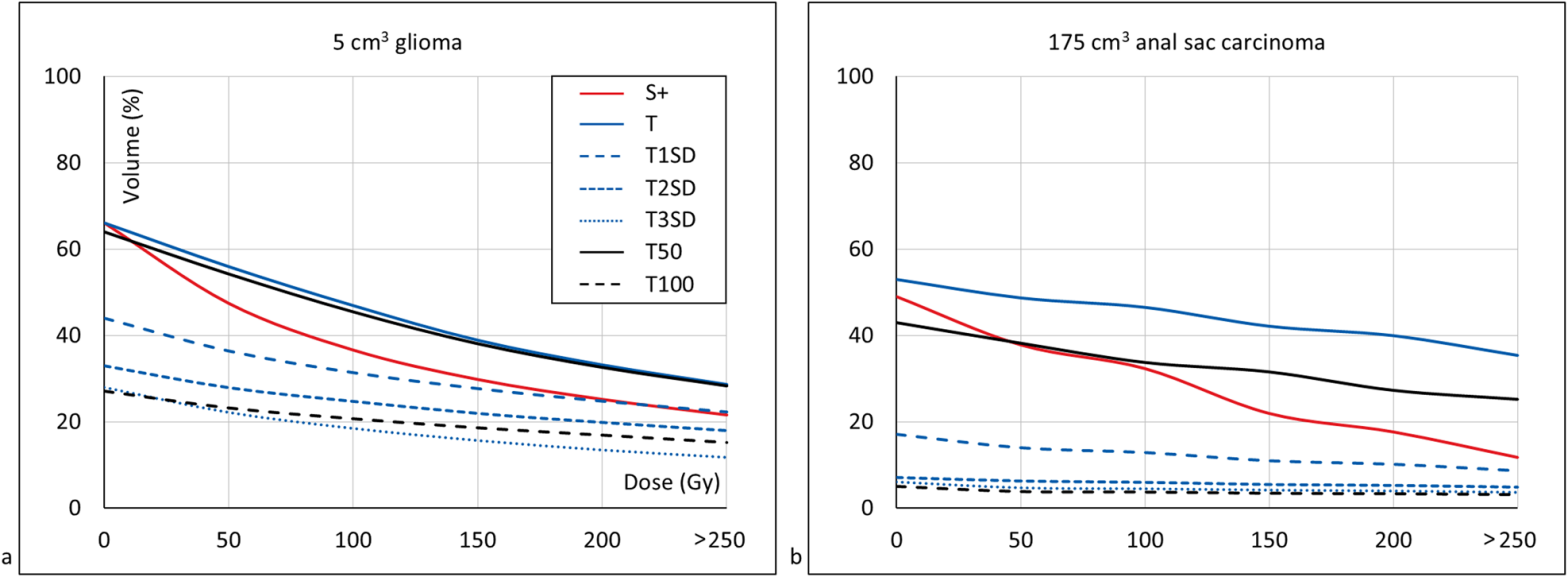
Dose-volume histograms corresponding to displayed Ho radiation-absorbed dose estimations on CT images of two canine patients for different quantification methods. Subtraction results, including negative values, were also evaluated in our quantification experiments, but not in these dosimetry results, as negative doses are not displayed in the used software. **a** Dose-volume histograms of a 5.0 cm^3^ glioma in a French Bulldog corresponding to Fig. 7a. **b** Dose-volume histograms of a 175 cm^3^ anal sac carcinoma in a crossbreed labrador retriever corresponding to Fig. 7b. CT, Computed tomography; S, Subtraction; SD, Standard deviation; T, Thresholding

Radiation-absorbed doses were successfully modeled (Fig. 7), and corresponding dose-volume histograms were created (Fig. 8), which also showed a decrease in dose coverage for higher thresholds and higher volume fractions for subtraction and lower thresholds.

## Discussion

In this study, we set out to find a relatively easy, robust, and accurate method of quantifying Ho-MS and to generate corresponding doses on CT for clinical application. CT calibrations showed near-perfect linearity between radiodensity and Ho concentration for multiple conditions, however, lines were steeper for the Canon scanner with a larger HU range compared to the Siemens scanner. Calibration parameters were, therefore, not interchangeable between scanners, which is desired for clinical implementation and could be true for scanners with equal HU ranges. The latter was not evaluated, and parameters should be determined per scanner.

Steeper regression lines were also found for lower tube voltages, indicating higher sensitivity with desired lower radiation exposure. However, quantification in phantoms showed no evident differences while the Siemens scanner’s detection limit was reached at lower concentrations using 80 kVp. Patients were therefore scanned using 120 kVp and we advise checking for such technical limitations prior to clinical application, which can be overcome by lowering injection amounts, increasing tube voltages, and examining postinjection voxel values.

Finally, different exposures and reconstruction kernels also showed no evident differences in regression lines, except for lower detection limits for bone kernel reconstructions compared to soft tissue. Quantification results were not compared for different kernels, but this is advised when non-soft-tissue kernels are required.

CT calibration was limited by two factors. Firstly, CT measurements are not generally reproducible between scanners or scans because of technical differences [47, 48], which impedes the translation of calibration parameters and may negatively affect quantification. Secondly, the phantoms were scanned in free air instead of using an *in vivo* mimicking phantom [48]. An application-specific phantom should be developed using tailored material composition and thickness [30], and Ho concentrations, although more knowledge is required on CT voxel concentrations of Ho, and the quantification object or potential radiodensity differences should not negatively influence the calibration.

Sufficient mean Ho recoveries (70–130%) were mostly found using S − (albeit with relatively high SD) and T2SD, T3SD, and T100, with significant improvements for higher *in vivo* Ho amounts/concentrations, and for the small VOI using subtraction.

Subtraction using a single HU_reference_ value rendered sufficient mean recovery but with high variation (93% for subtraction *versus* 24% for thresholding) and results were strongly dependent on the VOI, which is undesired for clinical application. This could be improved by implementing voxel-based registration of pre- and postinjection scans, but this might be challenging with submillimeter CT resolutions while scans may still vary in baseline radiodensity.

Thresholding showed sufficient means with relatively low variation, independence of the VOI, and low volume fractions. However, results were insufficient and undervalued in hydrogel phantoms with relatively low baseline radiodensity and higher Ho-MS spread. Thresholding performed markedly better in tissue phantoms with higher radiodensity more representative of the clinical situation. Fixed threshold T100 results were comparable to previous study results of ≥ 76% recovery [29], and were surpassed mostly using tissue-specific T2SD (5 ×) but also T1SD (1 ×) and T3SD (2 ×) depending on the phantom/patient and the VOI.

CT quantification efficacy was clinically tested using images from a limited number of patients (*n* = 7) and tumor types (*n* = 5) that had relatively soft consistency. As observed, quantification results were underestimated in tissues with very low baseline radiodensity and improved for higher concentrations. This should be taken into consideration by ensuring high local amounts and validating quantification results with respect to the baseline radiodensity/tumor consistency for more patients and different tumor types in the future.

We looked at mean radiodensity and quantification results and deemed 70–130% recovery sufficient for further analysis, as quantitative intra-operative feedback is currently lacking. However, little is known about (varying) local voxel concentrations and with that, corresponding minimum and maximum Ho detectability, especially taking varying CT image noise into account. In addition, we had a limited number of samples per condition. Next research should analyze Ho-MS concentrations and local distributions at individual voxel levels for more samples, using advanced acquisition and postprocessing techniques, such as iterative reconstructions and dual-energy CT, which facilitates image-based material decomposition to detect and quantify Ho-MS [30].

At last, ^166^Ho radiation-absorbed dose estimations were successfully implemented as a proof of principle, and dose-volume histograms were in line with quantification results. Clinical implementation for therapeutic safety and efficacy assessment should follow for extended evaluation.

To conclude, CT radiodensity increased linearly with Ho concentration for multiple settings on both scanners, but calibration parameters were not interchangeable between them because of technical differences. Thresholding showed better quantification results with less dependency on the VOI and reliable spatial recovery compared to subtraction using a single reference value. Fixed threshold T100 performed best in patients (98 ± 31%) and T2SD in phantoms (102 ± 11%). These methods should be considered for further clinical application in combination with radioactive measurements and intra-operative Ho-MS and dose visualizations for definitive treatment evaluation.

## Supplementary information

**Additional file 1:** **Supplementary Table S1.** ^166^Ho-MS injection and quantification parameters in veterinary patients. **Supplementary Table S2.** ^166^Ho-MS injection and quantification parameters in phantoms (*n* = 5). **Supplementary Table S3.** Planned and measured holmium (Ho) concentrations in a Ho chloride (Ho-Cl) phantom and a Ho poly(L-lactic) acid microspheres (Ho-MS) phantom with corresponding CT calibration measurement results consisting of the maximum (max.) and mean Hounsfield Unit (HU) values with the standard deviation (SD) for two CT scanners and one combination of acquisition parameters. The exposures (mAs) between the two scanners were set based on the matched CTDIvol. The Ho concentrations in the Ho-Cl phantom were determined using Inductively Coupled Plasma Optical Emission Spectrometry (ICP-OES) and in the Ho-MS phantom by weighing the necessary amounts of Ho-MS upon preparation. The measured HU values on CT were used to plot CT calibration curves and to calculate the corresponding intercept and slope values with regression statistics (Fig. 5). *Maximum HU of scanner reached (3071 HU for Siemens scanner). NA = Not applicable. **Supplementary Table 4.** CT acquisition parameters used for CT calibration for holmium using two different scanners (Siemens SOMATOM Definition AS and Canon Aquilion ONE). Exposures (mAs) were set on the Siemens scanner and the resulting CTDIvol (mGy) values per setting were used on the Canon scanner to obtain equal exposures with respect to the scanned volumes, for proper comparison of measured HU values. **Supplementary Table 5.** CT calibration results for detection of holmium (Ho) based on Hounsfield Unit (HU) measurements in a Ho (III) chloride hexahydrate (Ho-Cl_3_) phantom and a Ho poly(L-lactic) acid microspheres (Ho-PLLA-MS) phantom both containing multiple Ho concentrations (n). CT scans were acquired using a Siemens SOMATOM Definition AS (top) and a Canon Aquilion One (bottom) scanner. Each row represents one dataset acquired by a combination of different acquisition parameters: tube kilovoltage peak (kVp), exposure (mAs)/CTDIvol (mGy), slice thickness (mm), reconstructed using two soft-tissue kernels, and one bone kernel. The Ho concentrations in the Ho-Cl_3_ phantom reached the maximum HU value of the Siemens scanner (HU of 3,071), and we included the maximum (Max.) Ho concentration (mg/mL) that could be detected before this max. HU value was reached, together with the number of concentrations/measurement points (*n*) that were included to calculate the calibration intercept (*m*) and slope (*b*) values with their standard error (SE). Statistics of the regression are also included: *R*-squared (*R*^2^) values, the *F*-statistic (F) of the ANOVA test with the degrees of freedom (df), and *p*-values of the coefficients. **Supplementary Table 6.** Descriptive statistics and test results for holmium-166 microspheres injections, quantification, recovery, and volume fractions after injection in phantoms and veterinary patients.

## Data Availability

The datasets used and/or analyzed during the current study are available from the corresponding author upon reasonable request.

## Abbreviations

^166^Ho-MS: Holmium-166 microspheres
CT: Computed tomography
DPK: Dose point kernel
HoCl: Holmium chloride
MRI: Magnetic resonance imaging
S: Subtraction
SD: Standard deviation
T: Threshold
VOI: Volume of interest
