# Supplementary material for: Quantitative CT imaging and radiation-absorbed dose estimations of 166Ho microspheres: paving the way for clinical application
Source: Eur Radiol Exp. 2024 Oct 14;8:116. doi: 10.1186/s41747-024-00511-8 (PMC11473764; doi:10.1186/s41747-024-00511-8)
Supplement: Supplementary file 1 — Additional file 1: Supplementary Table S1. 166Ho-MS injection and quantification parameters in veterinary patients. Supplementary Table S2. 166Ho-MS injection and quantification parameters in phantoms (n = 5). Supplementary Table S3. Planned and measured holmium (Ho) concentrations in a Ho chloride (Ho-Cl) phantom and a Ho poly(L-lactic) acid microspheres (Ho-MS) phantom with corresponding CT calibration measurement results consisting of the maximum (max.) and mean Hounsfield Unit (HU) values with the standard deviation (SD) for two CT scanners and one combination of acquisition parameters. The exposures (mAs) between the two scanners were set based on the matched CTDIvol. The Ho concentrations in the Ho-Cl phantom were determined using Inductively Coupled Plasma Optical Emission Spectrometry (ICP-OES) and in the Ho-MS phantom by weighing the necessary amounts of Ho-MS upon preparation. The measured HU values on CT were used to plot CT calibration curves and to calculate the corresponding intercept and slope values with regression statistics (Fig. 5). *Maximum HU of scanner reached (3071 HU for Siemens scanner). NA = Not applicable. Supplementary Table 4. CT acquisition parameters used for CT calibration for holmium using two different scanners (Siemens SOMATOM Definition AS and Canon Aquilion ONE). Exposures (mAs) were set on the Siemens scanner and the resulting CTDIvol (mGy) values per setting were used on the Canon scanner to obtain equal exposures with respect to the scanned volumes, for proper comparison of measured HU values. Supplementary Table 5. CT calibration results for detection of holmium (Ho) based on Hounsfield Unit (HU) measurements in a Ho (III) chloride hexahydrate (Ho-Cl3) phantom and a Ho poly(L-lactic) acid microspheres (Ho-PLLA-MS) phantom both containing multiple Ho concentrations (n). CT scans were acquired using a Siemens SOMATOM Definition AS (top) and a Canon Aquilion One (bottom) scanner. Each row represents one dataset acquired by a com [file 41747_2024_511_MOESM1_ESM.docx]

**Quantitative CT imaging and dose estimations of ^166^Ho microspheres: paving the way for clinical application**

**ELECTRONIC SUPPLEMENTARY MATERIAL**

**Supplementary Table S1.** ^166^Ho-MS injection and quantification parameters in veterinary patients.

| Injection and quantification parameters | | | | | | | | |
| --- | --- | --- | --- | --- | --- | --- | --- | --- |
| Patient | Ho-MS injections | | | | | Reference VOI pre | | |
|  | Ho | Radioactivity | Aim | Injected | | Volume | Radiodensity | |
|  | % | MBq/mg | mg | mg | Efficacy | mL | mean HU | ± SD |
| 1 | 19.56 | 1.90 | 28.12 | 36.35 | 129% | 2.20 | 42.84 | ± 16.90 |
| 2 | 19.88 | 3.40 | 25.68 | 17.63 | 69% | 2.64 | 49.35 | ± 15.64 |
| 3 | 19.74 | 5.50 | 16.48 | 57.63 | 350% | 5.26 | 37.13 | ± 9.10 |
| 4 | 20.02 | 2.50 | 21.97 | 8.12 | 37% | 4.55 | 51.89 | ± 9.88 |
| 5 | 19.37 | 11.10 | 211.12 | 255.56 | 121% | 86.17 | 46.93 | ± 11.02 |
| 6 | 19.83 | 6.40 | 153.02 | 73.32 | 48% | 37.88 | 48.29 | ± 11.86 |
| 7 | 19.34 | 4.20 | 19.49 | 23.95 | 123% | 2.58 | 44.25 | ± 12.04 |
|  | | | | | | | | |
| Patient | Reference VOI post | | | Quantification VOI | | | Reference VOI used | Theoretical  Ho-MS concentration quantification VOI |
|  | Volume | Radiodensity | | Volume | Radiodensity | |  |  |
|  | mL | mean HU | ± SD | mL | mean HU | ± SD |  | mg/mL |
| 1 | 2.20 | 48.42 | ± 9.38 | 3.31 | 112.07 | ± 127.77 | post | 10.98 |
| 2 | NA | NA | | 4.89 | 67.73 | ± 56.95 | pre | 3.61 |
| 3 |  |  |  | 7.44 | 81.74 | ± 155.35 | pre | 7.75 |
| 4 |  |  |  | 4.55 | 70.94 | ± 82.32 | pre | 1.78 |
| 5 |  |  |  | 188.74 | 42.71 | ± 60.15 | pre | 1.35 |
| 6 |  |  |  | 66.49 | 71.73 | ± 23.47 | pre | 1.10 |
| 7 | 2.58 | 64.44 | ± 8.00 | 8.80 | 81.74 | ± 88.83 | post | 2.72 |

*Ho-MS* Holmium microspheres *SD* Standard deviation, *NA* Not applicable, *VOI* Volume of interest.

**Supplementary Table S2.** ^166^Ho-MS injection and quantification parameters in phantoms (*n* = 5).

| Phantom | | Ho-MS injections | | | | | | |  |
| --- | --- | --- | --- | --- | --- | --- | --- | --- | --- |
|  |  |  |  |  |  |  |  |  |  |
|  |  | Ho | Radioactivity | Aim | Injected | | | |  |
|  |  | % | MBq/mg | mg | mean mg | ± SD | Efficacy | |  |
| 1 | Hydrogel | 19.56 | 1.10 | 7.50 | 4.82 | ± 2.74 | 64 | ± 37% |  |
| 2 | Chicken muscle |  | 1.09 |  | 5.32 | ± 2.75 | 71 | ± 37% |  |
| 3 | Pig liver |  | 1.08 |  | 8.60 | ± 2.75 | 115 | ± 37% |  |
| 4 | Hydrogel |  | 1.07 | 15.00 | 12.81 | ± 4.05 | 85 | ± 27% |  |
| 5 | Pig liver |  | 1.07 |  | 9.69 | ± 3.16 | 65 | ± 21% |  |
|  | | | | | | | | |  |
| Phantom | Reference VOI pre | | Reference VOI post | | Large quantification VOI | | Small quantification VOI | |  |
|  |  |  |  |  |  |  |  |  |  |
|  | Volume | | Volume | | Volume | | Volume | |  |
|  | mean mL | ± SD | mean mL | ± SD | mean mL | ± SD | mean mL | ± SD |  |
| 1 | 58.79 | ± 0.29 | 55.15 | ± 1.71 | 58.75 | ± 0.72 | 3.60 | ± 1.68 |  |
| 2 | 15.67 | ± 0.99 | 12.66 | ± 1.01 | 15.75 | ± 0.95 | 3.09 | ± 1.21 |  |
| 3 | 19.78 | ± 2.94 | 16.82 | ± 2.51 | 20.03 | ± 2.80 | 3.21 | ± 0.79 |  |
| 4 | 55.66 | ± 0.90 | 41.80 | ± 4.14 | 55.55 | ± 1.11 | 13.75 | ± 4.95 |  |
| 5 | 17.73 | ± 1.92 | 11.72 | ± 2.96 | 18.20 | ± 2.15 | 6.48 | ± 3.28 |  |
|  | | | | | | | | |  |
| Phantom | Reference VOI pre | | Reference VOI post | | Quantification VOI | | Reference VOI used | Theoretical  Ho-MS concentration quantification VOI |  |
|  |  |  |  |  |  |  |  |  |  |
|  | Radiodensity | | Radiodensity | | Radiodensity | |  |  |  |
|  | mean HU | ± SD | mean HU | ± SD | mean HU | ± SD |  | mg/mL |  |
| 1) 120 kVp | 3.74 | ± 5.10 | 3.97 | ± 5.76 | 4.16 | ± 8.43 | pre | 0.08 ± 0.05 |  |
| 1) 80 kVp | 4.02 | ± 7.05 | 4.14 | ± 7.83 | 4.38 | ± 10.40 |  |  |  |
| 2) 120 kVp | 69.48 | ± 7.87 | 57.10 | ± 6.57 | 58.49 | ± 35.99 | post | 0.34 ± 0.18 |  |
| 3) 120 kVp | 58.90 | ± 43.67 | 53.18 | ± 37.48 | 55.46 | ± 56.39 | post | 0.44 ± 0.17 |  |
| 3) 80 kVp | 61.93 | ± 43.90 | 55.16 | ± 37.82 | 57.91 | ± 59.42 |  |  |  |
| 4) 120 kVp | 3.86 | ± 6.12 | 3.93 | ± 7.12 | 4.94 | ± 18.54 | pre | 0.23 ± 0.07 |  |
| 5) 120 kVp | 58.03 | ± 45.46 | 52.22 | ± 37.68 | 53.48 | ± 62.80 | post | 0.53 ± 0.13 |  |

^166^Ho-MS Holmium-166 microspheres, *SD* Standard deviation, *VOI* Volume of interest.

**Supplementary Table S3.** Planned and measured holmium (Ho) concentrations in a Ho chloride (Ho-Cl) phantom and a Ho poly(L-lactic) acid microspheres (Ho-MS) phantom with corresponding CT calibration measurement results consisting of the maximum (max.) and mean Hounsfield Unit (HU) values with the standard deviation (SD) for two CT scanners and one combination of acquisition parameters. The exposures (mAs) between the two scanners were set based on the matched on CTDIvol. The Ho concentrations in the Ho-Cl phantom were determined using Inductively Coupled Plasma Optical Emission Spectrometry (ICP-OES) and in the Ho-MS phantom by weighing the necessary amounts of Ho-MS upon preparation. The measured HU values on CT were used to plot CT calibration curves and to calculate the corresponding intercept and slope values with regression statistics (**Figure 5**). *Maximum HU of scanner reached (3071 HU for Siemens scanner). NA = Not applicable.

| CT calibration | | | | | | | | | |
| --- | --- | --- | --- | --- | --- | --- | --- | --- | --- |
| Ho concentration | | | | HU measurements | | | | | |
| Planned | Measured | | Difference | Siemens SOMATOM Definition AS | | | Canon Aquilion ONE | | |
|  |  |  |  | 120 kVp 400 mAs 1 mm H41s | | | 120 kVp 720 mAs 1 mm Brain+ | | |
| mg/mL | mg/mL | ± SD |  | max. HU | mean HU | ± SD | max. HU | mean HU | ± SD |
| HoCl phantom | | | | | | | | | |
| 0 | 0.00 | ± 0.00 | NA | 26 | -0.79 | ± 9.28 | 17 | 6.04 | ± 3.57 |
| 0.125 | 0.11 | ± 0.00 | -12% | 39 | 4.54 | ± 9.51 | 23 | 13.02 | ± 2.67 |
| 0.25 | 0.23 | ± 0.00 | -8% | 33 | 10.02 | ± 7.17 | 21 | 7.60 | ± 5.01 |
| 0.5 | 0.46 | ± 0.01 | -8% | 51 | 15.51 | ± 11.46 | 29 | 20.08 | ± 2.74 |
| 1 | 0.90 | ± 0.02 | -10% | 77 | 40.70 | ± 11.98 | 52 | 42.82 | ± 3.25 |
| 2 | 1.96 | ± 0.04 | -2% | 98 | 78.47 | ± 4.64 | 87 | 76.49 | ± 3.43 |
| 4 | 3.71 | ± 0.07 | -7% | 169 | 138.80 | ± 10.67 | 155 | 144.73 | ± 3.82 |
| 6 | 5.48 | ± 0.11 | -9% | 201 | 181.33 | ± 8.56 | 194 | 179.56 | ± 4.81 |
| 10 | 9.01 | ± 0.18 | -10% | 313 | 291.24 | ± 6.88 | 349 | 336.03 | ± 4.61 |
| 25 | 21.40 | ± 0.43 | -14% | 721 | 694.52 | ± 12.81 | 823 | 789.99 | ± 8.90 |
| 40 | 36.70 | ± 0.73 | -8% | 1203 | 1145.63 | ± 24.86 | 1391 | 1326.93 | ± 13.57 |
| 50 | 43.60 | ± 0.87 | -13% | 1427 | 1394.48 | ± 28.96 | 1659 | 1601.75 | ± 10.40 |
| 75 | 65.00 | ± 1.30 | -13% | 2180 | 2081.01 | ± 38.51 | 2489 | 2368.03 | ± 28.35 |
| 80 | 71.30 | ± 1.43 | -11% | 2363 | 2245.74 | ± 63.47 | 2643 | 2557.26 | ± 23.33 |
| 85 | 78.00 | ± 1.56 | -8% | 2477 | 2362.18 | ± 54.17 | 2821 | 2709.33 | ± 28.60 |
| 90 | 79.70 | ± 1.59 | -11% | 2601 | 2448.96 | ± 56.30 | 2923 | 2775.18 | ± 39.22 |
| 100 | 89.30 | ± 1.79 | -11% | 2739 | 2584.97 | ± 66.56 | 3101 | 2946.23 | ± 86.10 |
| 95 | 92.50 | ± 1.85 | -3% | 2903 | 2668.95 | ± 117.34 | 3282 | 3108.30 | ± 56.54 |
| 150 | 129.00 | ± 2.58 | -14% | 3071* | 3064.47 | ± 66.65 | 4723 | 4470.00 | ± 91.33 |
| Ho-MS phantom | | | | | | | | | |
| 0 | 0.00 |  | NA | 25 | 1.98 | ± 7.79 | 16 | 6.42 | ± 2.78 |
| 0.125 | 0.12 |  | -4% | 38 | 4.69 | ± 9.13 | 23 | 15.53 | ± 2.68 |
| 0.25 | 0.25 |  | 0% | 79 | 14.40 | ± 6.89 | 52 | 13.82 | ± 3.77 |
| 0.5 | 0.49 |  | -2% | 160 | 21.71 | ± 12.30 | 123 | 26.12 | ± 6.89 |
| 1 | 0.97 |  | -3% | 189 | 52.17 | ± 14.67 | 201 | 57.72 | ± 8.26 |
| 2 | 1.94 |  | -3% | 179 | 80.97 | ± 8.89 | 126 | 75.68 | ± 6.34 |
| 4 | 3.88 |  | -3% | 202 | 154.90 | ± 9.42 | 216 | 165.81 | ± 5.92 |
| 6 | 5.78 |  | -4% | 249 | 193.03 | ± 5.77 | 246 | 224.47 | ± 5.33 |
| 10 | 9.71 |  | -3% | 367 | 303.80 | ± 10.31 | 510 | 363.71 | ± 28.42 |

**Supplementary Table 4.** CT acquisition parameters used for CT calibration for holmium using two different scanners (Siemens SOMATOM Definition AS and Canon Aquilion ONE). Exposures (mAs) were set on the Siemens scanner and the resulting CTDIvol (mGy) values per setting were used on the Canon scanner to obtain equal exposures with respect to the scanned volumes, for proper comparison of measured HU values.

| **Tube kilovoltage peak** | **Exposure** | | | | | **CTDIvol** | | | |
| --- | --- | --- | --- | --- | --- | --- | --- | --- | --- |
| **kVp** | **mAs** | | | | | **mGy** | | | |
|  | Set | Siemens | | Canon | | Siemens | | Canon | |
|  |  | Ho-Cl | Ho-MS | Ho-Cl | Ho-MS | Ho-Cl | Ho-MS | Ho-Cl | Ho-MS |
| 80 | free | 70 | 76 | 150 | 160 | 3.44 | 3.73 | 3.30 | 3.60 |
|  | 200 | 200 | 200 | 440 | 440 | 9.83 | 9.83 | 9.90 | 9.90 |
|  | 400 | 400 | 400 | 800 | 800 | 19.65 | 19.65 | 18.10 | 18.10 |
| 100 | free | 74 | 79 | 140 | 150 | 7.28 | 7.77 | 7.10 | 7.60 |
|  | 200 | 200 | 200 | 390 | 390 | 19.66 | 19.66 | 19.70 | 19.70 |
|  | 400 | 400 | 400 | 770 | 770 | 39.33 | 39.33 | 39.30 | 39.30 |
| 120 | free | 76/77 | 81 | 140 | 150 | 12.33/12.49 | 13.14 | 12.50 | 13.30 |
|  | 200 | 200 | 200 | 360 | 360 | 32.44 | 32.44 | 32.40 | 32.40 |
|  | 400 | 400 | 400 | 720 | 720 | 64.87 | 64.87 | 64.80 | 64.80 |

**Supplementary Table 5.** CT calibration results for detection of holmium (Ho) based on Hounsfield Unit (HU) measurements in a Ho (III) chloride hexahydrate (Ho-Cl_3_) phantom and a Ho poly(L-lactic) acid microspheres (Ho-PLLA-MS) phantom both containing multiple Ho concentrations (n). CT scans were acquired using a Siemens SOMATOM Definition AS (top) and a Canon Aquilion One (bottom) scanner. Each row represents one dataset acquired by a combination of different acquisition parameters: tube kilovoltage peak (kVp), exposure (mAs) / CTDIvol (mGy), slice thickness (mm), reconstructed using two soft tissue kernels and one bone kernel. The Ho concentrations in the Ho-Cl_3_ phantom reached the maximum HU value of the Siemens scanner (HU of 3,071), and we included the maximum (Max.) Ho concentration (mg/mL) that could be detected before this max. HU value was reached, together with the number of concentrations/measurement points (n) that were included to calculate the calibration intercept (m) and slope (b) values with their standard error (SE). Statistics of the regression are also included: R Squared (R^2^) values, the F-statistic (F) of the ANOVA test with the degrees of freedom (df), and p-values of the coefficients.

| CT calibration results | | | | | | | | | | | | | | | | | | | | | | |  |
| --- | --- | --- | --- | --- | --- | --- | --- | --- | --- | --- | --- | --- | --- | --- | --- | --- | --- | --- | --- | --- | --- | --- | --- |
| Siemens SOMATOM Definition AS | | | | | | | | | | | | | | | | | | | | | | |  |
|  |  |  |  |  |  |  |  |  |  |  |  |  |  |  |  |  |  |  |  |  |  |  |  |
| Settings | | | Ho-Cl phantom (df1 = 1, df2 = n - 2) | | | | | | | | | | | Ho-MS phantom (n = 9, df1 = 1, df2 = 7) | | | | | | | | |  |
| Voltage | Slices | Kernel | Exposure | CTDIvol | Max. | n | R^2^ | m | SE | b | SE | F | p | Exposure | CTDIvol | R^2^ | m | SE | b | SE | F | p |  |
|  |  |  |  |  |  |  |  |  |  |  |  |  |  |  |  |  |  |  |  |  |  |  |  |
| kVp | mm |  | mAs | mGy | mg/mL |  |  | HU | | HU x mL/mg | |  |  | mAs | mGy |  | HU | | HU x mL/mg | |  |  |  |
| 80 | 1 | H31s | 70 | 3.44 | 78.00 | 15 | 0.999 | 11.20 | 11.40 | 36.48 | 36.50 | 2.22E+04 | 2.09E-22 | 76 | 3.73 | 0.990 | 13.11 | 13.40 | 35.38 | 35.38 | 6.60E+02 | 3.47E-08 |  |
| 80 | 1 | H41s | 70 | 3.44 | 78.00 | 15 | 0.999 | 11.25 | 11.47 | 36.55 | 36.56 | 2.08E+04 | 3.19E-22 | 76 | 3.73 | 0.989 | 13.10 | 13.40 | 35.41 | 35.41 | 6.56E+02 | 3.53E-08 |  |
| 80 | 1 | H60s | 70 | 3.44 | 43.60 | 12 | 1.000 | -2.48 | -1.44 | 37.07 | 37.08 | 2.21E+04 | 4.65E-18 | 76 | 3.73 | 0.997 | -3.83 | -2.80 | 39.60 | 39.58 | 2.00E+03 | 7.30E-10 |  |
| 80 | 2 | H31s | 70 | 3.44 | 78.00 | 15 | 0.999 | 10.97 | 11.20 | 36.53 | 36.55 | 2.00E+04 | 4.19E-22 | 76 | 3.73 | 0.989 | 13.32 | 13.67 | 35.24 | 35.24 | 6.19E+02 | 4.32E-08 |  |
| 80 | 2 | H41s | 70 | 3.44 | 78.00 | 15 | 0.999 | 11.16 | 11.42 | 36.57 | 36.59 | 2.01E+04 | 4.03E-22 | 76 | 3.73 | 0.989 | 13.38 | 13.75 | 35.27 | 35.27 | 6.18E+02 | 4.35E-08 |  |
| 80 | 2 | H60s | 70 | 3.44 | 43.60 | 12 | 1.000 | -3.02 | -1.95 | 37.30 | 37.32 | 2.66E+04 | 1.86E-18 | 76 | 3.73 | 0.997 | -3.33 | -2.26 | 39.56 | 39.55 | 1.97E+03 | 7.72E-10 |  |
| 80 | 5 | H31s | 70 | 3.44 | 78.00 | 15 | 0.999 | 11.11 | 11.42 | 36.46 | 36.50 | 2.24E+04 | 2.01E-22 | 76 | 3.73 | 0.989 | 13.96 | 14.46 | 35.24 | 35.23 | 6.52E+02 | 3.61E-08 |  |
| 80 | 5 | H41s | 70 | 3.44 | 78.00 | 15 | 0.999 | 11.37 | 11.67 | 36.52 | 36.56 | 2.34E+04 | 1.50E-22 | 76 | 3.73 | 0.989 | 13.82 | 14.34 | 35.32 | 35.31 | 6.33E+02 | 3.99E-08 |  |
| 80 | 5 | H60s | 70 | 3.44 | 65.00 | 13 | 1.000 | 0.76 | 1.86 | 36.63 | 36.68 | 2.57E+04 | 6.91E-20 | 76 | 3.73 | 0.997 | -3.33 | -2.07 | 39.81 | 39.80 | 2.08E+03 | 6.34E-10 |  |
| 80 | 1 | H31s | 200 | 9.83 | 78.00 | 15 | 0.999 | 11.37 | 11.52 | 36.46 | 36.47 | 2.11E+04 | 2.96E-22 | 200 | 9.83 | 0.989 | 14.11 | 14.34 | 35.31 | 35.30 | 6.17E+02 | 4.38E-08 |  |
| 80 | 1 | H41s | 200 | 9.83 | 78.00 | 15 | 0.999 | 11.60 | 11.77 | 36.50 | 36.52 | 2.12E+04 | 2.87E-22 | 200 | 9.83 | 0.989 | 13.74 | 13.98 | 35.36 | 35.35 | 6.12E+02 | 4.50E-08 |  |
| 80 | 1 | H60s | 200 | 9.83 | 43.60 | 12 | 1.000 | -2.64 | -1.96 | 37.14 | 37.15 | 2.03E+04 | 7.10E-18 | 200 | 9.83 | 0.997 | -2.97 | -2.35 | 39.52 | 39.52 | 1.99E+03 | 7.47E-10 |  |
| 80 | 2 | H31s | 200 | 9.83 | 78.00 | 15 | 0.999 | 11.25 | 11.46 | 36.46 | 36.49 | 2.02E+04 | 3.87E-22 | 200 | 9.83 | 0.989 | 12.74 | 13.03 | 35.34 | 35.35 | 6.06E+02 | 4.65E-08 |  |
| 80 | 2 | H41s | 200 | 9.83 | 78.00 | 15 | 0.999 | 11.78 | 11.98 | 36.46 | 36.49 | 2.19E+04 | 2.34E-22 | 200 | 9.83 | 0.989 | 13.40 | 13.71 | 35.28 | 35.29 | 6.23E+02 | 4.23E-08 |  |
| 80 | 2 | H60s | 200 | 9.83 | 43.60 | 12 | 1.000 | -2.40 | -1.66 | 37.20 | 37.22 | 2.17E+04 | 5.09E-18 | 200 | 9.83 | 0.996 | -3.63 | -2.97 | 39.56 | 39.57 | 1.91E+03 | 8.60E-10 |  |
| 80 | 5 | H31s | 200 | 9.83 | 78.00 | 15 | 1.000 | 11.19 | 11.46 | 36.43 | 36.47 | 2.47E+04 | 1.06E-22 | 200 | 9.83 | 0.989 | 13.37 | 13.85 | 35.31 | 35.30 | 6.07E+02 | 4.61E-08 |  |
| 80 | 5 | H41s | 200 | 9.83 | 78.00 | 15 | 1.000 | 11.26 | 11.51 | 36.49 | 36.54 | 2.42E+04 | 1.19E-22 | 200 | 9.83 | 0.989 | 13.40 | 13.89 | 35.36 | 35.34 | 6.03E+02 | 4.72E-08 |  |
| 80 | 5 | H60s | 200 | 9.83 | 65.00 | 13 | 1.000 | 0.62 | 1.39 | 36.67 | 36.72 | 2.56E+04 | 7.10E-20 | 200 | 9.83 | 0.997 | -3.73 | -2.90 | 39.83 | 39.85 | 2.15E+03 | 5.69E-10 |  |
| 80 | 1 | H31s | 400 | 19.65 | 78.00 | 15 | 0.999 | 11.22 | 11.36 | 36.48 | 36.50 | 2.10E+04 | 2.98E-22 | 400 | 19.65 | 0.988 | 13.71 | 13.93 | 35.35 | 35.34 | 5.90E+02 | 5.12E-08 |  |
| 80 | 1 | H41s | 400 | 19.65 | 78.00 | 15 | 0.999 | 11.31 | 11.45 | 36.56 | 36.58 | 2.02E+04 | 3.95E-22 | 400 | 19.65 | 0.988 | 13.76 | 13.98 | 35.37 | 35.36 | 5.88E+02 | 5.16E-08 |  |
| 80 | 1 | H60s | 400 | 19.65 | 43.60 | 12 | 1.000 | -2.55 | -2.05 | 37.20 | 37.21 | 2.09E+04 | 6.16E-18 | 400 | 19.65 | 0.996 | -3.48 | -3.03 | 39.63 | 39.64 | 1.96E+03 | 7.89E-10 |  |
| 80 | 2 | H31s | 400 | 19.65 | 78.00 | 15 | 0.999 | 11.33 | 11.51 | 36.47 | 36.50 | 2.04E+04 | 3.67E-22 | 400 | 19.65 | 0.988 | 14.29 | 14.58 | 35.20 | 35.20 | 5.75E+02 | 5.59E-08 |  |
| 80 | 2 | H41s | 400 | 19.65 | 78.00 | 15 | 0.999 | 11.47 | 11.66 | 36.55 | 36.58 | 1.96E+04 | 4.80E-22 | 400 | 19.65 | 0.988 | 14.13 | 14.43 | 35.26 | 35.27 | 5.70E+02 | 5.75E-08 |  |
| 80 | 2 | H60s | 400 | 19.65 | 65.00 | 13 | 1.000 | 1.21 | 1.71 | 36.68 | 36.72 | 2.65E+04 | 5.88E-20 | 400 | 19.65 | 0.997 | -2.49 | -1.95 | 39.58 | 39.60 | 1.99E+03 | 7.43E-10 |  |
| 80 | 5 | H31s | 400 | 19.65 | 78.00 | 15 | 0.999 | 12.26 | 12.52 | 36.34 | 36.39 | 2.36E+04 | 1.43E-22 | 400 | 19.65 | 0.989 | 13.76 | 14.22 | 35.29 | 35.28 | 6.31E+02 | 4.04E-08 |  |
| 80 | 5 | H41s | 400 | 19.65 | 78.00 | 15 | 0.999 | 12.24 | 12.50 | 36.45 | 36.50 | 2.00E+04 | 4.16E-22 | 400 | 19.65 | 0.989 | 13.78 | 14.25 | 35.29 | 35.28 | 6.07E+02 | 4.64E-08 |  |
| 80 | 5 | H60s | 400 | 19.65 | 65.00 | 13 | 1.000 | 2.12 | 2.75 | 36.63 | 36.69 | 2.57E+04 | 6.96E-20 | 400 | 19.65 | 0.997 | -3.37 | -2.68 | 39.88 | 39.90 | 2.12E+03 | 5.94E-10 |  |
| 100 | 1 | H31s | 74 | 7.28 | 79.70 | 16 | 1.000 | 8.88 | 9.02 | 35.32 | 35.34 | 3.54E+04 | 3.14E-25 | 79 | 7.77 | 0.989 | 12.67 | 12.89 | 34.03 | 34.03 | 6.17E+02 | 4.36E-08 |  |
| 100 | 1 | H41s | 74 | 7.28 | 79.70 | 16 | 1.000 | 8.98 | 9.14 | 35.42 | 35.44 | 2.95E+04 | 1.12E-24 | 79 | 7.77 | 0.989 | 12.91 | 13.15 | 34.04 | 34.04 | 6.04E+02 | 4.72E-08 |  |
| 100 | 1 | H60s | 74 | 7.28 | 65.00 | 13 | 1.000 | -3.25 | -2.63 | 35.86 | 35.88 | 3.13E+04 | 2.34E-20 | 79 | 7.77 | 0.997 | -5.06 | -4.39 | 38.48 | 38.49 | 2.48E+03 | 3.45E-10 |  |
| 100 | 2 | H31s | 74 | 7.28 | 79.70 | 16 | 1.000 | 8.79 | 8.98 | 35.35 | 35.37 | 2.96E+04 | 1.11E-24 | 79 | 7.77 | 0.988 | 11.81 | 12.10 | 34.08 | 34.09 | 5.94E+02 | 4.99E-08 |  |
| 100 | 2 | H41s | 74 | 7.28 | 79.70 | 16 | 1.000 | 9.13 | 9.33 | 35.40 | 35.42 | 2.94E+04 | 1.16E-24 | 79 | 7.77 | 0.988 | 11.86 | 12.16 | 34.11 | 34.11 | 5.93E+02 | 5.03E-08 |  |
| 100 | 2 | H60s | 74 | 7.28 | 65.00 | 13 | 1.000 | -3.14 | -2.48 | 35.81 | 35.84 | 2.91E+04 | 3.55E-20 | 79 | 7.77 | 0.997 | -4.94 | -4.19 | 38.42 | 38.43 | 2.27E+03 | 4.67E-10 |  |
| 100 | 5 | H31s | 74 | 7.28 | 79.70 | 16 | 1.000 | 9.04 | 9.35 | 35.35 | 35.39 | 2.83E+04 | 1.53E-24 | 79 | 7.77 | 0.989 | 13.21 | 13.65 | 33.94 | 33.93 | 6.17E+02 | 4.37E-08 |  |
| 100 | 5 | H41s | 74 | 7.28 | 79.70 | 16 | 1.000 | 9.37 | 9.63 | 35.40 | 35.43 | 2.93E+04 | 1.20E-24 | 79 | 7.77 | 0.988 | 12.88 | 13.34 | 33.99 | 33.98 | 5.90E+02 | 5.10E-08 |  |
| 100 | 5 | H60s | 74 | 7.28 | 65.00 | 13 | 1.000 | -3.67 | -3.01 | 36.00 | 36.08 | 3.81E+04 | 7.95E-21 | 79 | 7.77 | 0.997 | -4.58 | -3.67 | 38.61 | 38.62 | 2.36E+03 | 4.11E-10 |  |
| 100 | 1 | H31s | 200 | 19.66 | 79.70 | 16 | 1.000 | 8.80 | 8.93 | 35.36 | 35.38 | 3.09E+04 | 8.19E-25 | 200 | 19.66 | 0.988 | 11.91 | 12.11 | 34.09 | 34.09 | 5.62E+02 | 6.03E-08 |  |
| 100 | 1 | H41s | 200 | 19.66 | 79.70 | 16 | 1.000 | 8.87 | 9.01 | 35.43 | 35.44 | 2.87E+04 | 1.36E-24 | 200 | 19.66 | 0.988 | 11.95 | 12.15 | 34.11 | 34.11 | 5.61E+02 | 6.07E-08 |  |
| 100 | 1 | H60s | 200 | 19.66 | 43.60 | 12 | 1.000 | -6.04 | -5.55 | 36.27 | 36.29 | 2.15E+04 | 5.34E-18 | 200 | 19.66 | 0.997 | -5.40 | -4.97 | 38.45 | 38.47 | 2.45E+03 | 3.58E-10 |  |
| 100 | 2 | H31s | 200 | 19.66 | 79.70 | 16 | 1.000 | 8.75 | 8.93 | 35.33 | 35.36 | 3.31E+04 | 5.06E-25 | 200 | 19.66 | 0.988 | 12.00 | 12.26 | 34.05 | 34.06 | 5.81E+02 | 5.40E-08 |  |
| 100 | 2 | H41s | 200 | 19.66 | 79.70 | 16 | 1.000 | 8.88 | 9.06 | 35.41 | 35.43 | 2.94E+04 | 1.16E-24 | 200 | 19.66 | 0.988 | 12.39 | 12.67 | 34.00 | 34.01 | 5.56E+02 | 6.26E-08 |  |
| 100 | 2 | H60s | 200 | 19.66 | 65.00 | 13 | 1.000 | -3.40 | -2.91 | 35.84 | 35.87 | 2.97E+04 | 3.12E-20 | 200 | 19.66 | 0.997 | -4.77 | -4.26 | 38.42 | 38.44 | 2.27E+03 | 4.69E-10 |  |
| 100 | 5 | H31s | 200 | 19.66 | 79.70 | 16 | 1.000 | 9.33 | 9.59 | 35.33 | 35.36 | 3.11E+04 | 7.88E-25 | 200 | 19.66 | 0.989 | 12.20 | 12.62 | 34.04 | 34.03 | 6.05E+02 | 4.69E-08 |  |
| 100 | 5 | H41s | 200 | 19.66 | 79.70 | 16 | 1.000 | 9.34 | 9.64 | 35.42 | 35.45 | 2.69E+04 | 2.14E-24 | 200 | 19.66 | 0.989 | 12.28 | 12.71 | 34.07 | 34.06 | 6.02E+02 | 4.77E-08 |  |
| 100 | 5 | H60s | 200 | 19.66 | 65.00 | 13 | 1.000 | -2.35 | -1.72 | 35.81 | 35.85 | 2.99E+04 | 3.04E-20 | 200 | 19.66 | 0.997 | -5.18 | -4.54 | 38.66 | 38.68 | 2.42E+03 | 3.76E-10 |  |
| 100 | 1 | H31s | 400 | 39.33 | 79.70 | 16 | 1.000 | 9.05 | 9.17 | 35.36 | 35.38 | 3.53E+04 | 3.23E-25 | 400 | 39.33 | 0.988 | 13.49 | 13.68 | 34.04 | 34.03 | 5.61E+02 | 6.09E-08 |  |
| 100 | 1 | H41s | 400 | 39.33 | 79.70 | 16 | 1.000 | 9.10 | 9.24 | 35.47 | 35.48 | 2.91E+04 | 1.25E-24 | 400 | 39.33 | 0.988 | 13.57 | 13.76 | 34.06 | 34.06 | 5.65E+02 | 5.92E-08 |  |
| 100 | 1 | H60s | 400 | 39.33 | 65.00 | 13 | 1.000 | -2.82 | -2.49 | 35.89 | 35.91 | 3.00E+04 | 2.99E-20 | 400 | 39.33 | 0.997 | -3.82 | -3.47 | 38.44 | 38.46 | 2.27E+03 | 4.73E-10 |  |
| 100 | 2 | H31s | 400 | 39.33 | 79.70 | 16 | 1.000 | 9.56 | 9.73 | 35.36 | 35.38 | 2.87E+04 | 1.38E-24 | 400 | 39.33 | 0.988 | 13.61 | 13.86 | 34.00 | 34.01 | 5.82E+02 | 5.35E-08 |  |
| 100 | 2 | H41s | 400 | 39.33 | 79.70 | 16 | 1.000 | 9.83 | 10.01 | 35.41 | 35.43 | 2.88E+04 | 1.35E-24 | 400 | 39.33 | 0.988 | 13.19 | 13.45 | 34.09 | 34.10 | 5.85E+02 | 5.25E-08 |  |
| 100 | 2 | H60s | 400 | 39.33 | 65.00 | 13 | 1.000 | -1.94 | -1.54 | 35.89 | 35.93 | 3.12E+04 | 2.42E-20 | 400 | 39.33 | 0.997 | -3.55 | -3.12 | 38.43 | 38.46 | 2.22E+03 | 5.05E-10 |  |
| 100 | 5 | H31s | 400 | 39.33 | 79.70 | 16 | 1.000 | 9.81 | 10.04 | 35.33 | 35.37 | 3.20E+04 | 6.41E-25 | 400 | 39.33 | 0.989 | 13.55 | 13.97 | 33.98 | 33.98 | 6.12E+02 | 4.50E-08 |  |
| 100 | 5 | H41s | 400 | 39.33 | 79.70 | 16 | 1.000 | 10.07 | 10.33 | 35.42 | 35.46 | 2.71E+04 | 2.03E-24 | 400 | 39.33 | 0.989 | 13.82 | 14.24 | 34.00 | 33.99 | 6.07E+02 | 4.63E-08 |  |
| 100 | 5 | H60s | 400 | 39.33 | 65.00 | 13 | 1.000 | -2.77 | -2.40 | 36.01 | 36.08 | 4.24E+04 | 4.42E-21 | 400 | 39.33 | 0.997 | -3.78 | -3.19 | 38.65 | 38.67 | 2.37E+03 | 4.02E-10 |  |
| 120 | 1 | H31s | 76 | 12.33 | 89.30 | 18 | 0.996 | 25.83 | 25.95 | 29.75 | 29.77 | 4.41E+03 | 5.77E-21 | 81 | 13.14 | 0.988 | 10.56 | 10.77 | 31.28 | 31.27 | 5.71E+02 | 5.73E-08 |  |
| 120 | 1 | H41s | 76 | 12.33 | 89.30 | 18 | 0.996 | 26.37 | 26.47 | 29.80 | 29.82 | 4.34E+03 | 6.48E-21 | 81 | 13.14 | 0.988 | 10.70 | 10.91 | 31.29 | 31.29 | 5.62E+02 | 6.05E-08 |  |
| 120 | 1 | H60s | 76 | 12.33 | 71.30 | 14 | 1.000 | -2.25 | -1.79 | 31.73 | 31.75 | 2.48E+04 | 2.89E-21 | 81 | 13.14 | 0.997 | -6.31 | -5.77 | 35.16 | 35.17 | 2.31E+03 | 4.40E-10 |  |
| 120 | 2 | H31s | 76 | 12.33 | 89.30 | 18 | 0.997 | 25.55 | 25.69 | 29.76 | 29.79 | 4.54E+03 | 4.55E-21 | 81 | 13.14 | 0.988 | 11.19 | 11.45 | 31.13 | 31.14 | 5.74E+02 | 5.62E-08 |  |
| 120 | 2 | H41s | 76 | 12.33 | 89.30 | 18 | 0.996 | 26.11 | 26.25 | 29.83 | 29.85 | 4.24E+03 | 7.90E-21 | 81 | 13.14 | 0.988 | 11.25 | 11.53 | 31.16 | 31.17 | 5.73E+02 | 5.65E-08 |  |
| 120 | 2 | H60s | 76 | 12.33 | 78.00 | 15 | 0.998 | 3.64 | 4.16 | 31.08 | 31.11 | 7.65E+03 | 2.14E-19 | 81 | 13.14 | 0.997 | -5.41 | -4.79 | 35.08 | 35.10 | 2.39E+03 | 3.92E-10 |  |
| 120 | 5 | H31s | 77 | 12.49 | 89.30 | 18 | 0.996 | 26.76 | 26.84 | 29.68 | 29.73 | 4.21E+03 | 8.27E-21 | 81 | 13.14 | 0.989 | 10.35 | 10.76 | 31.25 | 31.24 | 6.17E+02 | 4.37E-08 |  |
| 120 | 5 | H41s | 77 | 12.49 | 89.30 | 18 | 0.996 | 26.49 | 26.60 | 29.77 | 29.81 | 4.07E+03 | 1.08E-20 | 81 | 13.14 | 0.989 | 10.52 | 10.93 | 31.27 | 31.26 | 6.07E+02 | 4.63E-08 |  |
| 120 | 5 | H60s | 77 | 12.49 | 92.50 | 17 | 0.995 | 17.16 | 17.80 | 29.84 | 29.89 | 3.18E+03 | 7.03E-19 | 81 | 13.14 | 0.997 | -6.19 | -5.45 | 35.34 | 35.36 | 2.34E+03 | 4.24E-10 |  |
| 120 | 1 | H31s | 200 | 32.44 | 89.30 | 18 | 0.996 | 25.66 | 25.76 | 29.80 | 29.82 | 4.39E+03 | 5.95E-21 | 200 | 32.44 | 0.988 | 11.17 | 11.36 | 31.22 | 31.22 | 5.70E+02 | 5.75E-08 |  |
| 120 | 1 | H41s | 200 | 32.44 | 89.30 | 18 | 0.996 | 25.89 | 25.98 | 29.85 | 29.86 | 4.40E+03 | 5.87E-21 | 200 | 32.44 | 0.988 | 11.20 | 11.39 | 31.25 | 31.24 | 5.69E+02 | 5.80E-08 |  |
| 120 | 1 | H60s | 200 | 32.44 | 71.30 | 14 | 1.000 | -3.08 | -2.78 | 31.89 | 31.92 | 3.04E+04 | 8.46E-22 | 200 | 32.44 | 0.997 | -5.71 | -5.33 | 35.13 | 35.14 | 2.29E+03 | 4.56E-10 |  |
| 120 | 2 | H31s | 200 | 32.44 | 89.30 | 18 | 0.996 | 26.03 | 26.14 | 29.75 | 29.78 | 4.41E+03 | 5.71E-21 | 200 | 32.44 | 0.988 | 10.70 | 10.95 | 31.18 | 31.19 | 5.69E+02 | 5.79E-08 |  |
| 120 | 2 | H41s | 200 | 32.44 | 89.30 | 18 | 0.996 | 26.33 | 26.45 | 29.80 | 29.82 | 4.30E+03 | 7.00E-21 | 200 | 32.44 | 0.988 | 10.77 | 11.03 | 31.21 | 31.22 | 5.68E+02 | 5.83E-08 |  |
| 120 | 2 | H60s | 200 | 32.44 | 78.00 | 15 | 0.998 | 4.40 | 4.80 | 30.99 | 31.02 | 8.27E+03 | 1.29E-19 | 200 | 32.44 | 0.997 | -5.88 | -5.44 | 35.15 | 35.18 | 2.27E+03 | 4.68E-10 |  |
| 120 | 5 | H31s | 200 | 32.44 | 89.30 | 18 | 0.996 | 26.09 | 26.36 | 29.72 | 29.76 | 4.44E+03 | 5.43E-21 | 200 | 32.44 | 0.989 | 11.02 | 11.41 | 31.21 | 31.20 | 6.28E+02 | 4.11E-08 |  |
| 120 | 5 | H41s | 200 | 32.44 | 89.30 | 18 | 0.996 | 27.31 | 27.42 | 29.71 | 29.76 | 4.22E+03 | 8.11E-21 | 200 | 32.44 | 0.989 | 11.10 | 11.50 | 31.24 | 31.23 | 6.22E+02 | 4.26E-08 |  |
| 120 | 5 | H60s | 200 | 32.44 | 92.50 | 17 | 0.995 | 17.46 | 18.01 | 29.84 | 29.89 | 3.13E+03 | 7.93E-19 | 200 | 32.44 | 0.997 | -5.49 | -4.89 | 35.31 | 35.33 | 2.39E+03 | 3.89E-10 |  |
| 120 | 1 | H31s | 400 | 64.87 | 89.30 | 18 | 0.996 | 25.79 | 25.88 | 29.86 | 29.88 | 4.49E+03 | 4.96E-21 | 400 | 64.87 | 0.988 | 11.57 | 11.76 | 31.21 | 31.20 | 5.66E+02 | 5.91E-08 |  |
| 120 | 1 | H41s | 400 | 64.87 | 89.30 | 18 | 0.996 | 26.36 | 26.45 | 29.89 | 29.90 | 4.31E+03 | 6.91E-21 | 400 | 64.87 | 0.988 | 11.54 | 11.72 | 31.28 | 31.28 | 5.74E+02 | 5.61E-08 |  |
| 120 | 1 | H60s | 400 | 64.87 | 78.00 | 15 | 0.998 | 4.23 | 4.50 | 31.07 | 31.09 | 8.31E+03 | 1.25E-19 | 400 | 64.87 | 0.997 | -5.34 | -5.04 | 35.12 | 35.14 | 2.30E+03 | 4.52E-10 |  |
| 120 | 2 | H31s | 400 | 64.87 | 89.30 | 18 | 0.997 | 26.53 | 26.66 | 29.77 | 29.79 | 4.49E+03 | 4.95E-21 | 400 | 64.87 | 0.988 | 11.46 | 11.72 | 31.11 | 31.12 | 5.72E+02 | 5.67E-08 |  |
| 120 | 2 | H41s | 400 | 64.87 | 89.30 | 18 | 0.996 | 26.72 | 26.84 | 29.84 | 29.86 | 4.32E+03 | 6.71E-21 | 400 | 64.87 | 0.988 | 11.26 | 11.52 | 31.18 | 31.19 | 5.66E+02 | 5.90E-08 |  |
| 120 | 2 | H60s | 400 | 64.87 | 71.30 | 14 | 1.000 | -2.01 | -1.77 | 31.88 | 31.92 | 3.02E+04 | 8.84E-22 | 400 | 64.87 | 0.997 | -5.30 | -4.93 | 35.13 | 35.16 | 2.27E+03 | 4.67E-10 |  |
| 120 | 5 | H31s | 400 | 64.87 | 89.30 | 18 | 0.997 | 25.75 | 26.00 | 29.83 | 29.87 | 4.50E+03 | 4.87E-21 | 400 | 64.87 | 0.989 | 11.48 | 11.88 | 31.14 | 31.13 | 6.26E+02 | 4.17E-08 |  |
| 120 | 5 | H41s | 400 | 64.87 | 89.30 | 18 | 0.996 | 27.20 | 27.26 | 29.81 | 29.85 | 4.23E+03 | 7.96E-21 | 400 | 64.87 | 0.989 | 11.47 | 11.86 | 31.21 | 31.20 | 6.28E+02 | 4.10E-08 |  |
| 120 | 5 | H60s | 400 | 64.87 | 79.70 | 16 | 0.998 | 6.10 | 6.50 | 30.87 | 30.92 | 8.14E+03 | 9.23E-21 | 400 | 64.87 | 0.997 | -5.55 | -5.04 | 35.36 | 35.38 | 2.46E+03 | 3.55E-10 |  |
| Canon Aquilion ONE | | | | | | | | | | | | | | | | | | | | | | |  |
|  |  |  |  |  |  |  |  |  |  |  |  |  |  |  |  |  |  |  |  |  |  |  |  |
| Settings | | | Ho-Cl phantom (df1 = 1, df2 = n - 2) | | | | | | | | | | | Ho-MS phantom (n = 9, df1 = 1, df2 = 7) | | | | | | | | |  |
| Voltage | Slices | Kernel | Exposure | CTDIvol | Max. | n | R^2^ | m | SE | b | SE | F | p | Exposure | CTDIvol | R^2^ | m | SE | b | SE | F | p |  |
|  |  |  |  |  |  |  |  |  |  |  |  |  |  |  |  |  |  |  |  |  |  |  |  |
| kVp | mm |  | mAs | mGy | mg/mL |  |  | HU | | HU x mL/mg | |  |  | mAs | mGy |  | HU | | HU x mL/mg | |  |  |  |
| 80 | 1 | Brain | 150 | 3.30 |  | 19 | 0.998 | 34.12 | 34.15 | 46.21 | 46.25 | 3.48E+03 | 1.20E-24 | 160 | 3.60 | 1.000 | 26.17 | 26.31 | 46.52 | 46.62 | 8.83E+04 | 2.76E-15 |  |
| 80 | 1 | Brain+ | 150 | 3.30 |  | 19 | 0.998 | 28.77 | 28.76 | 45.78 | 45.81 | 3.48E+03 | 1.19E-24 | 160 | 3.60 | 1.000 | 24.62 | 24.71 | 45.15 | 45.25 | 1.81E+04 | 7.04E-13 |  |
| 80 | 1 | Bone | 150 | 3.30 |  | 19 | 0.997 | 10.77 | 10.71 | 38.77 | 38.85 | 2.27E+03 | 4.34E-23 | 160 | 3.60 | 0.998 | 18.26 | 18.52 | 41.17 | 41.32 | 4.86E+03 | 6.65E-11 |  |
| 80 | 2 | Brain | 150 | 3.30 |  | 19 | 0.998 | 32.78 | 32.62 | 46.28 | 46.33 | 3.54E+03 | 1.03E-24 | 160 | 3.60 | 1.000 | 25.94 | 26.15 | 46.57 | 46.67 | 7.69E+04 | 4.45E-15 |  |
| 80 | 2 | Brain+ | 150 | 3.30 |  | 19 | 0.998 | 24.97 | 24.74 | 45.96 | 46.01 | 3.54E+03 | 1.03E-24 | 160 | 3.60 | 0.999 | 24.38 | 24.49 | 45.18 | 45.29 | 1.70E+04 | 8.69E-13 |  |
| 80 | 2 | Bone | 150 | 3.30 |  | 19 | 0.998 | 11.60 | 11.45 | 38.51 | 38.61 | 2.77E+03 | 8.11E-24 | 160 | 3.60 | 0.998 | 18.01 | 18.31 | 41.20 | 41.37 | 5.16E+03 | 5.35E-11 |  |
| 80 | 5 | Brain | 150 | 3.30 |  | 19 | 0.998 | 33.83 | 33.83 | 46.24 | 46.32 | 3.48E+03 | 1.21E-24 | 160 | 3.60 | 1.000 | 25.82 | 26.14 | 46.56 | 46.66 | 7.99E+04 | 3.90E-15 |  |
| 80 | 5 | Brain+ | 150 | 3.30 |  | 19 | 0.998 | 25.18 | 24.76 | 45.97 | 46.07 | 3.44E+03 | 1.31E-24 | 160 | 3.60 | 0.999 | 24.26 | 24.42 | 45.18 | 45.29 | 1.71E+04 | 8.41E-13 |  |
| 80 | 5 | Bone | 150 | 3.30 |  | 19 | 0.997 | 10.41 | 9.88 | 38.84 | 39.03 | 2.44E+03 | 2.37E-23 | 160 | 3.60 | 0.998 | 17.96 | 18.32 | 41.20 | 41.38 | 5.10E+03 | 5.59E-11 |  |
| 80 | 1 | Brain | 440 | 9.90 |  | 19 | 0.998 | 27.86 | 27.67 | 46.41 | 46.45 | 3.27E+03 | 2.00E-24 | 440 | 9.90 | 1.000 | 24.37 | 24.49 | 46.15 | 46.23 | 4.40E+04 | 3.10E-14 |  |
| 80 | 1 | Brain+ | 440 | 9.90 |  | 19 | 0.998 | 21.65 | 21.51 | 45.99 | 46.03 | 3.32E+03 | 1.75E-24 | 440 | 9.90 | 0.999 | 22.90 | 22.98 | 44.80 | 44.88 | 1.24E+04 | 2.62E-12 |  |
| 80 | 1 | Bone | 440 | 9.90 |  | 19 | 0.997 | 10.07 | 9.92 | 38.80 | 38.88 | 2.55E+03 | 1.64E-23 | 440 | 9.90 | 0.998 | 17.07 | 17.24 | 41.14 | 41.27 | 5.26E+03 | 4.96E-11 |  |
| 80 | 2 | Brain | 440 | 9.90 |  | 19 | 0.998 | 27.29 | 27.01 | 46.41 | 46.47 | 3.43E+03 | 1.34E-24 | 440 | 9.90 | 1.000 | 24.12 | 24.30 | 46.17 | 46.26 | 4.32E+04 | 3.29E-14 |  |
| 80 | 2 | Brain+ | 440 | 9.90 |  | 19 | 0.998 | 21.52 | 21.24 | 45.99 | 46.04 | 3.31E+03 | 1.82E-24 | 440 | 9.90 | 0.999 | 22.64 | 22.74 | 44.81 | 44.91 | 1.21E+04 | 2.80E-12 |  |
| 80 | 2 | Bone | 440 | 9.90 |  | 19 | 0.998 | 11.88 | 11.32 | 38.66 | 38.77 | 2.98E+03 | 4.36E-24 | 440 | 9.90 | 0.999 | 16.78 | 16.99 | 41.17 | 41.32 | 5.68E+03 | 3.79E-11 |  |
| 80 | 5 | Brain | 440 | 9.90 |  | 19 | 0.998 | 27.91 | 27.44 | 46.43 | 46.52 | 3.26E+03 | 2.07E-24 | 440 | 9.90 | 1.000 | 24.01 | 24.28 | 46.18 | 46.28 | 4.25E+04 | 3.48E-14 |  |
| 80 | 5 | Brain+ | 440 | 9.90 |  | 19 | 0.998 | 21.91 | 21.46 | 46.00 | 46.09 | 3.30E+03 | 1.85E-24 | 440 | 9.90 | 0.999 | 22.52 | 22.68 | 44.82 | 44.93 | 1.20E+04 | 2.85E-12 |  |
| 80 | 5 | Bone | 440 | 9.90 |  | 19 | 0.997 | 9.95 | 9.42 | 38.85 | 39.02 | 2.54E+03 | 1.67E-23 | 440 | 9.90 | 0.998 | 16.75 | 17.02 | 41.16 | 41.32 | 5.37E+03 | 4.61E-11 |  |
| 80 | 1 | Brain | 800 | 18.10 |  | 19 | 0.998 | 28.89 | 28.69 | 46.36 | 46.40 | 3.47E+03 | 1.22E-24 | 800 | 18.10 | 1.000 | 24.26 | 24.38 | 46.30 | 46.37 | 6.60E+04 | 7.48E-15 |  |
| 80 | 1 | Brain+ | 800 | 18.10 |  | 19 | 0.998 | 23.31 | 23.09 | 45.93 | 45.97 | 3.27E+03 | 2.00E-24 | 800 | 18.10 | 0.999 | 22.78 | 22.85 | 44.96 | 45.04 | 1.47E+04 | 1.42E-12 |  |
| 80 | 1 | Bone | 800 | 18.10 |  | 19 | 0.997 | 8.74 | 8.41 | 39.06 | 39.14 | 2.24E+03 | 4.87E-23 | 800 | 18.10 | 0.998 | 17.51 | 17.66 | 41.34 | 41.45 | 5.54E+03 | 4.15E-11 |  |
| 80 | 2 | Brain | 800 | 18.10 |  | 19 | 0.998 | 28.26 | 27.96 | 46.36 | 46.41 | 3.47E+03 | 1.21E-24 | 800 | 18.10 | 1.000 | 24.03 | 24.20 | 46.31 | 46.39 | 6.25E+04 | 9.04E-15 |  |
| 80 | 2 | Brain+ | 800 | 18.10 |  | 19 | 0.998 | 22.69 | 22.36 | 45.94 | 45.99 | 3.29E+03 | 1.90E-24 | 800 | 18.10 | 0.999 | 22.52 | 22.62 | 44.97 | 45.06 | 1.42E+04 | 1.60E-12 |  |
| 80 | 2 | Bone | 800 | 18.10 |  | 19 | 0.997 | 10.07 | 9.69 | 38.95 | 39.06 | 2.50E+03 | 1.94E-23 | 800 | 18.10 | 0.999 | 17.40 | 17.57 | 41.37 | 41.50 | 5.75E+03 | 3.64E-11 |  |
| 80 | 5 | Brain | 800 | 18.10 |  | 19 | 0.998 | 28.84 | 28.35 | 46.38 | 46.47 | 3.46E+03 | 1.25E-24 | 800 | 18.10 | 1.000 | 23.88 | 24.15 | 46.33 | 46.41 | 6.12E+04 | 9.72E-15 |  |
| 80 | 5 | Brain+ | 800 | 18.10 |  | 19 | 0.998 | 23.11 | 22.58 | 45.95 | 46.03 | 3.25E+03 | 2.11E-24 | 800 | 18.10 | 0.999 | 22.39 | 22.53 | 44.98 | 45.08 | 1.40E+04 | 1.67E-12 |  |
| 80 | 5 | Bone | 800 | 18.10 |  | 19 | 0.997 | 11.00 | 10.45 | 39.04 | 39.22 | 2.47E+03 | 2.18E-23 | 800 | 18.10 | 0.999 | 17.26 | 17.50 | 41.35 | 41.50 | 5.77E+03 | 3.60E-11 |  |
| 100 | 1 | Brain | 140 | 7.10 |  | 19 | 0.997 | 44.39 | 43.92 | 41.57 | 41.62 | 2.47E+03 | 2.21E-23 | 150 | 7.60 | 1.000 | 23.88 | 24.00 | 43.33 | 43.42 | 2.97E+04 | 1.24E-13 |  |
| 100 | 1 | Brain+ | 140 | 7.10 |  | 19 | 0.997 | 33.52 | 33.53 | 41.42 | 41.44 | 2.59E+03 | 1.46E-23 | 150 | 7.60 | 1.000 | 21.39 | 21.47 | 42.27 | 42.37 | 1.85E+04 | 6.40E-13 |  |
| 100 | 1 | Bone | 140 | 7.10 |  | 19 | 0.998 | 20.49 | 20.51 | 36.84 | 36.90 | 2.93E+03 | 5.09E-24 | 150 | 7.60 | 0.998 | 13.67 | 13.84 | 40.65 | 40.81 | 5.40E+03 | 4.39E-11 |  |
| 100 | 2 | Brain | 140 | 7.10 |  | 19 | 0.997 | 43.94 | 43.26 | 41.58 | 41.65 | 2.51E+03 | 1.96E-23 | 150 | 7.60 | 1.000 | 23.71 | 23.89 | 43.36 | 43.46 | 3.16E+04 | 9.97E-14 |  |
| 100 | 2 | Brain+ | 140 | 7.10 |  | 19 | 0.998 | 32.78 | 32.75 | 41.42 | 41.46 | 2.72E+03 | 9.77E-24 | 150 | 7.60 | 1.000 | 21.23 | 21.34 | 42.29 | 42.40 | 1.89E+04 | 5.88E-13 |  |
| 100 | 2 | Bone | 140 | 7.10 |  | 19 | 0.998 | 21.02 | 21.05 | 36.85 | 36.94 | 2.72E+03 | 9.64E-24 | 150 | 7.60 | 0.998 | 13.57 | 13.79 | 40.66 | 40.83 | 5.54E+03 | 3.99E-11 |  |
| 100 | 5 | Brain | 140 | 7.10 |  | 19 | 0.997 | 44.34 | 43.24 | 41.56 | 41.67 | 2.44E+03 | 2.48E-23 | 150 | 7.60 | 1.000 | 23.57 | 23.85 | 43.35 | 43.46 | 3.09E+04 | 1.07E-13 |  |
| 100 | 5 | Brain+ | 140 | 7.10 |  | 19 | 0.997 | 33.25 | 33.18 | 41.42 | 41.48 | 2.59E+03 | 1.46E-23 | 150 | 7.60 | 1.000 | 21.07 | 21.24 | 42.29 | 42.40 | 1.87E+04 | 6.12E-13 |  |
| 100 | 5 | Bone | 140 | 7.10 |  | 19 | 0.998 | 20.63 | 20.47 | 36.89 | 37.02 | 2.99E+03 | 4.24E-24 | 150 | 7.60 | 0.998 | 13.44 | 13.74 | 40.65 | 40.83 | 5.54E+03 | 4.00E-11 |  |
| 100 | 1 | Brain | 390 | 19.70 |  | 19 | 0.997 | 37.05 | 37.07 | 41.70 | 41.72 | 2.56E+03 | 1.60E-23 | 390 | 19.70 | 1.000 | 22.11 | 22.23 | 43.31 | 43.39 | 3.02E+04 | 1.15E-13 |  |
| 100 | 1 | Brain+ | 390 | 19.70 |  | 19 | 0.998 | 30.97 | 30.91 | 41.32 | 41.35 | 2.64E+03 | 1.25E-23 | 390 | 19.70 | 1.000 | 19.45 | 19.53 | 42.26 | 42.36 | 1.80E+04 | 6.79E-13 |  |
| 100 | 1 | Bone | 390 | 19.70 |  | 19 | 0.998 | 20.95 | 20.94 | 36.87 | 36.92 | 2.71E+03 | 9.88E-24 | 390 | 19.70 | 0.998 | 13.31 | 13.44 | 40.69 | 40.85 | 5.54E+03 | 3.99E-11 |  |
| 100 | 2 | Brain | 390 | 19.70 |  | 19 | 0.997 | 36.85 | 36.77 | 41.71 | 41.74 | 2.59E+03 | 1.48E-23 | 390 | 19.70 | 1.000 | 21.89 | 22.05 | 43.33 | 43.42 | 3.39E+04 | 7.61E-14 |  |
| 100 | 2 | Brain+ | 390 | 19.70 |  | 19 | 0.998 | 30.71 | 30.65 | 41.33 | 41.36 | 2.66E+03 | 1.17E-23 | 390 | 19.70 | 1.000 | 19.23 | 19.34 | 42.29 | 42.39 | 1.86E+04 | 6.13E-13 |  |
| 100 | 2 | Bone | 390 | 19.70 |  | 19 | 0.998 | 20.47 | 20.41 | 36.86 | 36.94 | 2.72E+03 | 9.67E-24 | 390 | 19.70 | 0.999 | 13.17 | 13.35 | 40.71 | 40.89 | 5.78E+03 | 3.43E-11 |  |
| 100 | 5 | Brain | 390 | 19.70 |  | 19 | 0.997 | 36.89 | 36.91 | 41.70 | 41.75 | 2.53E+03 | 1.82E-23 | 390 | 19.70 | 1.000 | 21.77 | 22.03 | 43.33 | 43.42 | 3.25E+04 | 8.84E-14 |  |
| 100 | 5 | Brain+ | 390 | 19.70 |  | 19 | 0.998 | 30.88 | 30.73 | 41.32 | 41.37 | 2.60E+03 | 1.43E-23 | 390 | 19.70 | 1.000 | 19.10 | 19.26 | 42.29 | 42.40 | 1.85E+04 | 6.18E-13 |  |
| 100 | 5 | Bone | 390 | 19.70 |  | 19 | 0.998 | 19.82 | 19.56 | 36.86 | 36.99 | 2.90E+03 | 5.54E-24 | 390 | 19.70 | 0.999 | 13.09 | 13.34 | 40.69 | 40.87 | 5.63E+03 | 3.77E-11 |  |
| 100 | 1 | Brain | 770 | 39.30 |  | 19 | 0.998 | 36.77 | 36.66 | 41.81 | 41.83 | 2.64E+03 | 1.24E-23 | 770 | 39.30 | 1.000 | 22.88 | 23.00 | 43.29 | 43.37 | 3.04E+04 | 1.13E-13 |  |
| 100 | 1 | Brain+ | 770 | 39.30 |  | 19 | 0.998 | 30.69 | 30.64 | 41.42 | 41.44 | 2.68E+03 | 1.10E-23 | 770 | 39.30 | 1.000 | 20.27 | 20.35 | 42.26 | 42.34 | 1.75E+04 | 7.58E-13 |  |
| 100 | 1 | Bone | 770 | 39.30 |  | 19 | 0.997 | 18.73 | 18.58 | 37.19 | 37.26 | 2.31E+03 | 3.85E-23 | 770 | 39.30 | 0.999 | 13.87 | 13.98 | 40.78 | 40.90 | 6.05E+03 | 2.95E-11 |  |
| 100 | 2 | Brain | 770 | 39.30 |  | 19 | 0.998 | 36.27 | 36.13 | 41.82 | 41.85 | 2.65E+03 | 1.22E-23 | 770 | 39.30 | 1.000 | 22.65 | 22.82 | 43.32 | 43.40 | 3.18E+04 | 9.65E-14 |  |
| 100 | 2 | Brain+ | 770 | 39.30 |  | 19 | 0.998 | 30.36 | 30.27 | 41.42 | 41.45 | 2.60E+03 | 1.42E-23 | 770 | 39.30 | 1.000 | 20.05 | 20.16 | 42.27 | 42.36 | 1.80E+04 | 6.92E-13 |  |
| 100 | 2 | Bone | 770 | 39.30 |  | 19 | 0.997 | 17.88 | 17.56 | 37.15 | 37.24 | 2.40E+03 | 2.72E-23 | 770 | 39.30 | 0.999 | 13.68 | 13.84 | 40.79 | 40.94 | 6.11E+03 | 2.85E-11 |  |
| 100 | 5 | Brain | 770 | 39.30 |  | 19 | 0.998 | 36.45 | 36.21 | 41.81 | 41.86 | 2.64E+03 | 1.26E-23 | 770 | 39.30 | 1.000 | 22.54 | 22.79 | 43.32 | 43.41 | 3.13E+04 | 1.02E-13 |  |
| 100 | 5 | Brain+ | 770 | 39.30 |  | 19 | 0.998 | 30.29 | 30.17 | 41.43 | 41.48 | 2.67E+03 | 1.11E-23 | 770 | 39.30 | 1.000 | 19.91 | 20.08 | 42.29 | 42.38 | 1.79E+04 | 7.05E-13 |  |
| 100 | 5 | Bone | 770 | 39.30 |  | 19 | 0.997 | 18.49 | 18.08 | 37.22 | 37.37 | 2.30E+03 | 3.89E-23 | 770 | 39.30 | 0.999 | 13.63 | 13.85 | 40.78 | 40.93 | 6.08E+03 | 2.88E-11 |  |
| 120 | 1 | Brain | 140 | 12.50 |  | 19 | 0.997 | 34.51 | 34.54 | 34.62 | 34.64 | 2.52E+03 | 1.82E-23 | 150 | 13.30 | 0.999 | 21.17 | 21.31 | 38.13 | 38.21 | 6.45E+03 | 2.58E-11 |  |
| 120 | 1 | Brain+ | 140 | 12.50 |  | 19 | 0.998 | 27.23 | 27.18 | 34.34 | 34.36 | 2.60E+03 | 1.40E-23 | 150 | 13.30 | 0.999 | 18.19 | 18.27 | 37.29 | 37.37 | 6.06E+03 | 3.11E-11 |  |
| 120 | 1 | Bone | 140 | 12.50 |  | 19 | 0.997 | 26.36 | 26.44 | 31.74 | 31.80 | 2.56E+03 | 1.60E-23 | 150 | 13.30 | 0.999 | 10.46 | 10.62 | 37.64 | 37.78 | 5.72E+03 | 3.50E-11 |  |
| 120 | 2 | Brain | 140 | 12.50 |  | 19 | 0.997 | 34.30 | 34.35 | 34.63 | 34.66 | 2.55E+03 | 1.70E-23 | 150 | 13.30 | 0.999 | 20.97 | 21.17 | 38.14 | 38.22 | 6.63E+03 | 2.34E-11 |  |
| 120 | 2 | Brain+ | 140 | 12.50 |  | 19 | 0.998 | 26.90 | 26.80 | 34.35 | 34.38 | 2.65E+03 | 1.21E-23 | 150 | 13.30 | 0.999 | 18.00 | 18.11 | 37.29 | 37.38 | 6.21E+03 | 2.85E-11 |  |
| 120 | 2 | Bone | 140 | 12.50 |  | 19 | 0.998 | 22.92 | 22.71 | 31.80 | 31.88 | 2.66E+03 | 1.16E-23 | 150 | 13.30 | 0.999 | 10.33 | 10.54 | 37.65 | 37.81 | 5.89E+03 | 3.14E-11 |  |
| 120 | 5 | Brain | 140 | 12.50 |  | 19 | 0.997 | 34.37 | 34.41 | 34.61 | 34.66 | 2.48E+03 | 2.09E-23 | 150 | 13.30 | 0.999 | 20.82 | 21.13 | 38.14 | 38.23 | 6.60E+03 | 2.37E-11 |  |
| 120 | 5 | Brain+ | 140 | 12.50 |  | 19 | 0.998 | 26.93 | 26.77 | 34.33 | 34.38 | 2.60E+03 | 1.43E-23 | 150 | 13.30 | 0.999 | 17.85 | 18.01 | 37.29 | 37.39 | 6.17E+03 | 2.92E-11 |  |
| 120 | 5 | Bone | 140 | 12.50 |  | 19 | 0.997 | 26.02 | 26.03 | 31.76 | 31.88 | 2.57E+03 | 1.57E-23 | 150 | 13.30 | 0.999 | 10.26 | 10.54 | 37.64 | 37.80 | 5.78E+03 | 3.35E-11 |  |
| 120 | 1 | Brain | 360 | 32.40 |  | 19 | 0.997 | 35.14 | 35.18 | 34.68 | 34.70 | 2.58E+03 | 1.51E-23 | 360 | 32.40 | 0.999 | 21.34 | 21.47 | 38.19 | 38.26 | 6.63E+03 | 2.35E-11 |  |
| 120 | 1 | Brain+ | 360 | 32.40 |  | 19 | 0.998 | 28.95 | 28.95 | 34.36 | 34.38 | 2.62E+03 | 1.31E-23 | 360 | 32.40 | 0.999 | 18.45 | 18.53 | 37.34 | 37.41 | 6.16E+03 | 2.95E-11 |  |
| 120 | 1 | Bone | 360 | 32.40 |  | 19 | 0.997 | 25.30 | 25.22 | 32.01 | 32.07 | 2.27E+03 | 4.51E-23 | 360 | 32.40 | 0.999 | 10.64 | 10.76 | 37.74 | 37.86 | 6.54E+03 | 2.18E-11 |  |
| 120 | 2 | Brain | 360 | 32.40 |  | 19 | 0.997 | 34.97 | 35.02 | 34.68 | 34.71 | 2.50E+03 | 2.01E-23 | 360 | 32.40 | 0.999 | 21.14 | 21.33 | 38.19 | 38.27 | 6.83E+03 | 2.11E-11 |  |
| 120 | 2 | Brain+ | 360 | 32.40 |  | 19 | 0.997 | 28.83 | 28.74 | 34.34 | 34.37 | 2.41E+03 | 2.64E-23 | 360 | 32.40 | 0.999 | 18.25 | 18.36 | 37.32 | 37.41 | 6.35E+03 | 2.64E-11 |  |
| 120 | 2 | Bone | 360 | 32.40 |  | 19 | 0.997 | 26.81 | 26.13 | 31.91 | 32.02 | 2.37E+03 | 3.05E-23 | 360 | 32.40 | 0.999 | 10.42 | 10.59 | 37.74 | 37.88 | 6.73E+03 | 1.97E-11 |  |
| 120 | 5 | Brain | 360 | 32.40 |  | 19 | 0.997 | 34.68 | 34.75 | 34.68 | 34.73 | 2.58E+03 | 1.53E-23 | 360 | 32.40 | 0.999 | 21.03 | 21.33 | 38.19 | 38.28 | 6.80E+03 | 2.14E-11 |  |
| 120 | 5 | Brain+ | 360 | 32.40 |  | 19 | 0.998 | 28.47 | 28.44 | 34.35 | 34.40 | 2.62E+03 | 1.33E-23 | 360 | 32.40 | 0.999 | 18.14 | 18.30 | 37.33 | 37.42 | 6.27E+03 | 2.77E-11 |  |
| 120 | 5 | Bone | 360 | 32.40 |  | 19 | 0.997 | 25.06 | 24.77 | 32.02 | 32.16 | 2.27E+03 | 4.51E-23 | 360 | 32.40 | 0.999 | 10.36 | 10.58 | 37.74 | 37.89 | 6.69E+03 | 2.01E-11 |  |
| 120 | 1 | Brain | 720 | 64.80 |  | 19 | 0.997 | 30.36 | 30.31 | 34.68 | 34.71 | 2.34E+03 | 3.45E-23 | 720 | 64.80 | 0.997 | 14.14 | 14.26 | 37.62 | 37.68 | 3.04E+03 | 3.31E-10 |  |
| 120 | 1 | Brain+ | 720 | 64.80 |  | 19 | 0.997 | 24.38 | 24.33 | 34.35 | 34.37 | 2.42E+03 | 2.56E-23 | 720 | 64.80 | 0.997 | 10.74 | 10.83 | 36.85 | 36.92 | 2.50E+03 | 6.34E-10 |  |
| 120 | 1 | Bone | 720 | 64.80 |  | 19 | 0.997 | 21.80 | 21.72 | 31.98 | 32.03 | 1.96E+03 | 1.52E-22 | 720 | 64.80 | 0.998 | 3.73 | 3.87 | 37.25 | 37.37 | 3.30E+03 | 2.20E-10 |  |
| 120 | 2 | Brain | 720 | 64.80 |  | 19 | 0.997 | 29.91 | 29.90 | 34.70 | 34.73 | 2.37E+03 | 3.08E-23 | 720 | 64.80 | 0.997 | 13.87 | 14.04 | 37.71 | 37.79 | 3.16E+03 | 2.88E-10 |  |
| 120 | 2 | Brain+ | 720 | 64.80 |  | 19 | 0.997 | 24.07 | 24.02 | 34.36 | 34.39 | 2.45E+03 | 2.37E-23 | 720 | 64.80 | 0.997 | 10.52 | 10.66 | 36.92 | 37.00 | 2.60E+03 | 5.52E-10 |  |
| 120 | 2 | Bone | 720 | 64.80 |  | 19 | 0.997 | 20.43 | 20.19 | 32.03 | 32.11 | 2.40E+03 | 2.72E-23 | 720 | 64.80 | 0.998 | 3.78 | 3.97 | 37.28 | 37.42 | 3.48E+03 | 1.84E-10 |  |
| 120 | 5 | Brain | 720 | 64.80 |  | 19 | 0.997 | 30.20 | 30.15 | 34.67 | 34.73 | 2.30E+03 | 4.06E-23 | 720 | 64.80 | 0.997 | 13.85 | 14.12 | 37.64 | 37.72 | 3.09E+03 | 3.10E-10 |  |
| 120 | 5 | Brain+ | 720 | 64.80 |  | 19 | 0.997 | 24.26 | 24.14 | 34.34 | 34.39 | 2.38E+03 | 2.98E-23 | 720 | 64.80 | 0.997 | 10.43 | 10.65 | 36.86 | 36.95 | 2.53E+03 | 6.01E-10 |  |
| 120 | 5 | Bone | 720 | 64.80 |  | 19 | 0.997 | 21.71 | 21.48 | 31.99 | 32.12 | 1.97E+03 | 1.46E-22 | 720 | 64.80 | 0.997 | 3.43 | 3.72 | 37.26 | 37.40 | 3.24E+03 | 2.35E-10 |  |

**Supplementary Table 6.** Descriptive statistics and test results for holmium-166 microspheres injections, quantification, recovery, and volume fractions after injection in phantoms and veterinary patients.

| **DESCRIPTIVES** | | | | | | | | | | | | | | | |  |  |
| --- | --- | --- | --- | --- | --- | --- | --- | --- | --- | --- | --- | --- | --- | --- | --- | --- | --- |
|  |  |  |  |  |  |  |  |  |  |  |  |  |  |  |  |  | |
| No. | Descriptives | | | | Shapiro-Wilk | | | Descriptives | | | | Shapiro-Wilk | | | |  | |
|  | Mean | SD | Median | IQR | W | df | p | Mean | SD | Median | IQR | W | df | p | |  | |
| Phantom injections (mg) | | | | | | | | Phantom injections efficacy (%) | | | | | | | |  | |
| 1 | 4.82 | 2.74 | 3.65 | 4.54 | 0.880 | 5 | 0.308 | 0.64 | 0.37 | 0.49 | 0.61 | 0.878 | 5 | 0.300 | |  | |
| 2 | 5.32 | 2.75 | 5.29 | 5.06 | 0.997 | 5 | 0.997 | 0.71 | 0.37 | 0.71 | 0.68 | 0.996 | 5 | 0.996 | |  | |
| 3 | 8.60 | 2.75 | 7.27 | 5.04 | 0.912 | 5 | 0.478 | 1.15 | 0.36 | 0.97 | 0.67 | 0.910 | 5 | 0.468 | |  | |
| 4 | 12.81 | 4.05 | 13.11 | 7.44 | 0.993 | 5 | 0.990 | 0.85 | 0.27 | 0.87 | 0.50 | 0.994 | 5 | 0.992 | |  | |
| 5 | 9.69 | 3.16 | 7.80 | 5.87 | 0.798 | 5 | 0.077 | 0.65 | 0.21 | 0.52 | 0.39 | 0.800 | 5 | 0.081 | |  | |
| Phantom volume reference VOI pre (mL) | | | | | | | | Phantom volume reference VOI post (mL) | | | | | | | |  | |
| 1 | 58.79 | 0.29 | 58.92 | 0.56 | 0.823 | 5 | 0.123 | 55.15 | 1.71 | 54.08 | 3.17 | 0.784 | 5 | 0.060 | |  | |
| 2 | 15.67 | 0.99 | 15.53 | 1.79 | 0.979 | 5 | 0.932 | 12.66 | 1.01 | 13.26 | 1.90 | 0.781 | 5 | 0.056 | |  | |
| 3 | 19.79 | 2.94 | 19.93 | 5.01 | 0.932 | 5 | 0.611 | 16.82 | 2.51 | 16.26 | 4.00 | 0.931 | 5 | 0.605 | |  | |
| 4 | 55.65 | 0.90 | 55.53 | 1.73 | 0.921 | 5 | 0.537 | 41.80 | 4.14 | 41.84 | 6.78 | 0.923 | 5 | 0.551 | |  | |
| 5 | 17.73 | 1.92 | 17.33 | 2.80 | 0.775 | 5 | **0.050** | 11.72 | 2.96 | 12.81 | 5.73 | 0.861 | 5 | 0.232 | |  | |
| 1 and 4 | 57.22 | 1.77 | 57.51 | 3.51 | 0.867 | 10 | 0.092 | 48.48 | 7.64 | 51.08 | 13.47 | 0.875 | 10 | 0.113 | |  | |
| 2, 3 and 5 | 17.73 | 2.61 | 16.89 | 3.69 | 0.889 | 15 | 0.066 | 13.73 | 3.14 | 13.54 | 4.44 | 0.958 | 15 | 0.658 | |  | |
| Phantom volume quantification VOI (mL) | | | | | | | | Phantom volume small quantification VOI (mL) | | | | | | | |  | |
| 1 | 58.75 | 0.72 | 58.94 | 1.04 | 0.733 | 5 | **0.021** | 3.60 | 1.68 | 3.69 | 3.34 | 0.899 | 5 | 0.405 | |  | |
| 2 | 15.75 | 0.95 | 15.70 | 1.72 | 0.978 | 5 | 0.924 | 3.09 | 1.21 | 3.50 | 2.04 | 0.892 | 5 | 0.367 | |  | |
| 3 | 20.03 | 2.80 | 20.01 | 4.68 | 0.885 | 5 | 0.330 | 3.21 | 0.79 | 3.57 | 1.29 | 0.807 | 5 | 0.092 | |  | |
| 4 | 55.55 | 1.11 | 55.52 | 2.16 | 0.920 | 5 | 0.532 | 13.75 | 4.95 | 13.68 | 8.94 | 0.972 | 5 | 0.889 | |  | |
| 5 | 18.20 | 2.16 | 17.70 | 3.03 | 0.791 | 5 | 0.068 | 6.48 | 3.28 | 8.22 | 6.21 | 0.808 | 5 | 0.094 | |  | |
| 1 and 4 | 57.15 | 1.90 | 57.09 | 3.65 | 0.900 | 10 | 0.222 | 8.67 | 6.39 | 5.93 | 11.21 | 0.896 | 10 | 0.199 | |  | |
| 2, 3 and 5 | 17.99 | 2.67 | 17.43 | 3.71 | 0.906 | 15 | 0.119 | 4.26 | 2.51 | 3.57 | 1.39 | 0.799 | 15 | **0.004** | |  | |
| Phantom radiodensity reference VOI pre (HU) | | | | | | | | Phantom radiodensity reference VOI post (HU) | | | | | | | |  | |
| 1 | 3.74 | 0.21 | 3.65 | 0.41 | 0.864 | 5 | 0.242 | 4.16 | 0.29 | 4.22 | 0.46 | 0.811 | 5 | 0.099 | |  | |
| 1 80 kVp | 4.02 | 0.30 | 3.89 | 0.58 | 0.874 | 5 | 0.281 | 4.38 | 0.33 | 4.42 | 0.59 | 0.956 | 5 | 0.780 | |  | |
| 2 | 69.48 | 1.07 | 69.41 | 1.91 | 0.956 | 5 | 0.778 | 58.59 | 1.99 | 59.30 | 3.43 | 0.931 | 5 | 0.606 | |  | |
| 3 | 58.90 | 3.42 | 57.88 | 6.22 | 0.927 | 5 | 0.577 | 55.46 | 1.76 | 55.21 | 3.09 | 0.911 | 5 | 0.473 | |  | |
| 3 80 kVp | 61.93 | 3.42 | 60.88 | 6.21 | 0.930 | 5 | 0.597 | 57.91 | 1.75 | 57.62 | 3.05 | 0.914 | 5 | 0.494 | |  | |
| 4 | 3.86 | 0.29 | 3.86 | 0.48 | 0.871 | 5 | 0.271 | 4.94 | 0.51 | 5.01 | 0.81 | 0.981 | 5 | 0.940 | |  | |
| 5 | 58.03 | 1.94 | 57.21 | 3.68 | 0.899 | 5 | 0.402 | 53.48 | 2.83 | 53.54 | 5.08 | 0.972 | 5 | 0.886 | |  | |
| Phantom radiodensity quantification VOI (HU) | | | | | | | |  | | | | | | | |  | |
| 1 | 3.97 | 0.32 | 4.07 | 0.50 | 0.850 | 5 | 0.194 |  |  |  |  |  |  |  |  |  | |
| 1 80 kVp | 4.14 | 0.41 | 4.18 | 0.74 | 0.924 | 5 | 0.555 |  |  |  |  |  |  |  |  |  | |
| 2 | 57.10 | 1.90 | 57.75 | 3.40 | 0.936 | 5 | 0.636 |  |  |  |  |  |  |  |  |  | |
| 3 | 53.18 | 3.15 | 52.69 | 5.81 | 0.954 | 5 | 0.766 |  |  |  |  |  |  |  |  |  | |
| 3 80 kVp | 55.16 | 3.17 | 54.70 | 6.01 | 0.967 | 5 | 0.855 |  |  |  |  |  |  |  |  |  | |
| 4 | 3.92 | 0.33 | 3.91 | 0.58 | 0.884 | 5 | 0.329 |  |  |  |  |  |  |  |  |  | |
| 5 | 52.22 | 2.37 | 52.49 | 4.39 | 0.938 | 5 | 0.655 |  |  |  |  |  |  |  |  |  | |
| Phantom 1 recovery | | | | | | | | Phantom 1 recovery small VOI | | | | | | | |  | |
| S- | 1.20 | 0.92 | 0.79 | 1.78 | 0.861 | 5 | 0.231 | 0.71 | 0.21 | 0.83 | 0.36 | 0.815 | 5 | 0.106 | |  | |
| S | 5.38 | 2.30 | 5.59 | 4.30 | 0.992 | 5 | 0.986 | 0.97 | 0.26 | 1.01 | 0.40 | 0.871 | 5 | 0.271 | |  | |
| S+ | 6.31 | 2.73 | 6.50 | 5.06 | 0.996 | 5 | 0.995 | 1.02 | 0.28 | 1.05 | 0.43 | 0.900 | 5 | 0.412 | |  | |
| T100 | 0.29 | 0.20 | 0.30 | 0.41 | 0.878 | 5 | 0.300 | 0.29 | 0.20 | 0.30 | 0.41 | 0.878 | 5 | 0.300 | |  | |
| T1SD | 0.47 | 0.20 | 0.49 | 0.37 | 0.988 | 5 | 0.974 | 0.47 | 0.20 | 0.49 | 0.37 | 0.988 | 5 | 0.974 | |  | |
| T2SD | 0.47 | 0.20 | 0.49 | 0.37 | 0.988 | 5 | 0.974 | 0.47 | 0.20 | 0.49 | 0.37 | 0.988 | 5 | 0.974 | |  | |
| T3SD | 0.47 | 0.20 | 0.49 | 0.37 | 0.988 | 5 | 0.974 | 0.47 | 0.20 | 0.49 | 0.37 | 0.988 | 5 | 0.974 | |  | |
| T50 | 0.42 | 0.20 | 0.44 | 0.39 | 0.978 | 5 | 0.926 | 0.42 | 0.20 | 0.44 | 0.39 | 0.978 | 5 | 0.926 | |  | |
| T | 0.47 | 0.20 | 0.49 | 0.37 | 0.988 | 5 | 0.974 | 0.47 | 0.20 | 0.49 | 0.37 | 0.988 | 5 | 0.974 | |  | |
| Phantom 2 recovery | | | | | | | | Phantom 2 recovery small VOI | | | | | | | |  | |
| S- | 0.66 | 0.32 | 0.74 | 0.61 | 0.900 | 5 | 0.409 | 0.66 | 0.32 | 0.74 | 0.61 | 0.900 | 5 | 0.409 | |  | |
| S | 0.70 | 0.29 | 0.74 | 0.56 | 0.930 | 5 | 0.597 | 0.69 | 0.31 | 0.74 | 0.59 | 0.927 | 5 | 0.578 | |  | |
| S+ | 2.36 | 0.90 | 1.81 | 1.53 | 0.793 | 5 | 0.071 | 1.10 | 0.23 | 1.07 | 0.37 | 0.963 | 5 | 0.829 | |  | |
| T100 | 0.84 | 0.11 | 0.87 | 0.21 | 0.927 | 5 | 0.577 | 0.84 | 0.11 | 0.87 | 0.21 | 0.927 | 5 | 0.577 | |  | |
| T1SD | 4.13 | 2.37 | 3.54 | 3.27 | 0.726 | 5 | **0.017** | 1.56 | 0.39 | 1.60 | 0.62 | 0.892 | 5 | 0.369 | |  | |
| T2SD | 1.10 | 0.12 | 1.11 | 0.21 | 0.925 | 5 | 0.565 | 1.02 | 0.10 | 1.05 | 0.19 | 0.928 | 5 | 0.581 | |  | |
| T3SD | 0.94 | 0.09 | 0.98 | 0.16 | 0.898 | 5 | 0.397 | 0.94 | 0.09 | 0.98 | 0.16 | 0.898 | 5 | 0.397 | |  | |
| T50 | 20.42 | 14.40 | 14.75 | 20.57 | 0.775 | 5 | **0.050** | 4.39 | 2.57 | 3.42 | 4.70 | 0.944 | 5 | 0.693 | |  | |
| T | 14.21 | 9.55 | 9.73 | 14.77 | 0.799 | 5 | 0.079 | 3.27 | 1.75 | 2.62 | 3.33 | 0.896 | 5 | 0.391 | |  | |
| Phantom 3 recovery | | | | | | | | Phantom 3 recovery small VOI | | | | | | | |  | |
| S- | 0.88 | 0.45 | 0.98 | 0.77 | 0.971 | 5 | 0.883 | 0.76 | 0.41 | 0.89 | 0.72 | 0.903 | 5 | 0.424 | |  | |
| S | 1.93 | 1.15 | 1.78 | 2.12 | 0.955 | 5 | 0.776 | 0.98 | 0.48 | 1.07 | 0.77 | 0.838 | 5 | 0.158 | |  | |
| S+ | 3.28 | 1.15 | 3.34 | 1.94 | 0.960 | 5 | 0.810 | 1.20 | 0.35 | 1.25 | 0.61 | 0.908 | 5 | 0.457 | |  | |
| T100 | 0.78 | 0.22 | 0.88 | 0.31 | 0.683 | 5 | **0.006** | 0.78 | 0.22 | 0.88 | 0.31 | 0.683 | 5 | **0.006** | |  | |
| T1SD | 1.34 | 0.70 | 1.07 | 1.08 | 0.782 | 5 | 0.057 | 0.98 | 0.16 | 0.89 | 0.29 | 0.837 | 5 | 0.157 | |  | |
| T2SD | 0.79 | 0.17 | 0.83 | 0.29 | 0.909 | 5 | 0.460 | 0.78 | 0.18 | 0.83 | 0.30 | 0.903 | 5 | 0.426 | |  | |
| T3SD | 0.72 | 0.17 | 0.78 | 0.27 | 0.886 | 5 | 0.336 | 0.72 | 0.17 | 0.78 | 0.27 | 0.886 | 5 | 0.336 | |  | |
| T50 | 12.43 | 4.57 | 11.34 | 8.96 | 0.905 | 5 | 0.436 | 2.78 | 0.79 | 2.85 | 1.47 | 0.929 | 5 | 0.591 | |  | |
| T | 11.13 | 3.91 | 9.01 | 7.42 | 0.807 | 5 | 0.093 | 2.57 | 0.77 | 2.81 | 1.51 | 0.895 | 5 | 0.382 | |  | |
| Phantom 1 recovery 80 kVp | | | | | | | | Phantom 3 recovery 80 kVp | | | | | | | |  | |
| S- | 0.89 | 0.78 | 0.57 | 1.50 | 0.884 | 5 | 0.330 | 0.88 | 0.40 | 0.92 | 0.65 | 0.947 | 5 | 0.718 | |  | |
| S | 6.28 | 2.83 | 6.62 | 5.27 | 0.990 | 5 | 0.980 | 1.73 | 0.95 | 1.67 | 1.70 | 0.961 | 5 | 0.816 | |  | |
| S+ | 6.95 | 3.09 | 7.25 | 5.74 | 0.993 | 5 | 0.990 | 3.02 | 0.98 | 2.90 | 1.72 | 0.981 | 5 | 0.938 | |  | |
| T100 | 0.33 | 0.20 | 0.34 | 0.40 | 0.891 | 5 | 0.360 | 0.79 | 0.21 | 0.86 | 0.30 | 0.739 | 5 | **0.023** | |  | |
| T1SD | 1.95 | 0.67 | 2.02 | 1.20 | 0.955 | 5 | 0.776 | 1.68 | 1.01 | 1.44 | 1.78 | 0.877 | 5 | 0.296 | |  | |
| T2SD | 1.34 | 0.50 | 1.45 | 0.92 | 0.964 | 5 | 0.832 | 0.80 | 0.15 | 0.78 | 0.27 | 0.925 | 5 | 0.563 | |  | |
| T3SD | 0.76 | 0.24 | 0.88 | 0.36 | 0.745 | 5 | **0.027** | 0.71 | 0.17 | 0.74 | 0.29 | 0.926 | 5 | 0.568 | |  | |
| T50 | 0.49 | 0.20 | 0.49 | 0.36 | 0.992 | 5 | 0.986 | 15.18 | 5.73 | 13.90 | 11.21 | 0.912 | 5 | 0.480 | |  | |
| T | 1.95 | 0.67 | 2.02 | 1.20 | 0.955 | 5 | 0.776 | 13.16 | 4.59 | 10.69 | 8.71 | 0.819 | 5 | 0.115 | |  | |
| Phantom 4 recovery | | | | | | | | Phantom 5 recovery | | | | | | | |  | |
| S- | 0.81 | 0.07 | 0.81 | 0.12 | 0.833 | 5 | 0.146 | 0.25 | 1.30 | 0.73 | 2.12 | 0.825 | 5 | 0.127 | |  | |
| S | 2.67 | 0.83 | 2.43 | 1.30 | 0.907 | 5 | 0.450 | 1.91 | 0.50 | 1.90 | 0.86 | 0.965 | 5 | 0.844 | |  | |
| S+ | 2.91 | 0.86 | 2.65 | 1.40 | 0.929 | 5 | 0.591 | 3.08 | 0.51 | 2.86 | 0.75 | 0.757 | 5 | **0.034** | |  | |
| T100 | 0.59 | 0.18 | 0.53 | 0.34 | 0.899 | 5 | 0.402 | 0.90 | 0.18 | 0.88 | 0.36 | 0.868 | 5 | 0.260 | |  | |
| T1SD | 0.74 | 0.12 | 0.66 | 0.23 | 0.753 | 5 | **0.032** | 0.94 | 0.16 | 0.89 | 0.31 | 0.937 | 5 | 0.646 | |  | |
| T2SD | 0.74 | 0.12 | 0.66 | 0.23 | 0.753 | 5 | **0.032** | 0.84 | 0.16 | 0.78 | 0.31 | 0.879 | 5 | 0.304 | |  | |
| T3SD | 0.74 | 0.12 | 0.66 | 0.23 | 0.753 | 5 | **0.032** | 0.77 | 0.16 | 0.70 | 0.31 | 0.837 | 5 | 0.156 | |  | |
| T50 | 0.71 | 0.14 | 0.64 | 0.26 | 0.825 | 5 | 0.128 | 9.87 | 2.27 | 10.46 | 3.99 | 0.956 | 5 | 0.783 | |  | |
| T | 0.74 | 0.12 | 0.66 | 0.23 | 0.753 | 5 | **0.032** | 9.42 | 1.84 | 9.88 | 3.45 | 0.926 | 5 | 0.569 | |  | |
| Phantom 1 ho volume fraction | | | | | | | | Phantom 1 ho volume fraction small VOI | | | | | | | |  | |
| S- | 1.00 | 0.00 | 1.00 | 0.00 |  | 5 |  | 1.00 | 0.00 | 1.00 | 0.00 |  | 5 |  | |  | |
| S | 0.78 | 0.01 | 0.78 | 0.02 | 0.961 | 5 | 0.814 | 0.75 | 0.07 | 0.79 | 0.12 | 0.847 | 5 | 0.186 | |  | |
| S+ | 0.56 | 0.02 | 0.57 | 0.03 | 0.842 | 5 | 0.171 | 0.54 | 0.06 | 0.57 | 0.11 | 0.898 | 5 | 0.398 | |  | |
| T100 | 0.00 | 0.00 | 0.00 | 0.00 |  | 5 |  | 0.02 | 0.01 | 0.02 | 0.02 | 0.961 | 5 | 0.814 | |  | |
| T1SD | 0.00 | 0.01 | 0.00 | 0.01 | 0.684 | 5 | **0.006** | 0.08 | 0.02 | 0.07 | 0.04 | 0.867 | 5 | 0.254 | |  | |
| T2SD | 0.00 | 0.01 | 0.00 | 0.01 | 0.684 | 5 | **0.006** | 0.08 | 0.02 | 0.07 | 0.04 | 0.867 | 5 | 0.254 | |  | |
| T3SD | 0.00 | 0.01 | 0.00 | 0.01 | 0.684 | 5 | **0.006** | 0.08 | 0.02 | 0.07 | 0.04 | 0.867 | 5 | 0.254 | |  | |
| T50 | 0.00 | 0.00 | 0.00 | 0.00 |  | 5 |  | 0.04 | 0.02 | 0.04 | 0.03 | 0.987 | 5 | 0.967 | |  | |
| T | 0.00 | 0.01 | 0.00 | 0.01 | 0.684 | 5 | **0.006** | 0.08 | 0.02 | 0.07 | 0.04 | 0.867 | 5 | 0.254 | |  | |
| Phantom 2 ho volume fraction | | | | | | | | Phantom 2 ho volume fraction small VOI | | | | | | | |  | |
| S- | 1.00 | 0.00 | 1.00 | 0.00 |  | 5 |  | 1.00 | 0.00 | 1.00 | 0.00 |  | 5 |  | |  | |
| S | 1.00 | 0.00 | 1.00 | 0.00 |  | 5 |  | 1.00 | 0.00 | 1.00 | 0.01 | 0.552 | 5 | **0.000** | |  | |
| S+ | 0.56 | 0.04 | 0.56 | 0.09 | 0.950 | 5 | 0.734 | 0.53 | 0.13 | 0.51 | 0.27 | 0.899 | 5 | 0.404 | |  | |
| T100 | 0.01 | 0.01 | 0.01 | 0.01 | 0.684 | 5 | **0.006** | 0.03 | 0.02 | 0.03 | 0.03 | 0.779 | 5 | 0.054 | |  | |
| T1SD | 0.13 | 0.02 | 0.13 | 0.04 | 0.952 | 5 | 0.754 | 0.17 | 0.05 | 0.16 | 0.09 | 0.949 | 5 | 0.732 | |  | |
| T2SD | 0.01 | 0.01 | 0.01 | 0.01 | 0.684 | 5 | **0.006** | 0.06 | 0.02 | 0.06 | 0.05 | 0.895 | 5 | 0.384 | |  | |
| T3SD | 0.01 | 0.00 | 0.01 | 0.00 |  | 5 |  | 0.05 | 0.02 | 0.04 | 0.04 | 0.842 | 5 | 0.171 | |  | |
| T50 | 0.88 | 0.04 | 0.91 | 0.08 | 0.767 | 5 | **0.042** | 0.84 | 0.05 | 0.85 | 0.07 | 0.796 | 5 | 0.075 | |  | |
| T | 0.56 | 0.04 | 0.56 | 0.09 | 0.950 | 5 | 0.734 | 0.53 | 0.13 | 0.51 | 0.27 | 0.899 | 5 | 0.404 | |  | |
| Phantom 3 ho volume fraction | | | | | | | | Phantom 3 ho volume fraction small VOI | | | | | | | |  | |
| S- | 1.00 | 0.00 | 1.00 | 0.00 |  | 5 |  | 1.00 | 0.00 | 1.00 | 0.00 |  | 5 |  | |  | |
| S | 0.99 | 0.01 | 0.99 | 0.02 | 0.881 | 5 | 0.314 | 0.99 | 0.01 | 0.99 | 0.02 | 0.961 | 5 | 0.814 | |  | |
| S+ | 0.67 | 0.08 | 0.66 | 0.16 | 0.989 | 5 | 0.976 | 0.74 | 0.15 | 0.79 | 0.27 | 0.899 | 5 | 0.406 | |  | |
| T100 | 0.01 | 0.01 | 0.01 | 0.01 | 0.684 | 5 | **0.006** | 0.04 | 0.02 | 0.04 | 0.04 | 0.979 | 5 | 0.928 | |  | |
| T1SD | 0.04 | 0.05 | 0.01 | 0.08 | 0.701 | 5 | **0.010** | 0.09 | 0.05 | 0.08 | 0.10 | 0.919 | 5 | 0.523 | |  | |
| T2SD | 0.01 | 0.01 | 0.01 | 0.01 | 0.684 | 5 | **0.006** | 0.04 | 0.01 | 0.04 | 0.02 | 0.833 | 5 | 0.146 | |  | |
| T3SD | 0.00 | 0.01 | 0.00 | 0.01 | 0.684 | 5 | **0.006** | 0.03 | 0.01 | 0.03 | 0.02 | 0.881 | 5 | 0.314 | |  | |
| T50 | 0.77 | 0.03 | 0.77 | 0.05 | 0.964 | 5 | 0.833 | 0.82 | 0.06 | 0.83 | 0.10 | 0.930 | 5 | 0.599 | |  | |
| T | 0.67 | 0.08 | 0.66 | 0.16 | 0.989 | 5 | 0.976 | 0.74 | 0.15 | 0.79 | 0.27 | 0.899 | 5 | 0.406 | |  | |
| Phantom 4 ho volume fraction | | | | | | | | Phantom 4 ho volume fraction small VOI | | | | | | | |  | |
| S- | 1.00 | 0.00 | 1.00 | 0.00 |  | 5 |  | 1.00 | 0.00 | 1.00 | 0.00 |  | 5 |  | |  | |
| S | 0.77 | 0.01 | 0.78 | 0.02 | 0.735 | 5 | **0.021** | 0.73 | 0.02 | 0.73 | 0.05 | 0.895 | 5 | 0.384 | |  | |
| S+ | 0.55 | 0.05 | 0.58 | 0.09 | 0.835 | 5 | 0.152 | 0.51 | 0.03 | 0.51 | 0.06 | 0.995 | 5 | 0.994 | |  | |
| T100 | 0.00 | 0.00 | 0.00 | 0.01 | 0.552 | 5 | **0.000** | 0.02 | 0.01 | 0.01 | 0.02 | 0.735 | 5 | **0.021** | |  | |
| T1SD | 0.01 | 0.00 | 0.01 | 0.00 |  | 5 |  | 0.05 | 0.02 | 0.05 | 0.03 | 0.779 | 5 | 0.054 | |  | |
| T2SD | 0.01 | 0.00 | 0.01 | 0.00 |  | 5 |  | 0.05 | 0.02 | 0.05 | 0.03 | 0.779 | 5 | 0.054 | |  | |
| T3SD | 0.01 | 0.00 | 0.01 | 0.00 |  | 5 |  | 0.05 | 0.02 | 0.05 | 0.03 | 0.779 | 5 | 0.054 | |  | |
| T50 | 0.01 | 0.00 | 0.01 | 0.00 |  | 5 |  | 0.04 | 0.02 | 0.03 | 0.03 | 0.859 | 5 | 0.223 | |  | |
| T | 0.01 | 0.00 | 0.01 | 0.00 |  | 5 |  | 0.05 | 0.02 | 0.05 | 0.03 | 0.779 | 5 | 0.054 | |  | |
| Patient parameters | | | | | | | | | | | | | | | |  | |
| Ho-MS content | 19.68 | 0.26 | 19.74 | 0.51 | 0.932 | 7 | 0.566 |  | | | | | | | |  | |
| Spec. radioactivity | 5.00 | 3.12 | 3.12 | 3.90 | 0.889 | 7 | 0.270 |  |  |  |  |  |  |  |  |  | |
| Injections | 67.51 | 86.00 | 36.35 | 55.69 | 0.696 | 7 | **0.003** |  |  |  |  |  |  |  |  |  | |
| Injection efficacy | 1.25 | 1.06 | 1.21 | 0.81 | 0.765 | 7 | **0.018** |  |  |  |  |  |  |  |  |  | |
| Ho-MS conc. | 4.18 | 3.76 | 2.72 | 6.40 | 0.824 | 7 | 0.070 |  | | | | | | |  | |  |
| Patient volumes (mL) | | | | | | | | Patient radiodensity (HU) | | | | | | | |  | |
| Reference VOI | 20.18 | 31.82 | 4.55 | 35.30 | 0.661 | 7 | **0.001** | 49.49 | 10,90* | 48.42 | 4.96 | 0.891 | 7 | 0.280 | |  | |
| Quantification VOI | 40.60 | 69.15 | 7.44 | 61.94 | 0.634 | 7 | **0.001** | 75.52 | 94,52* | 71.73 | 14.01 | 0.928 | 7 | 0.535 | |  | |
| Patient recovery | | | | | | | | Patient ho volume fraction | | | | | | | |  | |
| S- | 1.44 | 1.31 | 0.99 | 1.53 | 0.962 | 7 | 0.838 | 1.00 | 0.00 | 1.00 | 0.00 |  | 7 |  | |  | |
| S | 1.60 | 1.20 | 0.99 | 1.92 | 0.910 | 7 | 0.394 | 0.99 | 0.03 | 1.00 | 0.03 | 0.636 | 7 | **0.001** | |  | |
| S+ | 1.84 | 1.06 | 1.16 | 1.86 | 0.804 | 7 | **0.045** | 0.73 | 0.18 | 0.78 | 0.36 | 0.947 | 7 | 0.704 | |  | |
| T100 | 2.96 | 2.00 | 2.28 | 2.44 | 0.854 | 7 | 0.132 | 0.71 | 0.18 | 0.78 | 0.28 | 0.941 | 7 | 0.651 | |  | |
| T1SD | 2.51 | 1.83 | 1.42 | 2.25 | 0.796 | 7 | **0.037** | 0.51 | 0.23 | 0.52 | 0.41 | 0.933 | 7 | 0.580 | |  | |
| T2SD | 1.85 | 1.11 | 1.19 | 1.88 | 0.850 | 7 | 0.123 | 0.32 | 0.18 | 0.30 | 0.33 | 0.942 | 7 | 0.653 | |  | |
| T3SD | 1.34 | 0.52 | 1.10 | 0.85 | 0.920 | 7 | 0.473 | 0.20 | 0.14 | 0.15 | 0.20 | 0.942 | 7 | 0.658 | |  | |
| T50 | 2.94 | 2.05 | 2.05 | 2.59 | 0.865 | 7 | 0.169 | 0.66 | 0.23 | 0.73 | 0.48 | 0.904 | 7 | 0.355 | |  | |
| T | 0.98 | 0.31 | 0.96 | 0.67 | 0.935 | 7 | 0.591 | 0.12 | 0.09 | 0.13 | 0.11 | 0.891 | 7 | 0.279 | |  | |
| **DIFFERENCES** | | | | | | | | | | | | | | | |  | |
|  |  |  |  |  |  |  |  |  |  |  |  |  |  |  |  |  | |
| No. | Descriptives | | | | Shapiro-Wilk | | | Levene's test | | Statistical test | | | | PST / IST / M / W | |  | |
|  | Mean | SD | Median | IQR | W | df | p | F | sig. | t | z | df | p |  |  |  | |
| Phantom injections (mg) | | | | | | | | | | | | | | | |  | |
| 1 vs 4 | -7.99 | 2.12 | -7.61 | 3.82 | 0.988 | 5 | 0.972 | 0.68 | 0.43 | -3.654 |  | 8 | **0.006** | **IST** | |  | |
| 3 vs 5 | -1.10 | 3.56 | -0.65 | 5.66 | 0.941 | 5 | 0.676 | 0.57 | 0.47 | -0.585 |  | 8 | 0.575 | **IST** | |  | |
| Phantom volume reference VOI pre | | | | | | | | | | | | | | | |  | |
| 1 vs 2 | 43.12 | 0.84 | 42.90 | 1.59 | 0.962 | 5 | 0.822 | 4.03 | 0.08 | 93.309 |  | 8 | **0.000** | **IST** | |  | |
| 1 vs 3 | 39.01 | 2.80 | 38.77 | 4.85 | 0.912 | 5 | 0.480 | 4.54 | 0.07 | 29.493 |  | 8 | **0.000** | **IST** | |  | |
| Phantom volume reference VOI post | | | | | | | | | | | | | | | |  | |
| 1 vs 2 | 42.67 | 2.23 | 42.45 | 4.20 | 0.939 | 5 | 0.658 | 7.70 | 0.02 | 47.883 |  | 8 | **0.000** | **IST** | |  | |
| 1 vs 3 | 38.51 | 3.72 | 37.82 | 6.29 | 0.971 | 5 | 0.883 | 0.09 | 0.77 | 28.222 |  | 8 | **0.000** | **IST** | |  | |
| Phantom volume quantification VOI | | | | | | | | | | | | | | | |  | |
| 1 vs 2 | 43.00 | 1.57 | 43.17 | 2.72 | 0.971 | 5 | 0.885 | 0.37 | 0.56 | 80.602 |  | 8 | **0.000** | **IST** | |  | |
| 1 vs 3 | 38.72 | 2.48 | 39.21 | 3.79 | 0.893 | 5 | 0.372 | 2.59 | 0.15 | 29.958 |  | 8 | **0.000** | **IST** | |  | |
| Phantom volume small quantification VOI | | | | | | | | | | | | | | | |  | |
| 1 vs 2 | 0.51 | 2.39 | 0.19 | 4.39 | 0.956 | 5 | 0.783 | 1.03 | 0.34 | 0.545 |  | 8 | 0.600 | **IST** | |  | |
| 1 vs 3 | 0.38 | 1.64 | 1.39 | 3.09 | 0.759 | 5 | **0.036** | NA | |  | -0.313 |  | 0.754 | **M** | |  | |
| Phantom Ho-MS concentration quantification VOI | | | | | | | | | | | | | | | |  | |
| 1 vs 4 | -0.15 | 0.04 | -0.15 | 0.07 | 0.999 | 5 | 1.000 | 1.04 | 0.34 | -3.800 |  | 8 | **0.005** | **IST** | |  | |
| 3 vs 5 | -0.08 | 0.16 | -0.04 | 0.26 | 0.930 | 5 | 0.600 | 0.33 | 0.58 | -0.873 |  | 8 | 0.408 | **IST** | |  | |
| Phantom radiodensity reference VOI pre vs post (HU) | | | | | | | | | | | | | | | |  | |
| 1 | -0.23 | 0.26 | -0.24 | -0.52 | 0.915 | 5 | 0.496 | NA | | -2.008 |  | 4 | 0.115 | PST | |  | |
| 1 80 kVp | -0.11 | 0.42 | -0.11 | -0.80 | 0.984 | 5 | 0.953 |  |  | -0.608 |  | 4 | 0.576 | PST | |  | |
| 2 | 12.38 | 0.89 | 11.98 | -1.64 | 0.857 | 5 | 0.216 |  |  | 31.027 |  | 4 | **0.000** | PST | |  | |
| 3 | 5.72 | 0.56 | 5.55 | -0.89 | 0.909 | 5 | 0.459 |  |  | 23.003 |  | 4 | **0.000** | PST | |  | |
| 3 80 kVp | 6.77 | 0.61 | 6.44 | -1.12 | 0.888 | 5 | 0.345 |  |  | 24.668 |  | 4 | **0.000** | PST | |  | |
| 4 | -0.06 | 0.08 | -0.05 | -0.12 | 0.895 | 5 | 0.385 |  |  | -1.737 |  | 4 | 0.157 | PST | |  | |
| 5 | 5.81 | 2.24 | 6.72 | -3.19 | 0.725 | 5 | **0.017** |  |  |  | -2.023 |  | **0.043** | W | |  | |
| Phantom radiodensity used reference VOI vs quantification VOI (HU) | | | | | | | | | | | | | | | |  | |
| 1 | -0.42 | 0.20 | -0.44 | -0.39 | 0.964 | 5 | 0.836 | NA | | -4.662 |  | 4 | **0.010** | PST | |  | |
| 1 80 kVp | -0.36 | 0.27 | -0.39 | -0.50 | 0.975 | 5 | 0.907 |  |  | -2.970 |  | 4 | **0.041** | PST | |  | |
| 2 | -1.49 | 1.14 | -1.31 | -1.89 | 0.953 | 5 | 0.756 |  |  | -2.913 |  | 4 | **0.044** | PST | |  | |
| 3 | -2.28 | 1.44 | -2.52 | -2.72 | 0.970 | 5 | 0.878 |  |  | -3.542 |  | 4 | **0.024** | PST | |  | |
| 3 80 kVp | -2.75 | 1.54 | -2.92 | -2.97 | 0.963 | 5 | 0.831 |  |  | -3.988 |  | 4 | **0.016** | PST | |  | |
| 4 | -1.08 | 0.28 | -1.15 | -0.55 | 0.914 | 5 | 0.492 |  |  | -8.548 |  | 4 | **0.001** | PST | |  | |
| 5 | -1.26 | 3.58 | -1.79 | -6.37 | 0.928 | 5 | 0.586 |  |  | -0.791 |  | 4 | 0.473 | PST | |  | |
| Phantom 1 recovery large vs small VOI | | | | | | | | | | | | | | | |  | |
| S- | 0.48 | 0.79 | 0.15 | 1.52 | 0.870 | 5 | 0.266 | NA | | 1.371 |  | 4 | 0.242 | PST | |  | |
| S | 4.42 | 2.11 | 4.51 | 3.93 | 0.990 | 5 | 0.981 |  |  | 4.677 |  | 4 | **0.009** | PST | |  | |
| S+ | 5.29 | 2.54 | 5.38 | 4.67 | 0.990 | 5 | 0.980 |  |  | 4.654 |  | 4 | **0.010** | PST | |  | |
| T100 | 0.00 | 0.00 | 0.00 | 0.00 |  |  |  |  |  |  |  |  |  |  | |  | |
| T50 | 0.00 | 0.00 | 0.00 | 0.00 |  |  |  |  |  |  |  |  |  |  | |  | |
| Phantom 2 recovery large vs small VOI | | | | | | | | | | | | | | | |  | |
| S- | 0.00 | 0.00 | 0.00 | 0.00 |  |  |  | NA | |  |  |  |  |  | |  | |
| S | 0.01 | 0.02 | 0.00 | 0.03 | 0.701 | 5 | **0.010** |  |  |  | -1.342 |  | 0.180 | W | |  | |
| S+ | 1.26 | 0.86 | 0.95 | 1.37 | 0.832 | 5 | 0.145 |  |  | 3.296 |  | 4 | **0.030** | PST | |  | |
| T100 | 0.00 | 0.00 | 0.00 | 0.00 |  |  |  |  |  |  |  |  |  |  | |  | |
| T1SD | 2.57 | 2.24 | 1.89 | 3.01 | 0.730 | 5 | **0.019** |  |  |  | -2.023 |  | **0.043** | W | |  | |
| T2SD | 0.08 | 0.08 | 0.06 | 0.14 | 0.856 | 5 | 0.214 |  |  | 2.116 |  | 4 | 0.102 | PST | |  | |
| T3SD | 0.00 | 0.00 | 0.00 | 0.00 |  |  |  |  |  |  |  |  |  |  | |  | |
| T50 | 16.04 | 12.26 | 12.94 | 16.88 | 0.763 | 5 | **0.039** |  |  |  | -2.023 |  | **0.043** | W | |  | |
| T | 10.94 | 8.25 | 8.51 | 12.14 | 0.793 | 5 | 0.070 |  |  | 2.966 |  | 4 | **0.041** | PST | |  | |
| Phantom 3 recovery large vs small VOI | | | | | | | | | | | | | | | |  | |
| S- | 0.13 | 0.12 | 0.11 | 0.17 | 0.855 | 5 | 0.212 | NA | | 2.396 |  | 4 | 0.075 | PST | |  | |
| S | 0.96 | 0.83 | 0.55 | 1.58 | 0.840 | 5 | 0.166 |  |  | 2.583 |  | 4 | 0.061 | PST | |  | |
| S+ | 2.08 | 0.92 | 2.20 | 1.59 | 0.874 | 5 | 0.282 |  |  | 5.080 |  | 4 | **0.007** | PST | |  | |
| T100 | 0.00 | 0.00 | 0.00 | 0.00 |  |  |  |  |  |  |  |  |  |  | |  | |
| T1SD | 0.36 | 0.74 | 0.00 | 0.91 | 0.607 | 5 | **0.001** |  |  |  | -1.342 |  | 0.180 | W | |  | |
| T2SD | 0.00 | 0.00 | 0.00 | 0.01 | 0.552 | 5 | **0.000** |  |  |  | -1.000 |  | 0.317 | W | |  | |
| T3SD | 0.00 | 0.00 | 0.00 | 0.00 |  |  |  |  |  |  |  |  |  |  | |  | |
| T50 | 9.65 | 3.91 | 8.91 | 7.70 | 0.869 | 5 | 0.263 |  |  | 5.526 |  | 4 | **0.005** | PST | |  | |
| T | 8.55 | 3.31 | 7.12 | 6.38 | 0.848 | 5 | 0.187 |  |  | 5.782 |  | 4 | **0.004** | PST | |  | |
| Phantom 1 recovery 80 vs 120 kVp | | | | | | | | | | | | | | | |  | |
| S- | 0.31 | 0.22 | 0.24 | 0.29 | 0.718 | 5 | **0.015** | NA | |  | -2.023 |  | **0.043** | W | |  | |
| S | 4.67 | 2.11 | 4.76 | 3.94 | 0.994 | 5 | 0.992 |  |  | -3.794 |  | 4 | **0.019** | PST | |  | |
| S+ | -0.64 | 0.37 | -0.75 | 0.68 | 0.959 | 5 | 0.801 |  |  | -3.918 |  | 4 | **0.017** | PST | |  | |
| T100 | -0.04 | 0.01 | -0.04 | 0.01 | 0.552 | 5 | **0.000** |  |  |  | -2.121 |  | **0.034** | W | |  | |
| T50 | -0.07 | 0.04 | -0.05 | 0.06 | 0.791 | 5 | 0.069 |  |  | -4.185 |  | 4 | **0.014** | PST | |  | |
| Phantom 3 recovery 80 vs 120 kVp | | | | | | | | | | | | | | | |  | |
| S- | 0.00 | 0.09 | -0.04 | 0.17 | 0.897 | 5 | 0.391 | NA | | 0.050 |  | 4 | 0.962 | PST | |  | |
| S | 0.20 | 0.22 | 0.11 | 0.42 | 0.853 | 5 | 0.204 |  |  | 2.110 |  | 4 | 0.102 | PST | |  | |
| S+ | 0.26 | 0.22 | 0.15 | 0.41 | 0.867 | 5 | 0.255 |  |  | 2.651 |  | 4 | 0.057 | PST | |  | |
| T100 | -0.01 | 0.04 | -0.01 | 0.07 | 0.978 | 5 | 0.925 |  |  | -0.550 |  | 4 | 0.611 | PST | |  | |
| T1SD | -0.34 | 0.35 | -0.37 | 0.70 | 0.900 | 5 | 0.410 |  |  | -2.142 |  | 4 | 0.099 | PST | |  | |
| T2SD | -0.01 | 0.05 | 0.00 | 0.09 | 0.969 | 5 | 0.870 |  |  | -0.642 |  | 4 | 0.556 | PST | |  | |
| T3SD | 0.01 | 0.03 | 0.01 | 0.06 | 0.995 | 5 | 0.994 |  |  | 0.399 |  | 4 | 0.710 | PST | |  | |
| T50 | -2.76 | 1.16 | -2.56 | 2.26 | 0.938 | 5 | 0.652 |  |  | -5.323 |  | 4 | **0.006** | PST | |  | |
| T | -2.03 | 0.69 | -1.70 | 1.30 | 0.870 | 5 | 0.265 |  |  | -6.564 |  | 4 | **0.003** | PST | |  | |
| Phantom recovery 1 vs 4 | | | | | | | | | | | | | | | |  | |
| S- | 0.39 | 0.89 | -0.02 | 1.72 | 0.819 | 5 | 0.115 | 36.60 | 0.00 | 0.930 |  | 4 | 0.404 | IST | |  | |
| S | 2.72 | 1.57 | 3.19 | 3.01 | 0.915 | 5 | 0.500 | 4.15 | 0.08 | 2.485 |  | 8 | **0.038** | IST | |  | |
| S+ | 3.40 | 1.95 | 3.91 | 3.69 | 0.958 | 5 | 0.792 | 4.35 | 0.07 | 2.653 |  | 8 | **0.029** | IST | |  | |
| T100 | -0.30 | 0.15 | -0.30 | 0.27 | 0.915 | 5 | 0.498 | 0.13 | 0.72 | -2.470 |  | 8 | **0.039** | IST | |  | |
| T50 | -0.28 | 0.14 | -0.29 | 0.25 | 0.971 | 5 | 0.880 | 0.76 | 0.41 | -2.584 |  | 8 | **0.032** | IST | |  | |
| Phantom recovery 3 vs 5 | | | | | | | | | | | | | | | |  | |
| S- | 0.63 | 1.15 | 0.25 | 1.52 | 0.717 | 5 | **0.014** | 2.91 | 0.13 |  | -0.629 |  | 0.530 | M | |  | |
| S | 0.02 | 1.42 | 0.21 | 2.20 | 0.826 | 5 | 0.131 | 2.99 | 0.12 | 0.043 |  | 8 | 0.967 | IST | |  | |
| S+ | 0.19 | 1.54 | 0.39 | 2.53 | 0.965 | 5 | 0.841 | 1.47 | 0.26 | 0.345 |  | 8 | 0.739 | IST | |  | |
| T100 | -0.12 | 0.29 | -0.18 | 0.54 | 0.933 | 5 | 0.620 | 0.03 | 0.88 | -0.911 |  | 8 | 0.389 | IST | |  | |
| T1SD | 0.40 | 0.79 | 0.20 | 1.38 | 0.872 | 5 | 0.276 | 3.16 | 0.11 | 1.239 |  | 8 | 0.250 | IST | |  | |
| T2SD | -0.05 | 0.25 | -0.20 | 0.47 | 0.816 | 5 | 0.110 | 0.03 | 0.86 | -0.500 |  | 8 | 0.631 | IST | |  | |
| T3SD | -0.05 | 0.23 | -0.18 | 0.44 | 0.847 | 5 | 0.186 | 0.11 | 0.75 | -0.518 |  | 8 | 0.619 | IST | |  | |
| T50 | 2.56 | 3.77 | 1.20 | 7.15 | 0.927 | 5 | 0.576 | 5.01 | 0.06 | 1.120 |  | 8 | 0.295 | IST | |  | |
| T | 1.71 | 3.93 | 0.95 | 7.34 | 0.907 | 5 | 0.447 | 11.35 | 0.01 | 0.882 |  | 6 | 0.413 | IST | |  | |
| Phantom 2 recovery quantification methods | | | | | | | | | | | | | | | |  | |
| Sm vs S | -0.04 | 0.04 | -0.05 | 0.07 | 0.879 | 5 | 0.304 | NA | | -2.216 |  | 4 | 0.091 | PST | |  | |
| T2SD vs T3SD | 0.16 | 0.10 | 0.12 | 0.17 | 0.845 | 5 | 0.179 |  |  | 3.584 |  | 4 | **0.023** | PST | |  | |
| T2SD vs T100 | 0.25 | 0.15 | 0.24 | 0.25 | 0.848 | 5 | 0.188 |  |  | 3.805 |  | 4 | **0.019** | PST | |  | |
| T3SD vs T100 | 0.10 | 0.06 | 0.08 | 0.10 | 0.880 | 5 | 0.311 |  |  | 3.900 |  | 4 | **0.018** | PST | |  | |
| S vs T3SD | -0.24 | 0.24 | -0.29 | 0.43 | 0.909 | 5 | 0.462 |  |  | -2.271 |  | 4 | 0.086 | PST | |  | |
| Phantom 3 recovery quantification methods | | | | | | | | | | | | | | | |  | |
| T2SD vs T3SD | 0.07 | 0.03 | 0.06 | 0.05 | 0.903 | 5 | 0.427 | NA | | 5.462 |  | 4 | **0.005** | PST | |  | |
| T2SD vs T100 | 0.00 | 0.08 | 0.01 | 0.16 | 0.931 | 5 | 0.603 |  |  | 0.108 |  | 4 | 0.919 | PST | |  | |
| T3SD vs T100 | -0.06 | 0.07 | -0.10 | 0.14 | 0.836 | 5 | 0.155 |  |  | -1.849 |  | 4 | 0.138 | PST | |  | |
| Sm vs T2SD | 0.10 | 0.39 | 0.03 | 0.68 | 0.933 | 5 | 0.619 |  |  | 0.557 |  | 4 | 0.607 | PST | |  | |
| Sm vs T100 | 0.10 | 0.32 | 0.07 | 0.54 | 0.853 | 5 | 0.205 |  |  | 0.692 |  | 4 | 0.527 | PST | |  | |
| Phantom 5 recovery quantification methods | | | | | | | | | | | | | | | |  | |
| T1SD vs T2SD | 0.11 | 0.03 | 0.11 | 0.05 | 0.940 | 5 | 0.666 | NA | | 7.385 |  | 4 | **0.002** | PST | |  | |
| T1SD vs T3SD | 0.17 | 0.04 | 0.17 | 0.07 | 0.978 | 5 | 0.925 |  |  | 9.358 |  | 4 | **0.001** | PST | |  | |
| T1SD vs T100 | 0.05 | 0.05 | 0.03 | 0.08 | 0.806 | 5 | 0.090 |  |  | 2.065 |  | 4 | 0.108 | PST | |  | |
| T2SD vs T3SD | 0.06 | 0.01 | 0.06 | 0.02 | 0.961 | 5 | 0.814 |  |  | 12.551 |  | 4 | **0.000** | PST | |  | |
| T2SD vs T100 | -0.06 | 0.04 | -0.06 | 0.07 | 0.915 | 5 | 0.501 |  |  | -3.795 |  | 4 | **0.019** | PST | |  | |
| T3SD vs T100 | -0.12 | 0.04 | -0.12 | 0.08 | 0.947 | 5 | 0.715 |  |  | -6.666 |  | 4 | **0.003** | PST | |  | |
| Phantom 1 recovery small VOI quantification methods | | | | | | | | | | | | | | | |  | |
| Sm vs S | -0.25 | 0.10 | -0.25 | 0.19 | 0.856 | 5 | 0.216 | NA | | -5.960 |  | 4 | **0.004** | PST | |  | |
| Sm vs Sp | -0.30 | 0.11 | -0.29 | 0.22 | 0.898 | 5 | 0.399 |  |  | -6.147 |  | 4 | **0.004** | PST | |  | |
| S vs Sp | -0.05 | 0.02 | -0.04 | 0.03 | 0.552 | 5 | **0.000** |  |  |  | -2.121 |  | **0.034** | W | |  | |
| Phantom 2 recovery small VOI quantification methods | | | | | | | | | | | | | | | |  | |
| Sm vs S | -0.03 | 0.03 | -0.04 | 0.05 | 0.766 | 5 | **0.041** | NA | |  | -1.633 |  | 0.102 | W | |  | |
| Sm vs Sp | -0.44 | 0.26 | -0.32 | 0.41 | 0.835 | 5 | 0.151 |  |  | -3.766 |  | 4 | **0.020** | PST | |  | |
| S vs Sp | -0.41 | 0.24 | -0.32 | 0.38 | 0.815 | 5 | 0.106 |  |  | -3.765 |  | 4 | **0.020** | PST | |  | |
| T2SD vs T3SD | 0.08 | 0.03 | 0.09 | 0.05 | 0.831 | 5 | 0.141 |  |  | 5.913 |  | 4 | 0.004 | PST | |  | |
| T2SD vs T100 | 0.17 | 0.08 | 0.17 | 0.15 | 0.982 | 5 | 0.945 |  |  | 4.910 |  | 4 | 0.008 | PST | |  | |
| T3SD vs T100 | 0.10 | 0.06 | 0.08 | 0.10 | 0.880 | 5 | 0.311 |  |  | 3.900 |  | 4 | 0.018 | PST | |  | |
| Sp vs T2SD | 0.08 | 0.16 | 0.10 | 0.29 | 0.913 | 5 | 0.485 |  |  | 1.105 |  | 4 | 0.331 | PST | |  | |
| Phantom 3 recovery small VOI quantification methods | | | | | | | | | | | | | | | |  | |
| Sm vs S | -0.22 | 0.20 | -0.16 | 0.38 | 0.888 | 5 | 0.348 | NA | | -2.480 |  | 4 | 0.068 | PST | |  | |
| Sm vs Sp | -0.44 | 0.21 | -0.52 | 0.34 | 0.878 | 5 | 0.302 |  |  | -4.747 |  | 4 | **0.009** | PST | |  | |
| S vs Sp | -0.22 | 0.17 | -0.24 | 0.31 | 0.896 | 5 | 0.387 |  |  | -2.881 |  | 4 | **0.045** | PST | |  | |
| T1SD vs T2SD | 0.19 | 0.12 | 0.18 | 0.24 | 0.958 | 5 | 0.796 |  |  | 3.533 |  | 4 | **0.024** | PST | |  | |
| T1SD vs T3SD | 0.26 | 0.13 | 0.29 | 0.25 | 0.950 | 5 | 0.735 |  |  | 4.390 |  | 4 | **0.012** | PST | |  | |
| T1SD vs T100 | 0.20 | 0.20 | 0.19 | 0.39 | 0.953 | 5 | 0.757 |  |  | 2.176 |  | 4 | 0.095 | PST | |  | |
| T2SD vs T3SD | 0.06 | 0.03 | 0.06 | 0.04 | 0.820 | 5 | 0.117 |  |  | 5.297 |  | 4 | **0.006** | PST | |  | |
| T2SD vs T100 | 0.00 | 0.08 | 0.01 | 0.15 | 0.931 | 5 | 0.605 |  |  | 0.056 |  | 4 | 0.958 | PST | |  | |
| T3SD vs T100 | -0.06 | 0.07 | -0.10 | 0.14 | 0.836 | 5 | 0.155 |  |  | -1.849 |  | 4 | 0.138 | PST | |  | |
| S vs T1SD | 0.00 | 0.43 | 0.16 | 0.71 | 0.903 | 5 | 0.428 |  |  | -0.010 |  | 4 | 0.992 | PST | |  | |
| S vs T100 | 0.19 | 0.29 | 0.16 | 0.54 | 0.899 | 5 | 0.404 |  |  | 1.483 |  | 4 | 0.212 | PST | |  | |
| Sp vs T1SD | 0.22 | 0.32 | 0.22 | 0.49 | 0.922 | 5 | 0.542 |  |  | 1.511 |  | 4 | 0.205 | PST | |  | |
| Sp vs T100 | 0.41 | 0.21 | 0.37 | 0.39 | 0.916 | 5 | 0.504 |  |  | 4.478 |  | 4 | **0.011** | PST | |  | |
| Phantom 3 recovery 80 kVp quantification methods | | | | | | | | | | | | | | | |  | |
| T2SD vs T3SD | 0.09 | 0.04 | 0.07 | 0.07 | 0.947 | 5 | 0.714 | NA | | 4.916 |  | 4 | **0.008** | PST | |  | |
| T2SD vs T100 | 0.01 | 0.10 | -0.01 | 0.19 | 0.884 | 5 | 0.327 |  |  | 0.172 |  | 4 | 0.872 | PST | |  | |
| T3SD vs T100 | -0.08 | 0.08 | -0.12 | 0.14 | 0.881 | 5 | 0.314 |  |  | -2.316 |  | 4 | **0.081** | PST | |  | |
| Sm vs T2SD | 0.08 | 0.35 | 0.07 | 0.60 | 0.958 | 5 | 0.797 |  |  | 0.508 |  | 4 | 0.638 | PST | |  | |
| Sm vs T100 | 0.09 | 0.28 | 0.06 | 0.46 | 0.877 | 5 | 0.297 |  |  | 0.711 |  | 4 | 0.516 | PST | |  | |
| Phantom 1 ho volume fraction large vs small VOI | | | | | | | | | | | | | | | |  | |
| S- | 0.00 | 0.00 | 0.00 | 0.00 |  | 5 |  | NA | |  |  |  |  |  | |  | |
| S | 0.04 | 0.06 | 0.01 | 0.11 | 0.867 | 5 | 0.256 |  |  | 1.298 |  | 4 | 0.264 | PST | |  | |
| S+ | 0.02 | 0.04 | 0.00 | 0.09 | 0.860 | 5 | 0.228 |  |  | 0.953 |  | 4 | 0.394 | PST | |  | |
| T100 | -0.02 | 0.01 | -0.02 | 0.02 | 0.961 | 5 | 0.814 |  |  | -3.138 |  | 4 | **0.035** | PST | |  | |
| T50 | -0.04 | 0.02 | -0.04 | 0.04 | 0.894 | 5 | 0.377 |  |  | -5.657 |  | 4 | **0.005** | PST | |  | |
| Phantom 2 ho volume fraction large vs small VOI | | | | | | | | | | | | | | | |  | |
| S- | 0.00 | 0.00 | 0.00 | 0.00 |  | 5 |  | NA | |  |  |  |  |  | |  | |
| S | 0.00 | 0.00 | 0.00 | 0.01 | 0.552 | 5 | **0.000** |  |  |  | -1.000 |  | 0.317 | W | |  | |
| S+ | 0.03 | 0.09 | 0.05 | 0.18 | 0.948 | 5 | 0.722 |  |  | 0.694 |  | 4 | 0.526 | PST | |  | |
| T100 | -0.03 | 0.02 | -0.02 | 0.03 | 0.859 | 5 | 0.223 |  |  | -2.982 |  | 4 | **0.041** | PST | |  | |
| T1SD | -0.05 | 0.04 | -0.04 | 0.07 | 0.956 | 5 | 0.783 |  |  | -2.482 |  | 4 | 0.068 | PST | |  | |
| T2SD | -0.05 | 0.02 | -0.05 | 0.04 | 0.833 | 5 | 0.146 |  |  | -4.311 |  | 4 | **0.013** | PST | |  | |
| T3SD | -0.04 | 0.02 | -0.03 | 0.04 | 0.806 | 5 | 0.090 |  |  | -3.882 |  | 4 | **0.018** | PST | |  | |
| T50 | 0.04 | 0.07 | 0.02 | 0.13 | 0.914 | 5 | 0.492 |  |  | 1.276 |  | 4 | 0.271 | PST | |  | |
| T | 0.03 | 0.09 | 0.05 | 0.18 | 0.948 | 5 | 0.722 |  |  | 0.694 |  | 4 | 0.526 | PST | |  | |
| Phantom 3 ho volume fraction large vs small VOI | | | | | | | | | | | | | | | |  | |
| S- | 0.00 | 0.00 | 0.00 | 0.00 |  | 5 |  | NA | |  |  |  |  |  | |  | |
| S | 0.00 | 0.01 | 0.00 | 0.02 | 0.961 | 5 | 0.814 |  |  | 0.343 |  | 4 | 0.749 | PST | |  | |
| S+ | -0.07 | 0.11 | -0.07 | 0.16 | 0.903 | 5 | 0.429 |  |  | -1.479 |  | 4 | 0.213 | PST | |  | |
| T100 | -0.04 | 0.02 | -0.03 | 0.03 | 0.859 | 5 | 0.223 |  |  | -5.308 |  | 4 | **0.006** | PST | |  | |
| T1SD | -0.06 | 0.04 | -0.06 | 0.07 | 0.950 | 5 | 0.738 |  |  | -2.889 |  | 4 | **0.045** | PST | |  | |
| T2SD | -0.04 | 0.01 | -0.04 | 0.01 | 0.552 | 5 | **0.000** |  |  |  | -2.060 |  | **0.039** | W | |  | |
| T3SD | -0.02 | 0.01 | -0.03 | 0.02 | 0.771 | 5 | **0.046** |  |  |  | -2.041 |  | **0.041** | W | |  | |
| T50 | -0.05 | 0.08 | -0.06 | 0.13 | 0.972 | 5 | 0.885 |  |  | -1.595 |  | 4 | 0.186 | PST | |  | |
| T | -0.07 | 0.11 | -0.07 | 0.16 | 0.903 | 5 | 0.429 |  |  | -1.479 |  | 4 | 0.213 | PST | |  | |
| Phantom 4 ho volume fraction large vs small VOI | | | | | | | | | | | | | | | |  | |
| S- | 0.00 | 0.00 | 0.00 | 0.00 |  | 5 |  | NA | |  |  |  |  |  | |  | |
| S | 0.04 | 0.03 | 0.04 | 0.05 | 0.944 | 5 | 0.692 |  |  | 3.508 |  | 4 | **0.025** | PST | |  | |
| S+ | 0.05 | 0.03 | 0.05 | 0.05 | 0.944 | 5 | 0.692 |  |  | 4.147 |  | 4 | **0.014** | PST | |  | |
| T100 | -0.02 | 0.01 | -0.01 | 0.02 | 0.735 | 5 | **0.021** |  |  |  | -2.060 |  | **0.039** | W | |  | |
| T50 | -0.03 | 0.02 | -0.02 | 0.03 | 0.859 | 5 | 0.223 |  |  | -3.255 |  | 4 | **0.031** | PST | |  | |
| Phantom ho volume fraction 1 vs 4 | | | | | | | | | | | | | | | |  | |
| S- | 0.00 | 0.00 | 0.00 | 0.00 |  | 5 |  |  |  |  |  |  |  |  | |  | |
| S | 0.01 | 0.02 | 0.01 | 0.04 | 0.943 | 5 | 0.685 | 0.04 | 0.85 | 1.549 |  | 8 | 0.160 | IST | |  | |
| S+ | 0.01 | 0.06 | 0.00 | 0.12 | 0.957 | 5 | 0.786 | 9.59 | 0.01 | 0.446 |  | 6 | 0.672 | IST | |  | |
| T100 | 0.00 | 0.00 | 0.00 | 0.00 |  | 5 |  | 7.11 | 0.03 | -1.000 |  | 4 | 0.374 | IST | |  | |
| T50 | -0.01 | 0.01 | -0.01 | 0.01 | 0.684 | 5 | **0.006** |  |  |  |  |  |  |  | |  | |
| Phantom ho volume fraction 1 vs 4 small VOI | | | | | | | | | | | | | | | |  | |
| S- | 0.00 | 0.00 | 0.00 | 0.00 |  | 5 |  |  |  |  |  |  |  |  | |  | |
| S | 0.02 | 0.05 | 0.02 | 0.09 | 0.908 | 5 | 0.457 | 11.28 | 0.01 | 0.571 |  | 5 | 0.592 | IST | |  | |
| S+ | 0.04 | 0.08 | 0.03 | 0.14 | 0.992 | 5 | 0.986 | 2.08 | 0.19 | 1.232 |  | 8 | 0.253 | IST | |  | |
| T100 | 0.00 | 0.01 | -0.01 | 0.02 | 0.771 | 5 | **0.046** | 0.04 | 0.85 |  | 0.000 |  | 1.000 | M | |  | |
| T50 | 0.01 | 0.01 | 0.00 | 0.02 | 0.771 | 5 | **0.046** | 0.06 | 0.81 |  | -0.319 |  | 0.750 | M | |  | |
| Patients Ho-MS concentration | | | | | | | | | | | | | | | |  | |
| 1-3 vs 4-7 | 5.71 | 2.98 |  |  |  |  |  | 4.40 | 0.09 | 3.114 |  | 5 | **0.026** | IST | |  | |
| Patients radiodensity VOIs | | | | | | | | | | | | | | | |  | |
| ref vs quant | -26.03 | 21.85 | 19.05 | 27.31 | 0.918 | 7 | 0.451 | NA | | -3.151 |  | 6 | **0.020** | PST | |  | |
| Patients recovery 1-3 vs 4-7 | | | | | | | | | | | | | | | |  | |
| S- | -0.86 |  |  |  |  |  |  | 3.20 | 0.13 | -0.84 |  | 5 | 0.44 | IST | |  | |
| S | -1.15 |  |  |  |  |  |  | 5.93 | 0.06 | -1.33 |  | 5 | 0.24 | IST | |  | |
| S+ | -1.34 |  |  |  |  |  |  | 7.80 | 0.04 | -2.40 |  | 3 | 0.09 | IST | |  | |
| T100 | -2.71 |  |  |  |  |  |  | 2.98 | 0.14 | -2.35 |  | 5 | 0.07 | IST | |  | |
| T1SD | -2.18 |  |  |  |  |  |  | 3.17 | 0.14 | -1.85 |  | 5 | 0.12 | IST | |  | |
| T2SD | -1.29 |  |  |  |  |  |  | 5.06 | 0.07 | -1.78 |  | 5 | 0.14 | IST | |  | |
| T3SD | -0.54 |  |  |  |  |  |  | 3.52 | 0.12 | -1.49 |  | 5 | 0.20 | IST | |  | |
| T50 | -2.77 |  |  |  |  |  |  | 2.31 | 0.19 | -2.35 |  | 5 | 0.07 | IST | |  | |
| T | -0.06 |  |  |  |  |  |  | 66.51 | 0.00 | -0.26 |  | 3 | 0.81 | IST | |  | |
| Patients Ho vol fraction 1-3 vs 4-7 | | | | | | | | | | | | | | | |  | |
| S- |  |  |  |  |  |  |  |  |  |  |  |  |  | IST | |  | |
| S | 0.02 |  |  |  |  |  |  | 6.70 | 0.05 | 1.51 |  | 3 | 0.23 | IST | |  | |
| S+ | -0.03 |  |  |  |  |  |  | 1.05 | 0.35 | -0.22 |  | 5 | 0.84 | IST | |  | |
| T100 | -0.01 |  |  |  |  |  |  | 0.39 | 0.56 | -0.09 |  | 5 | 0.93 | IST | |  | |
| T1SD | -0.01 |  |  |  |  |  |  | 2.46 | 0.18 | -0.04 |  | 5 | 0.97 | IST | |  | |
| T2SD | 0.05 |  |  |  |  |  |  | 0.40 | 0.55 | 0.35 |  | 5 | 0.74 | IST | |  | |
| T3SD | 0.12 |  |  |  |  |  |  | 0.48 | 0.52 | 1.15 |  | 5 | 0.30 | IST | |  | |
| T50 | -0.16 |  |  |  |  |  |  | 0.10 | 0.76 | -0.91 |  | 5 | 0.41 | IST | |  | |
| T | 0.13 |  |  |  |  |  |  | 1.54 | 0.27 | 2.43 |  | 5 | 0.06 | IST | |  | |
